# Heavy‐Atom Free Bodipy‐Borafluorene Photosensitizer Decorated with Coumarin Antenna Selectively Staining Endoplasmic Reticulum for Application in PDT

**DOI:** 10.1002/chem.202501949

**Published:** 2025-07-23

**Authors:** Karolina Wrochna, Dawid R. Natkowski, Agata Blacha‐Grzechnik, Sandra Pluczyk‐Malek, Connor B. Armstrong, Paolo J. Mastroeni, Dominic J. Black, Robert Pal, Krzysztof Durka, Paulina H. Marek‐Urban, Patrycja Stachelek

**Affiliations:** ^1^ Faculty of Chemistry Warsaw University of Technology Noakowskiego 3 Warsaw 00–664 Poland; ^2^ Faculty of Chemistry Silesian University of Technology Strzody 9 Gliwice 44–100 Poland; ^3^ Centre for Organic and Nanohybrid Electronics Silesian University of Technology Konarskiego 22B Gliwice 44–100 Poland; ^4^ Department of Chemistry Durham University South Road Durham DH1 3LE UK

**Keywords:** BODIPY, coumarin, endoplasmic reticulum, FRET, heavy‐atom free photosensitizers, PDT, spiro organoboron complexes

## Abstract

The organelle‐localized photodynamic therapy (PDT) has become a promising strategy for efficient cancer treatment. The photogeneration of reactive oxygen species in the endoplasmic reticulum (ER) leads to lipid oxidation and causes cell apoptosis or necrosis. However, most of the commercially available ER‐localized phototherapeutic agents exhibit dark cytotoxicity related to the presence of heavy atoms. In this contribution, we developed a novel heavy‐atom free BODIPY photosensitizer that localizes selectively in the ER with good biocompatibility, small dark cytotoxicity and efficient singlet oxygen generation, causing cellular death in a PDT experiment. The molecular design of the BODIPY PS involves two key elements: (i) replacing of the common BF2 group with the 9‐borafluorene which strongly enhances intersystem crossing to the triplet state responsible for photogeneration of singlet oxygen, and (ii) introduction of the pendant coumarin group ensuring ER‐localization and serving as light‐harvesting antenna for energy transfer to BODIPY core structure. The localization of BODIPY‐based PS was confirmed by laser scanning confocal microscopy (LSCM) imaging, and co‐staining revealed strong localization within the ER.

## Graphical abstract

A heavy‐atom free BODIPY photosensitizer localizing in the endoplasmic reticulum that efficiently generates singlet‐oxygen causing cell death upon light activation.

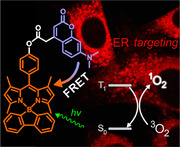

## 1 Introduction

Photodynamic therapy (PDT) is a clinically‐approved,‐edge treatment that has been applied for various cancers including skin, oesophagus, bladder, brain, oral, and lung cancers.^[^ ^1^, ^2^, ^3^, ^4^ ^]^ It involves non‐toxic photosensitizer molecules (PS) activated by light of a specific wavelength upon localization in tumorous cells.^[^ ^5^, ^6^ ^]^ The transition of electron or energy from triplet PS to molecular oxygen generates highly cytotoxic reactive oxygen species (ROS) that cause irreparable damage to cells. Since the lifetimes of ROS are relatively short (e.g., τ ≈ 0.1 ms in biological systems), their area of action is limited to the immediate vicinity of the photogeneration site (≈0.01 µm).^[^ ^7^ ^]^ For these reasons, PDT is a minimally‐invasive treatment that constitutes an interesting alternative to surgery, chemotherapy, or radiotherapy, reducing possible treatment repetition. The targeted localization of the PSs in cell organelles can further amplify the efficiency of PDT, reducing the required dose of PS and minimizing side effects.^[^ ^8^, ^9^, ^10^ ^]^ For instance, the accumulation of the PS in endoplasmic reticulum (ER) followed by the irradiation, leads to lipid oxidation, ER stress, and, in consequence, cell apoptosis or necrosis.^[^ ^11^, ^12^, ^13^, ^14^ ^]^

Among various groups of PS, boron dipyrromethenes (BODIPYs) are promising candidates for applications in PDT.^[^ ^15^, ^16^, ^17^, ^18^, ^19^, ^20^, ^21^, ^22^, ^23^, ^24^, ^25^, ^26^, ^27^ ^]^ This is due to their high molar extinction coefficient, excellent photo‐ and chemical stability, low dark cytotoxicity, and easy functionalization.^[^ ^28^, ^29^, ^30^, ^31^ ^]^ The primary design strategy for photosensitizing BODIPYs is to incorporate heavy atoms in the structures (e.g., Br, I),^[^ ^23^, ^32^, ^33^ ^]^ although such systems also show increased dark cytotoxicity, short triplet lifetime, and lower photostability.^[^ ^34^, ^35^ ^]^ In turn, the design of heavy‐atom free BODIPY PSs is usually challenging and lacks generalization.^[^ ^32^, ^35^, ^36^, ^37^, ^38^, ^39^ ^]^ In our recent works, we have developed a novel strategy for photosensitizing BODIPY without relying on the heavy atom effect, while simultaneously enabling facile functionalization toward desired properties.^[^ ^40^, ^41^, ^42^ ^]^ Specifically, we have found that the spiro organoboron BODIPY, featuring perpendicularly aligned 9‐borafluorene (BF) and dipyrromethene units, facilitates ISC to long‐living triplet state via so‐called spin‐orbit charge transfer intersystem crossing (SOCT‐ISC).^[^ ^37^, ^43^, ^44^, ^45^, ^46^, ^47^, ^48^ ^]^ However, the introduction of a 9‐borafluorene ring system increases hydrophobicity and reduces biocompatibility of the compound; thus for PDT applications it is crucial to counter this effect by introducing an organelle‐targeting functional group.

**Scheme 1:**
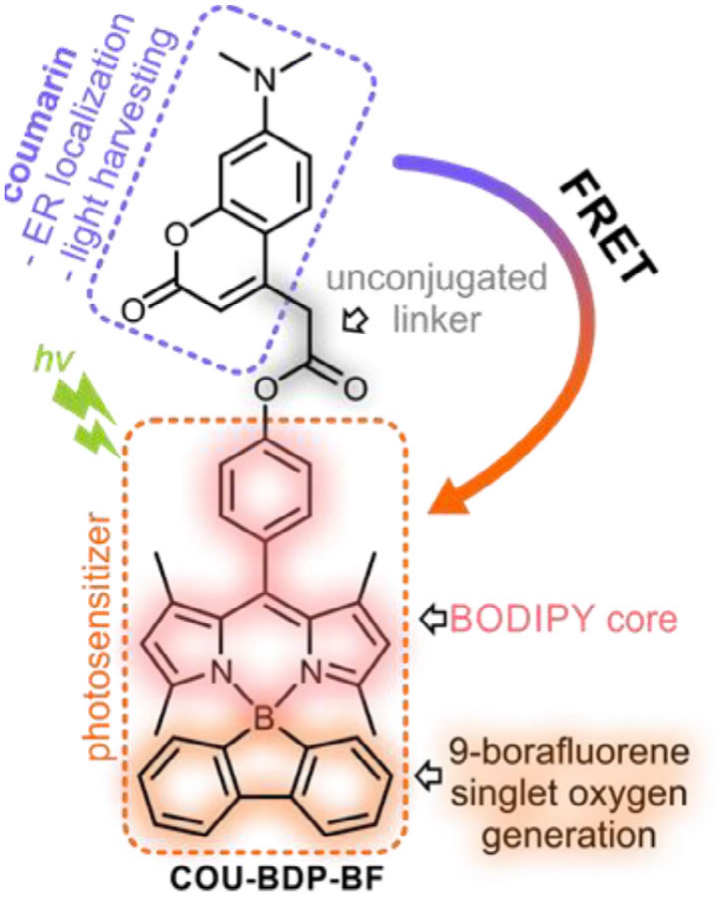
Coumarin‐BODIPY‐borafluorene (**COU‐BDP‐BF**) photosensitizer and referential compounds **ref‐COU** and **ref‐BDP‐BF**.

Herein, we present a coumarin‐BODIPY‐borafluorene triad (**COU‐BDP‐BF**) that selectively directs localization in the ER and further efficiently generates ROS upon excitation. The general idea of molecular design assumes the separation of molecular units responsible for the generation of singlet oxygen (9‐borafluorene) and the organelle‐targeting coumarin group (Scheme 1). Coumarin is a well‐recognized dye that has been used for bioimaging,^[^ ^49^ ^]^ in particular as ER‐selective probes.^[^ ^50^, ^51^, ^52^, ^53^ ^]^ Furthermore, due to the spectral overlap between coumarin emission and BODIPY absorption bands, it serves as a FRET energy donor to the BODIPY system.^[^ ^49^, ^54^ ^]^ The full mechanism of fluorescent probes localization in ER is not yet fully understood, however it is generally accepted that modifications like increasing probe's lipophilicity (*C*log*P* > 3.4), D‐π‐A substrates as well as amphipathic substituents direct probes to ER. This is because amphipathic and lipophilic molecules will generally be directed to the ER through passive diffusion through the cell membrane and competitive uptake by other organelles will be reduced. In our design coumarin also serves a role of increasing the solubility of the otherwise moderately soluble **BDP‐BF** in polar solvents.^[^ ^55^, ^56^ ^]^ To ensure efficient triplet state conversion, we replaced the conventional difluoroboron moiety (BF_2_) with an annulated 9‐borafluorene ring system. Specifically, the location of the HOMO orbital on the 9‐borafluorene moiety facilitates the photoinduced electron transfer (PeT) leading to the formation of a charge transfer state which further interconverts to the triplet state via SOCT‐ISC. Importantly, due to the orthogonal orientation of dipyrromethene and borafluorene moieties, such modification barely affects the absorption properties of BODIPY. Similarly, the connection of the coumarin unit via a non‐conjugated linker has only a marginal impact on BODIPY properties. Photobiological studies demonstrate that PS localizes in the endoplasmic reticulum and upon excitation generates ROS, resulting in cell necrosis. The obtained results are discussed in comparison to the two reference compounds, **ref‐COU** and **ref‐BDP‐BF**, representing the separate building units of the **COU‐BDP‐BF**, confirming the effectiveness of the proposed concept.

## 2 Results and Discussion

**Figure 1:**
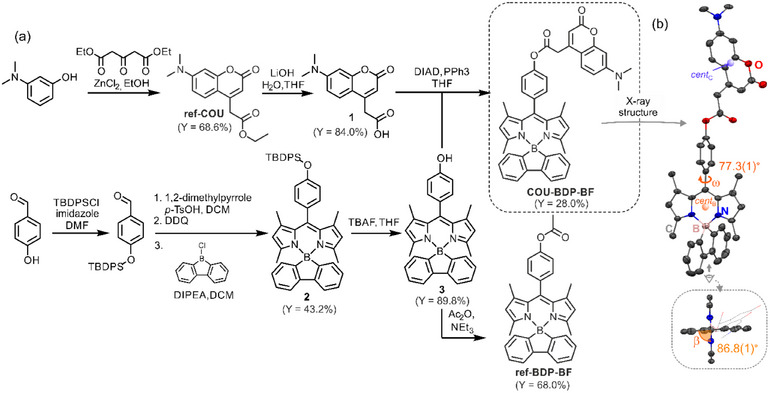
a) Synthesis of **COU‐BDP‐BF** and referential systems **ref‐COU**, **ref‐BDP‐BF**. b) Molecular structure of **COU‐BPD‐BF**, thermal ellipsoids were shown at 50% of probability, and hydrogen atoms were omitted for clarity.

The synthesis of **COU‐BDP‐BF** started from the preparation of BODIPY‐borafluorene complex functionalized with a hydroxyl group at the 4′‐position of the meso‐phenyl group (**3**). Thus 4‐(diphenyl‐*t*‐butylsilyl)oxybenzaldehyde was subjected to condensation with 2,4‐dimethylpyrrole followed by complexation with 9‐chloroborafluorene, giving BODIPY complex **2**. Subsequent desilylation with tetrabutylammonium fluoride (TBAF) afforded **3**. In parallel, coumarin was prepared following von Pechmann condensation from 3‐(dimethyloamino)phenol and 1,3‐acetonedicarboxylate yielding **ref‐COU**, which was further hydrolyzed to 1. Coupling compound **3** with **1** under the Mitsunobu conditions yielded the final **COU‐BDP‐BF** as an orange powder (Y: 28.0%), stable in air (Figure 1a). It also demonstrates sufficient photostability under strong irradiation and ROS concentration with an estimated half‐decomposition time of 18.5 h (Figures S7 and S8, Supporting Information), which is comparable to the 5,10,15,20‐tetraphenylporphyrin (TPP) photocatalysts (t_½_ = 16 h) measured under the same conditions. The reference compounds, **ref‐COU** and **ref‐BDP‐BF**, representing the separate building units of the **COU‐BDP‐BF**, were obtained for further comparative studies. All new compounds were fully characterized with ^1^H, ^11^B, ^13^C NMR spectroscopy, HR‐MS spectrometry, and single‐crystal X‐ray diffraction. Assignment of signals to individual proton groups in **COU‐BDP‐BF** was carried out using ^1^H‐^1^H COSY and ^1^H‐^13^C HSQC 2‐dimensional NMR spectroscopy (Figures S35 and S36, Supporting Information).

The structural analysis of **COU‐BDP‐BF** revealed that the adjacent borafluorene, dipyrromethene and meso‐phenyl moieties adopt nearly orthogonal orientation. The comparison between **ref‐BDP‐BF** and **COU‐BDP‐BF** proves that introduction of coumarin does not influence the molecular structure. The distance between coumarin (*cent_C_*, Figure 1b) and dipyrromethene (*cent_B_*) centroids is 12.17 Å, whereas the FRET critical distance (R_0_) was estimated as 55.8 Å (typical of FRET) assuming κ^2^ to be 2/3, this alongside good spectral overlap of 4.6 × 10^−14^ M^−1^cm^3^ in ethyl acetate provides further evidence for very efficient FRET.^[^ ^57^ ^]^

**Figure 2:**
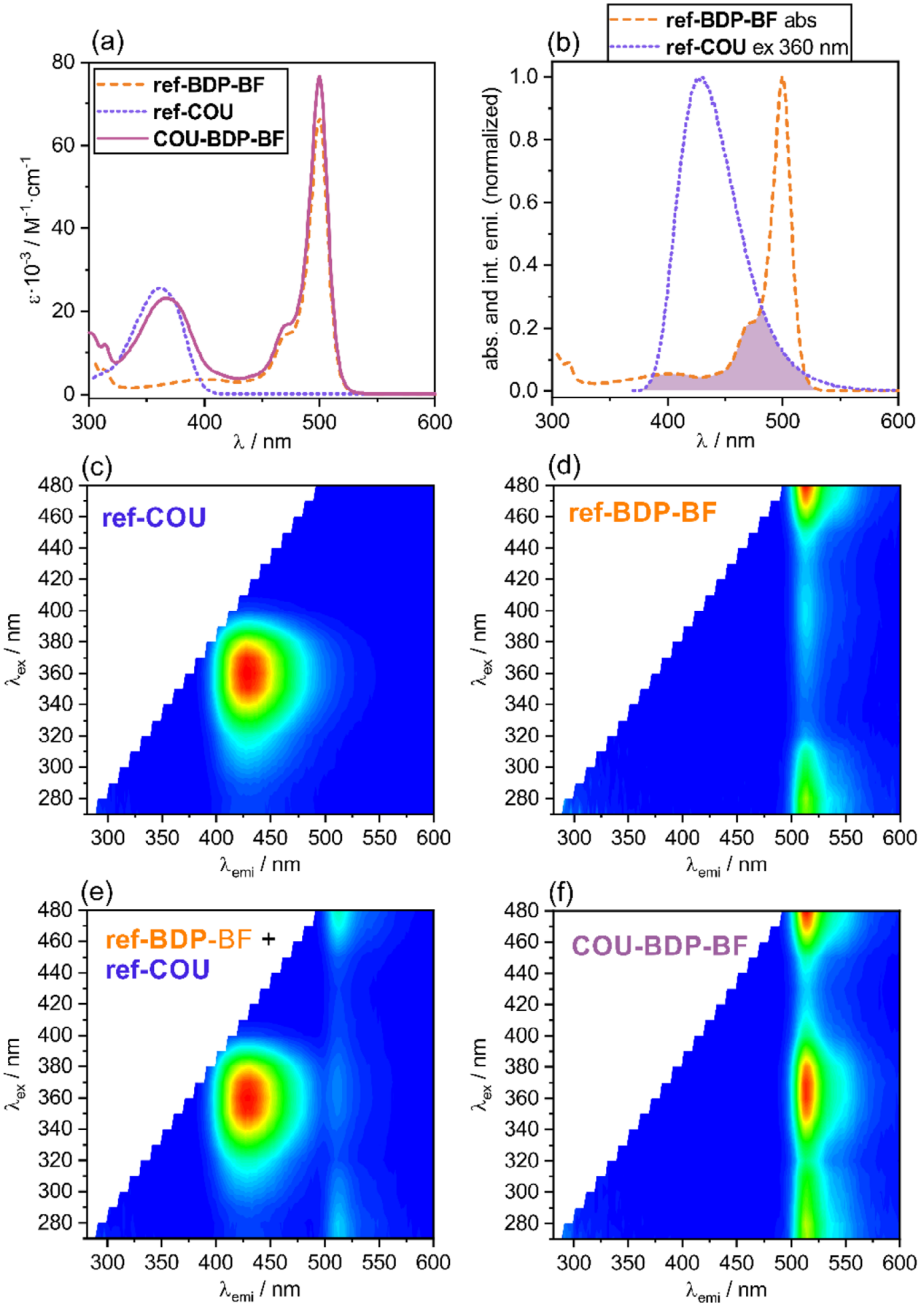
a) Absorption spectra of **COU‐BDP‐BF**, **ref‐COU,** and **ref‐BDP‐BF**. b) Overlay of **ref‐BDP‐BF** absorption and **ref‐COU** emission. Emission spectra as function of excitation wavelength for c) **ref‐COU**, d) **ref‐BDP‐BF**, e) equimolar mixture of **ref‐BDP‐BF** + **ref‐COU**, and f) **COU‐BDP‐BF**. All measurements were performed in AcOEt (C = 10^−5^ m).

Due to the orthogonal orientation of neighboring subunits, the coumarin and borafluorene groups have only a marginal effect on the absorption and emission properties of the central BODIPY chromophore (Table 1). Specifically, the absorption spectrum of **COU‐BDP‐BF** is a sum of the absorption bands of its building blocks (Figure 2a). Whereas, emission of the dyad, regardless of the excitation wavelength, corresponds exclusively to the narrow‐band emission (FWHM = 26 nm, λ_emi_ = 514 nm) of the BODIPY fragment, typical for BODIPY complexes including **ref‐BDP‐BF** (FWHM = 25 nm, λ_emi_ = 514 nm). Furthermore, the fluorescence quantum yield for both **COU‐BDP‐BF** (18%) and **ref‐BDP‐BF** (24%) complexes is relatively low and comparable to the parent (unmodified) BODIPY‐borafluorene complex,^[^ ^18^ ^]^ indicating possible depopulation of the singlet state via ISC. Excitation of the **COU‐BDP‐BF** in the 320–390 nm coumarin absorption range results in significant enhancement of the BODIPY emission intensity (Figure 2f) compared to the reference **ref‐BDP‐BF** complex (Figure 2d). To exclude that re‐absorption was responsible for the observed effect, emission spectra were recorded as a function of the excitation wavelength for the equimolar mixture of **ref‐BDP‐BF** and **ref‐COU** at the same concentration (10^−5^ m) (Figure 2e). The emission spectrum of the mixture is a sum of its components (Figure 2c,d) across all excitation ranges, clearly indicating quantitative energy transfer between coumarin and BODIPY units. The emission band associated with coumarin in **COU‐BDP‐BF** is barely visible, being c.a. 150 times less intense than the corresponding band in **ref‐COU**. In line with these observations, the calculated efficiency of the FRET is over 97%. The fluorescence decay of **COU‐BDP‐BF** at 514 nm measured by excitation with 340 nm EPLED laser source revealed mono‐exponential characteristics in ethyl acetate. The lifetime of 1.8 ns is closely comparable to the value record for **BDP‐BF** in ethyl acetate solution (τ = 1.7 ns). In contrast the lifetime of the **COU** unit in the **COU‐BDP‐BF** is too short to be resolved by the conventional time‐correlated, single photon counting methodology.

**Table 1:** Photophysical properties of studied systems in diluted DCM solution.

	λ_abs_ / nm (ε / M^−1^·cm^−1^)	λ_emi_ / nm	τ / ns	Φ^F^ / %	Φ_Δ_ / %(λ_ex_ / nm)
**ref‐COU**	361 (25 500)	428	3.33	97	0 (375)
**ref‐BDP‐BF**	499 (66 700)	514	1.66	24	49 (505)
**COU‐BDP‐Bf**	368 (23 200) 499 (76 600)	430 514	−1.76	−18^[^ ^a^ ^]^ / 31^[^ ^b^ ^]^ / 24^[^ ^c^ ^]^	61 (375) 57^[^ ^a^ ^]^ / 61^[^ ^b^ ^]^ / 84^[^ ^c^ ^]^ (505)

^a^ DCM

^b^Toluene

^c^DMF

**Figure 3:**
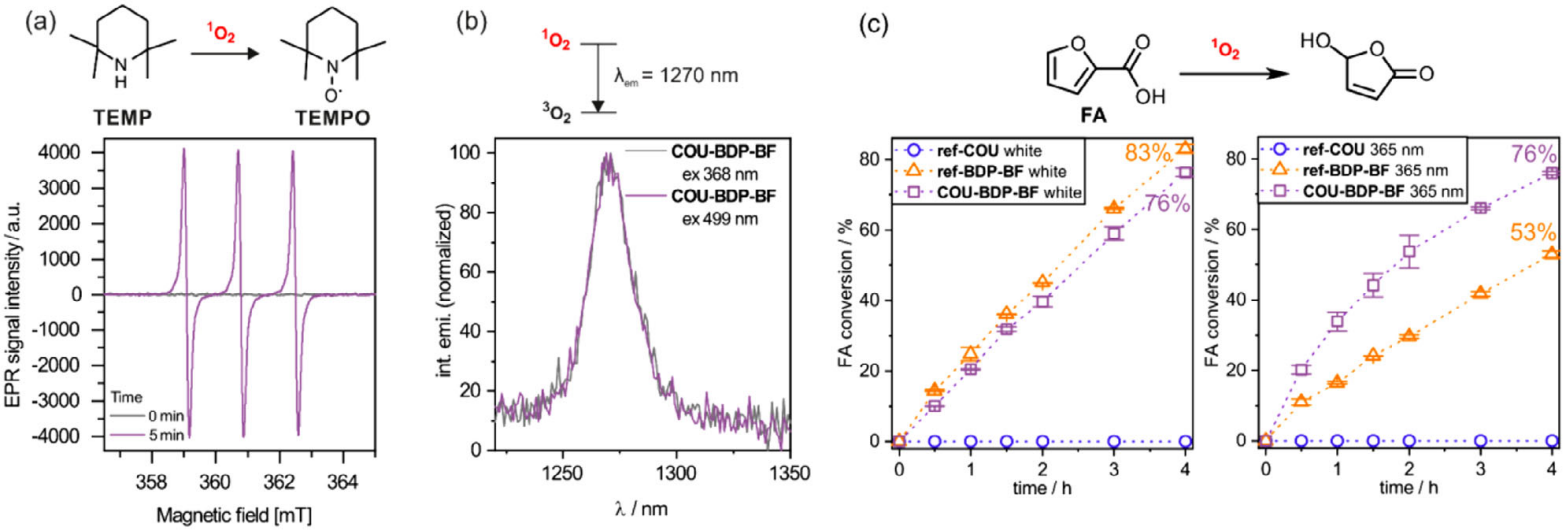
a) EPR spectrum recorded for **COU‐BDP‐BF** with TEMP as ¹O_2_ trap upon 5 min irradiation (EPR spectra for remaining systems are shown in Figure S5, Supporting Information). b) Singlet oxygen emission spectra were measured for **COU‐BDP‐BF** upon excitation at 368 nm and 499 nm. c) Reaction profiles for FA oxidation upon irradiation with white light and UV 365 nm.

In the next step, we evaluated the photocatalytic activity of **ref‐BDP‐BF** and **COU‐BDP‐BF**. The formation of singlet oxygen was first confirmed by EPR spectroscopy with diamagnetic 2,2,6,6‐tetramethylpiperidine (TEMP) as a ^1^O_2_ probe (Figure 3a). The formation of the paramagnetic nitroxide radical, TEMPO, was observed immediately after illumination with light. The g‐factor of the recorded spectra was 2.006, which aligns with the experimental data reported for TEMPO. The emission from singlet oxygen at 1270 nm was also directly observed with a spectrofluorometer equipped with an NIR detector (Figure 3b). In the following step, the singlet oxygen quantum yield (Φ_Δ_) was determined by monitoring the drop in intensity of the absorption band of 2,3,4,5‐tetraphenylcyclopentadienone (excitation at 375 nm) and diphenylisobenzofuran (excitation at 505 nm) – a well‐known singlet oxygen traps. According to our results, reference system **ref‐BDP‐BF** shows a moderate Φ_Δ_ of 49% (irradiated at 505 nm, DCM). In the case of **COU‐BDP‐BF**, Φ_Δ_ = 57% at 505 nm irradiation, and slightly increases to 61% when irradiated at 375 nm (DCM). The measured singlet oxygen quantum yields are comparable to the values obtained for other BODIPY‐borafluorene complexes studied in our previous work.^[^ ^42^ ^]^ The fluorescence and singlet oxygen quantum yields were also measured in toluene and dimethylformamide (DMF) selected as representative non‐polar and polar solvents, respectively. The results indicate that these parameters depend on the used solvent; however, there is no clear correlation with solvent polarity, and the observed variations are in relatively narrow range (Table 1). Beneficently, singlet oxygen quantum yield remains high independently on the used solvent, even reaching 84% in DMF. This suggests that similarly high yields may also be expected in biological environment.

Finally, BODIPY complexes were tested as singlet oxygen photosensitizers in the in situ oxidation of 2‐furoic acid (FA). The photocatalysts were used at only 0.05 molar % loading (*C* = 5.4 × 10^−5^ m), and irradiated with neutral‐white light (LED, 26 W) or 365 nm LED (26 W) (Figure 3c). Upon 4 h irradiation with white light, FA conversion reached 83% for **ref‐BDP‐BF** and 76% for **COU‐BDP‐BF** corresponding to TOF values of 416 and 378 h^−1^, respectively. In turn, upon irradiation at coumarin absorption wavelength (365 nm), **COU‐BDP‐BF** maintained at the same FA conversion rate (76%), whereas **ref‐BDP‐BF** exhibited a significant decrease (53%). In summary, these experiments confirm that coumarin efficiently transfers energy to the BODIPY subunit, which subsequently undergoes intersystem crossing to the triplet state, efficiently generating ^1^O_2_ in contact with oxygen. The incorporation of the coumarin unit does not interfere with the intersystem crossing mechanism operating in the BODIPY‐borafluorene subunits.

**Figure 4:**
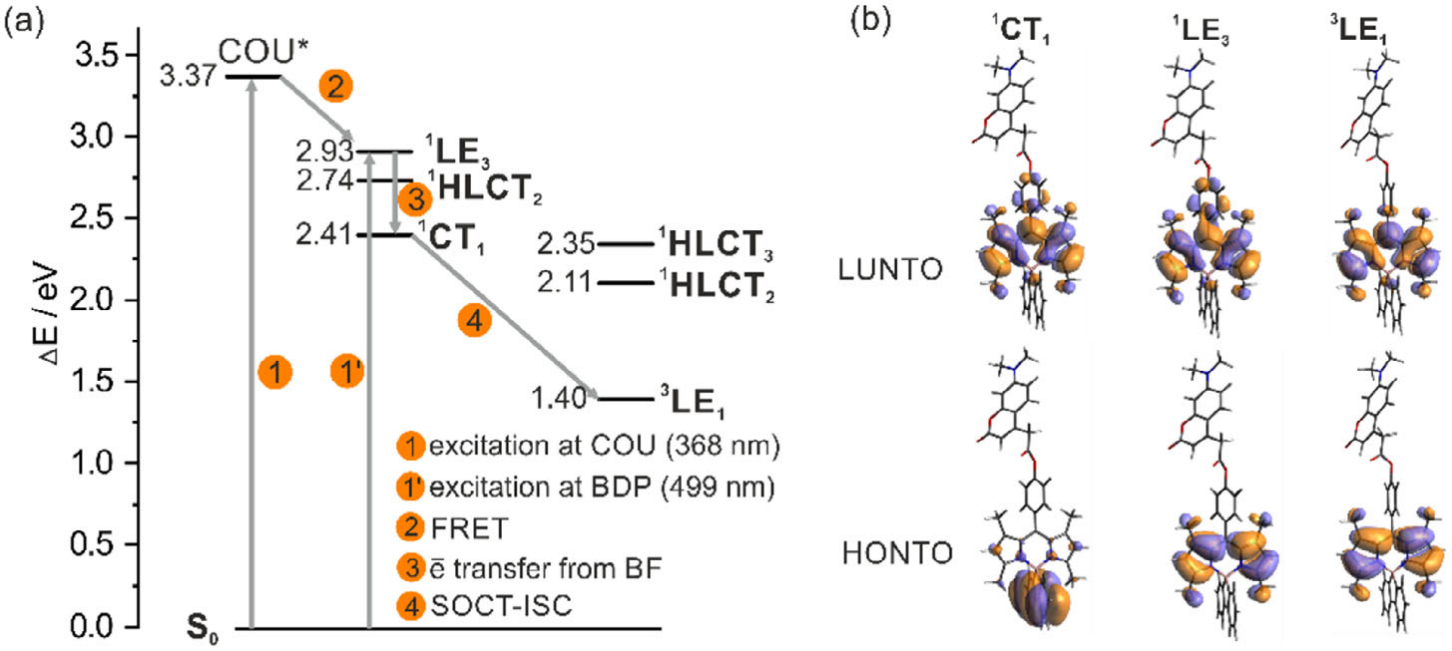
a) Calculated energy diagram demonstrating the photophysical processes in **COU‐BDP‐BF**. b) Visualization of natural transition orbitals (NTO) for key excited states in **COU‐BDP‐BF**.

To elucidate the mechanism underlying the observed photocatalytic activity of the **ref‐BDP‐BF** and **COU‐BDP‐BF** complexes, we conducted a series of TD‐DFT calculations at the PBE1PBE/6–311++G(d,p) level of theory. Natural transition orbital (NTO) analysis was used to characterize the nature of the excited states. According to our calculations, the lowest singlet excited state possesses charge transfer character (^1^CT_1_), a second singlet excited state displaying hybrid local‐charge transfer character (^1^HLCT_2_), and a third state is solely localized on the dipyrromethene moiety (^1^LE_3_). Furthermore, the calculations revealed the presence of a low‐energy triplet state (1.4 eV) localized on dipyrromethene (^3^LE_1_). In line with our experimental results, the coumarin does not participate in the photoactivation process as it does not affect the energy or spatial locations of the orbitals in the excited state. Upon the coumarin excitation at 368 nm, the energy is transferred to BODIPY via FRET. The further fate of the excited state is the same as observed for direct excitation of dipyrromethene at 499 nm. Specifically, the electron transfer from HOMO orbital located at the borafluorene unit leads to the population of ^1^CT_1_ singlet charge transfer state with positive and negative charges separated between the borafluorene and dipyrromethene subunits, respectively. Due to the orthogonal orientation of borafluorene and dipyrromethene moieties, the following ^1^CT_1_ → ^3^LE_1_ intersystem crossing is likely facilitated via SOCT‐ISC mechanism (Figure 4). It should be noted that SOCT‐ISC mechanism also operates in related *meso*‐functionalized BODIPY complexes.^[^ ^36^, ^37^, ^43^, ^58^, ^59^, ^60^, ^61^ ^]^ However, in our design, the donor unit is localized on the borafluorene moiety, thereby leaving the *meso* position available for further modifications. Concordantly, herein we have demonstrated that such molecular design is more efficient for obtaining complex molecular systems, while preserving their high photocatalytic activity.

**Figure 5:**
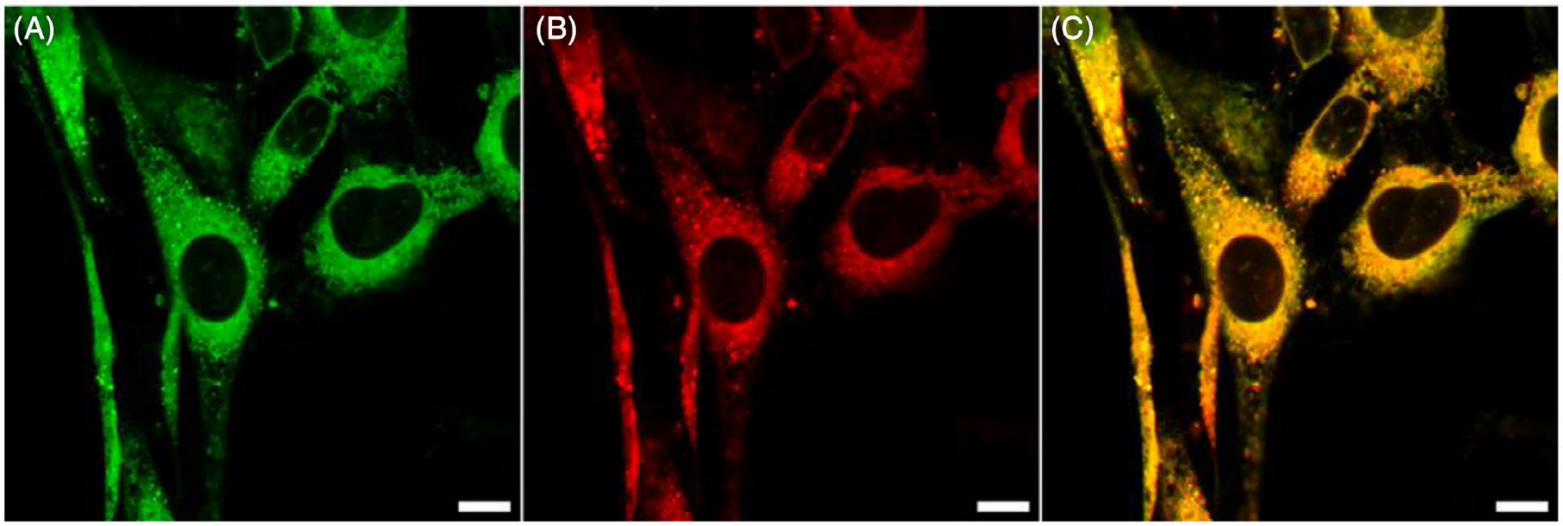
Images of mouse embryonic fibroblast (NIH‐3T3) cells. A) Image of **COU‐BDP‐BF** (λ_ex_ = 488 nm, 3 mW, λ_emi_ = 500–550 nm). B) ER TrackerTM Red (BODIPY™ TR Glibenclamide) (λ_ex_ = 543 nm, 36 mW, λ_emi_ = 550–750 nm). C) RGB merge of A and B. Pearson's coefficient calculated to be 0.94. Scale bars = 10 µm.

In order to test the validity of our novel PDT material design, we have incubated the Swiss mouse embryonic fibroblast cell line, NIH‐3T3, with 5 µM **COU‐BDP‐BF**. This cell line was selected for live‐cell laser scanning confocal microscopy (LSCM) as it offers reproducible results representative of and applicable to most mammalian cells. Co‐staining experiments showed that **COU‐BDP‐BF** localized within the endoplasmic reticulum with a high Pearson's correlation coefficient of 0.94 (Figure 5). Confocal imaging was performed after incubation in the dark for 2 h. In addition, co‐staining experiments were carried out with LysoTracker Red DND‐99 (Figure S9, Supporting Information) and MitoTracker Red (Figure S10, Supporting Information) to ascertain that **COU‐BDP‐BF** localizes exclusively in the ER. Localization of PDT material in the ER is desirable because high levels of ER stress can lead to programmed cell death and contribute toward anti‐cancer effects, making it especially valuable as a target for PDT therapies.^[^ ^25^ ^]^

**Figure 6:**
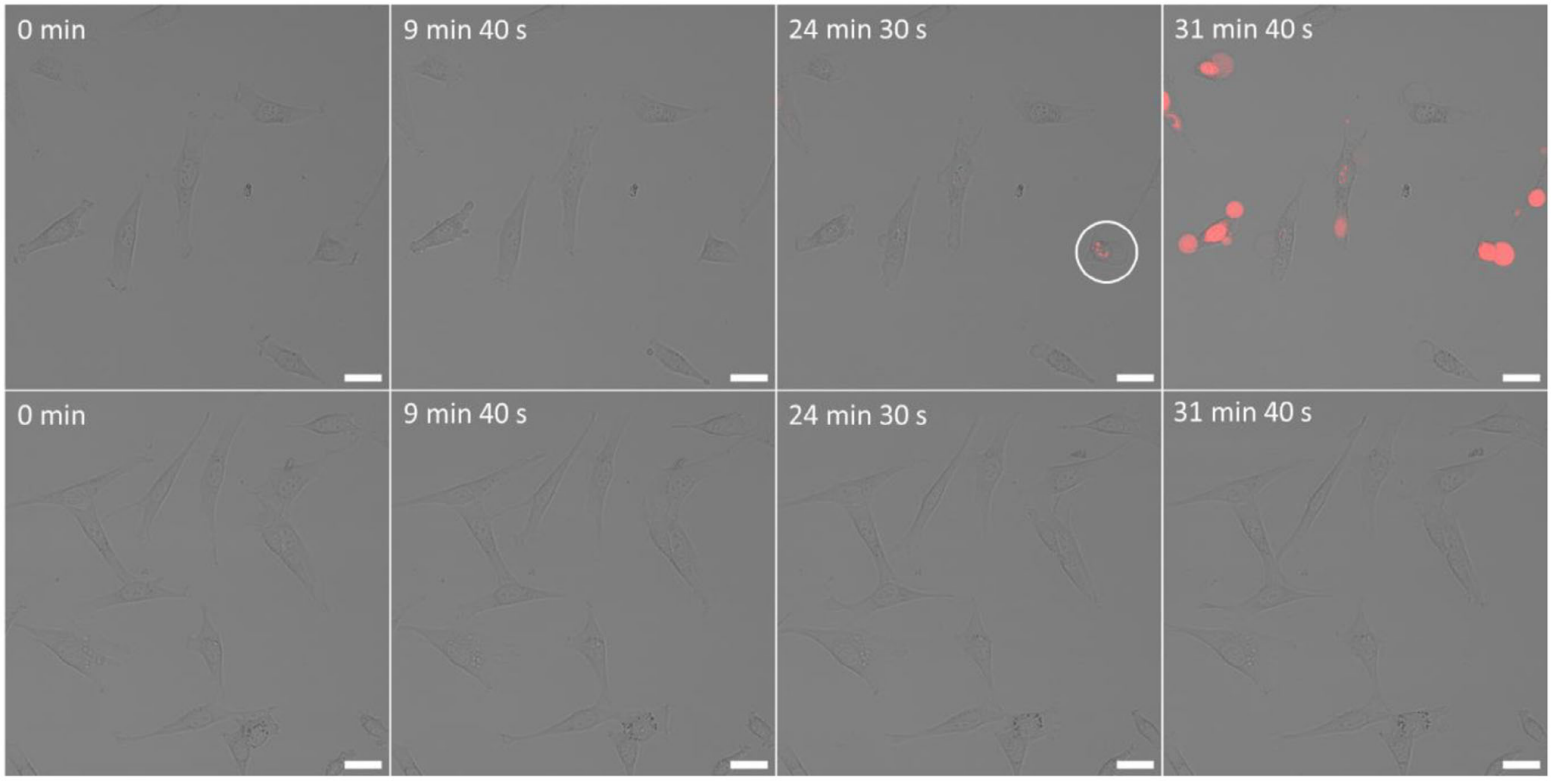
Microscopic observation of cell death caused by excitation at 488 nm (12 mW) (exposure times are shown for each image). Top row: Cells were incubated with 5 um of **COU‐BDP‐BF** for 2 h prior to the experiment. Bottom row: Cells were incubated with phenyl red‐free media for 2 h, 0.1 µg mL^−1^ of PI was added immediately before imaging both rows. Images are an RGB merge of the PI fluorescence (λ_ex_ = 543 nm, 30 mW; λ_emi_ = 620–700 nm) channel, and the transmission channel. Circles highlight the regions where cell death is first observed. Scale bars = 10 µm.

To evaluate the photocatalytic activity of **COU‐BDP‐BF**, cells were grown in chamber slides with 2 wells; in one of the wells cells were dosed with a 5 µM **COU‐BDP‐BF** solution, and at the same time, the media in the other well contained no **COU‐BDP‐BF** to be used as a control. Propidium Iodide (PI), a cell‐impermeant fluorescent dye, was added to both at a concentration of 0.1 µg mL^−1^ to assess cellular viability. PI emission increases 20‐ to 30‐fold when it intercalates between bases in double stranded DNA. However, it is excluded from live cells with intact plasma membranes, and it is shown to be non‐toxic to the **COU‐BDP‐BF** free cells. Hence, it only becomes visible in the experiment after cell death when the cell membrane becomes permeable and intercalation with DNA occurs. It is a protocol that can distinguish between necrotic, necroptotic, or ferroptotic, and apoptotic cells. Additionally, necrotic cells display a swollen, decondensed morphology that is very distinct from the features exhibited by apoptotic nuclei.^[^ ^62^ ^]^ Both chambers of the slide were kept in the dark until the start of the experiment where they were illuminated with a 488 nm laser (12 mW) at 1000 Hz/line scan speed (1‐line average, bidirectional scanning). 543 nm laser excitation was also used to capture PI emission (used to follow the onset of necrosis) and a transmission image in parallel.^[^ ^63^ ^]^ Figure 6 demonstrates that it takes 24 min and 30 s for the first cell treated with **COU‐BDP‐BF** to show confirmation of necrosis (red PI emission, circled) and 31 min and 40 s for widespread cell death when continuously exposed to 488 nm argon laser light. Additionally, cells dosed with **COU‐BDP‐BF** show blebbing and alteration to their overall morphology on the transmission images after under 10 min. This is in contrast to the untreated NIH‐3T3 cells which showed no signs of necrosis (both on transmission images and PI fluorescence channel) at the same time scales and further. Although membrane blebbing also occurs during apoptosis, the cell swelling and absence of apoptotic bodies support our assessment of **COU‐BDP‐BF** triggering necrosis (Figure S11, Supporting Information). We stopped monitoring the cells with no added **COU‐BDP‐BF** after 40 min, when the untreated cells were still viable. This indicates that **COU‐BDP‐BF** accelerates necrosis onset when compared to the control sample.

Cytotoxicity assays were carried out to ensure that **COU‐BDP‐BF** is not toxic to the cells in the absence of light activation. Cytotoxicity data for **COU‐BDP‐BF** were collected after incubation at concentrations ranging from 0 to 10 µm at times of 1, 6 and 24 h. It was found that even at concentrations of 10 µm and incubation times of 24 h cells remain viable and there is no difference in cell variability between the treated and untreated cells (Figure S12, Supporting Information).

**Figure 7:**
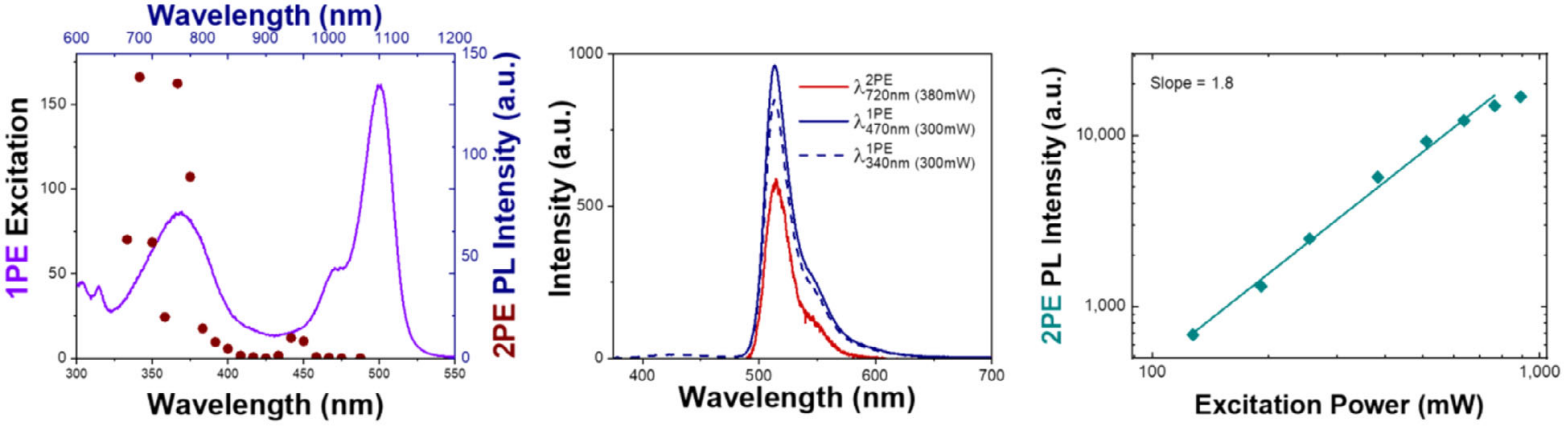
**COU‐BDP‐BF** in ethyl acetate. a) One‐photon absorption (solid purple line) and two‐photon excitation (λ_emi_ = 560 nm, brown dots) spectra. b) One‐ (λ_ex_ = 340 nm, dashed navy line and λ_ex_ = 470 nm, solid navy line) and two‐photon induced (λ_ex_ = 720 nm, red line) emission spectra of **COU‐BDP‐BF**. c) Excitation power dependency of the 2PE‐induced photoluminescence intensity, slope 1.8 ± 0.1, σ^2^ = 30 ± 3 GM.

Finally, the two‐photon absorption (2PA) properties of **COU‐BDP‐BF** and **ref‐BDP‐BF** were investigated. According to our results, **ref‐BDP‐BF** has negligible 2PA activity. In contrast, 2PA (650‐800 nm) of **COU‐BDP‐BF** leads to measurable fluorescence from BODIPY (Figure 7). 2P activity was confirmed by the quadratic dependence of the recorded maximum‐emission intensity on the excitation power. The obtained σ^2^ value was 30 ± 3 GM in ethyl acetate, comparable with other 2‐photon active coumarin systems.^[^ ^26^ ^]^ These results highlight that FRET probes based on BODIPY can be excited with physiologically safer NIR. Nevertheless, more studies are required to increase the cross‐section of the antenna for PDT applications.

## 3 Conclusion

In conclusion, we have developed a novel approach for designing biocompatible, heavy‐atom‐free BODIPY photosensitizers. Obtained results confirm the proposed photosensitization of BODIPY systems with organic cyclic modification on boron atom. Hence, the introduction of functional groups such as coumarin at the easily accessible *meso* position enables targeted localization of the BODIPY PS within specific organelles (e.g., the ER), enhancing therapeutic efficacy while reducing the required dose. In addition, the resulting compound exhibited effective (>97%) energy transfer between the coumarin antenna and PS, allowing for a larger Stokes’ shift, beneficial for bioimaging applications. We have shown that the PS is selectively accumulated in the ER and generates ROS upon light activation, leading to cell stress and ultimately death by necrosis. Future work will focus on extending the proposed approach for the construction of heavy‐atom free organelle‐targeting BODIPY PSs toward red‐ and NIR‐absorbing systems, particularly for efficient multiphoton excitation with NIR light.

## 4 Experimental Section

### General comment

All used reagents were purchased from Merck, Acros and Apollo Scientific. Dichloromethane (DCM), *N,N*‐dimethylformamide (DMF), and tetrahydrofuran (THF) were purified using MBraun SPS and stored over 4 Å molecular sieves. Starting materials including 2,4‐dimethylpyrrole, *p*‐hydroxybenzaldehyde, imidazole, diethyl 1,3‐acetonedicarboxylate, *p*‐toluenesulfonic acid, 2,3‐dichloro‐5,6‐dicyano‐1,4‐benzoquinone (DDQ), diisopropyl azodicarboxylate (DIAD), 3‐(dimethyloamino)phenol, *N,N*‐diisopropylethylamine (NEt(*i*‐Pr)_2_), trimethylamine, triphenylphosfine, *tert*‐butyldiphenylsilyl chloride (DPTBSCl), tetrabutylammonium fluoride (TBAF), acetyl chloride (AcCl), inorganic salts and solvents were used as received without additional purification. Synthesis of 9‐chloroborafulorene was conducted as in our previously published work.^[^ ^42^ ^]^ Reactions and manipulations involving air and moisture‐sensitive reagents were carried out under an argon atmosphere.

^1^H, ^11^B, and ^13^C NMR spectra were recorded on Agilent NMR 400 MHz DDR2, JOEL JNM‐ECZL 600 MHz, and Bruker Advance III 300 MHz spectrometers. ^1^H‐^1^H COSY (600 MHz frequencies) and ^1^H‐^13^C HSQC (600 MHz, 150 MHz) NMR spectra were recorded on JOEL JNM‐ECZL 600 MHz spectrometer. ^1^H and ^13^C chemical shifts were referenced to TMS using known chemical shifts of solvent residual peaks. ^11^B NMR chemical shifts are given relative to BF_3_·Et_2_O. HR‐MS analyses were performed on a GCT Premier mass spectrometer equipped with an EI ion source and a Maldi SYNAPT G2‐S HDMS spectrometer equipped with an Supporting Information ion source.

EPR spectroscopy were conducted using a JEOL JES‐FA 200, X‐band CW‐EPR spectrometer operating at 100 kHz field modulation. Measurement were carried out using the microwave power equal to 0.995 mW and modulation width equal to 0.1 and 0.01 mT. The g‐factor value was determined using the JEOL internal manganese (Mn) standard.

### Synthesis

10′‐(4‐((*tert*‐butyldiphenylsilyl)oxy)phenyl)‐1′,3′,7′,9′‐tetramethyl‐4′λ^4^,5λ^4^‐spiro[dibenzo[*b*,*d*]borole‐5,5′‐dipyrrolo[1,2‐*c*:2′,1′‐*f*][1,3,2]diazaborinine] (**2**):

Dimethylpyrrole (3.87 g, 7.29 mmol) was dissolved in dry DCM (50 mL), then NEt(*i*‐Pr)_2_ (1.35 mL, 7.29 mmol, 1 eqv.) was added. The resulting solution was moved to the Schlenk vessel with 9‐chloroborafluorene (1.57 g, 7.29 mmol, 1 eqv.) which was earlier dissolved in DCM (50 mL). The reaction mixture was left stirring overnight, then extracted with H_2_O and DCM (3×50 mL). Combined organic phase was dried over anhydrous MgSO_4_, filtered, and concentrated in vacuo. The crude product was further purified by column chromatography (SiO_2_, PhMe/hexane 3:2 as eluent) to give orange solid (2.18 g, 43.2% yield) after concentration. ^1^H NMR (400 MHz, CDCl_3_) δ = 7.77 (d, *J* = 7.2 Hz, 4H), 7.63 (d, *J* = 7.6 Hz, 2H), 7.49 – 7.37 (m, 7H), 7.26 – 7.19 (m, 3H), 7.18 – 7.05 (m, 4H), 6.96 (d, *J* = 8.0 Hz, 2H), 5.80 (s, 2H), 1.48 (s, 6H), 1.40 (s, 6H), 1.19 (s, 9H) ppm. ^13^C{^1^H} NMR (101 MHz, CDCl_3_) δ = 156.22, 153.99, 150.38, 142.26, 140.01, 135.65, 135.19, 132.54, 131.60, 130.16, 130.07, 129.38, 127.84, 127.24, 127.09, 121.59, 120.85, 118.72, 26.58, 26.15, 19.54, 15.00, 14.67 ppm. ^11^B NMR (96 MHz, CDCl_3_) δ = ‐0.38 ppm. HRMS (ESI, positive ion mode), calculated for C_47_H_45_BN_2_OSi^+^ [M^+^]: 692.33887; found: 692.33822.

4‐(1′,3′,7′,9′‐tetramethyl‐4′λ^4^,5λ^4^‐spiro[dibenzo[*b*,*d*]borole‐5,5′‐dipyrrolo[1,2‐c:2′,1′‐*f*][1,3,2]diazaborinin]‐10′‐yl)phenol (**3**):

Compound **2** (2.23 g, 3.21 mmol) was dissolved in dry THF (100 mL) and cooled down to –5 °C. Then, TBAF (1.29 g, 4.02 mmol, 1.25 eqv.) was added, and the reaction mixture became dark. After stirring for 2.5 h the mixture was poured into a cold saturated aqueous solution of NH_4_Cl (60 mL), then the reaction mixture was extracted with Et_2_O (3×50 mL). The combined organic phase was dried over anhydrous MgSO_4_, filtered and concentrated in vacuo. The crude product was washed with hexane to give orange solid (1.31 g, 89.8% yield) after filtration. ^1^H NMR (400 MHz, CDCl_3_) δ = 7.63 (d, *J* = 7.5 Hz, 2H), 7.33 – 7.27 (m, 2H), 7.26 – 7.22 (m, 4H), 7.11 (t, *J* = 7.2 Hz, 2H), 7.02 (d, *J* = 7.4 Hz, 2H), 5.83 (s, 2H), 5.18 (s, 1H), 1.49 (s, 12H) ppm. ^13^C{^1^H} NMR (101 MHz, CDCl_3_) δ = 156.21, 154.09, 150.38, 142.13, 140.00, 131.72, 130.15, 129.78, 128.22, 127.24, 127.08, 121.63, 118.71, 116.01, 14.95, 14.66 ppm. ^11^B NMR (96 MHz, CDCl_3_) δ = –0.31 ppm. HRMS (ESI, positive ion mode), calculated for C_31_H_27_BN_2_O [M^+^]: 454.22110; found: 464.22108.

4‐(1′,3′,7′,9′‐tetramethyl‐4′λ^4^,5λ^4^‐spiro[dibenzo[*b*,*d*]borole‐5,5′‐dipyrrolo[1,2‐c:2′,1′‐*f*][1,3,2]diazaborinin]‐10′‐yl)phenyl 2‐(7‐(dimethylamino)‐2‐oxo‐2H‐chromen‐4‐yl)acetate (**COU‐BDP‐BF**):

Triphenylphosphine (315 mg, 1.2 mmol, 1.2 eqv) was dissolved in dry THF (20 mL) and cooled to ‐3 °C. Simultaneously, DIAD (0.25 mL, 1.2 mmol, 1.2 eqv.) was added to another flask with dry THF (10 mL). Then, the DIAD solution was transferred to triphenylphosphine. The temperature rose to 1 °C. Compound **1** (263 mg, 0.99 mmol, 1 eqv.) and **3** (450 mg, 0.99 mmol) were added, and the reaction mixture was stirred at RT overnight. Then, the reaction mixture was extracted with DCM (3×50 mL). The combined organic layer was dried over anhydrous MgSO_4_, filtered, and concentrated in vacuo. The crude product was further purified by column chromatography (SiO_2_, 20% AcOEt/hexane as eluent) to give orange solid (230 mg, 28.0% yield) after concetration. ^1^H NMR (600 MHz, CDCl_3_) δ = 7.62 (d, *J* = 7.5 Hz, 2H, BF), 7.54 (d, *J* = 9.0 Hz, 1H, COU), 7.47 – 7.44 (m, 2H, BDP‐Ph), 7.28 – 7.22 (m, 6H, BDP‐Ph + BF), 7.10 (td, *J* = 7.1, 0.9 Hz, 2H, BF), 6.70 (dd, *J* = 9.0, 2.6 Hz, 1H, COU), 6.58 (d, *J* = 2.6 Hz, 1H, COU), 6.21 (s, 1H, COU), 5.82 (d, *J* = 1.0 Hz, 2H, BPD‐pyrrole), 3.97 (s, 2H, COU‐CH_2_), 3.09 (s, 6H, COU‐NMe_2_), 1.49 (s, 6H, BDP‐Me), 1.44 (s, 6H, BDP‐Me) ppm. ^13^C{^1^H} NMR (101 MHz, CDCl_3_) δ = 167.46, 161.63, 156.10, 154.54, 153.10, 150.76, 150.39, 147.57, 140.74, 139.87, 134.01, 131.23, 130.12, 129.74, 127.30, 127.11, 125.14, 122.12, 121.94, 118.74, 111.08, 109.17, 108.27, 98.46, 40.16, 38.27, 14.95, 14.67 ppm. ^11^B NMR (96 MHz, CDCl_3_) δ = –0.20 ppm. HRMS (ESI, positive ion mode), calculated for C_44_H_38_BN_3_O_4_^+^ [MH^+^]: 684.30281; found: 684.30272.

A detailed synthetic protocols for the remaining compounds are provided in the Supporting Information.

### Crystallographic studies

Single crystals suitable for X‐ray diffraction measurements of **ref‐COU**, **2,** **3**, **ref‐BDP‐BF**, and **COU‐BDP‐BF** were obtained by slow evaporation of DCM or AcOEt solutions. The crystallizations were performed in open vials at room temperature. X‐ray diffraction data were collected on a SuperNova diffractometer equipped with an Atlas detector using Cu‐K*α* radiation (*λ* = 1.54184 Å). Data for all samples were collected at 100 K, using the Oxford cryosystem temperature device. Data reduction and analysis were carried out with the CrysAlisPro program. All structures were solved by intrinsic phasing using SHELXT^[^ ^64^ ^]^ and refined using SHELXL‐2014^[^ ^65^ ^]^ with Olex2 suite.^[^ ^66^ ^]^ All non‐hydrogen atoms were refined anisotropically. Selected crystal data are summarized in Table S1. Crystallographic Information Files (CIFs) have been deposited with the Cambridge Crystallographic Data Centre as supplementary publications no. 2425189 (**ref‐COU**), 2425190 (**2**), 2425186 (**3**), 2425188 (**ref‐BDP‐BF**), and 2425187 (**COU‐BDP‐BF**).

### Theoretical computations

Single‐molecule calculations for **ref‐COU**, **ref‐BDP‐BF**, and **COU‐BDP‐BF** were performed using *Gaussian16* program.^[^ ^67^ ^]^ In the first step the molecules were optimized in their ground states using PBE1PBE (DFT)^[^ ^68^ ^]^ method with 6–311++G(d,p) basis set.^[^ ^69^ ^]^ Starting geometries were adopted from crystal structures. After geometry optimization, the vibrational frequencies were calculated, and the results showed that the optimized structures are stable geometric structures (no imaginary frequencies). In the next step, excited state geometries were obtained with TD‐DFT methods (PBE1PBE /6–311++G(d,p)). The absorption spectra were calculated at ground state geometry, while for emission properties, the geometry was fully optimized. Natural transition orbitals (NTO) were calculated for each excited state. The molecular orbitals and natural transition orbitals were visualized with the *Avogadro* software.^[^ ^70^ ^]^

### Optical properties and photocatalytic activity

Absorption spectra were recorded using a Hitachi U‐2800 spectrophotometer. Emission and fluorescence quantum yields were recorded using spectrofluorometer Edinburgh Instruments FS5. The measurements were performed at room temperature, according to published procedures.^[^ ^71^, ^72^ ^]^ Suprasil quartz cuvettes (10.00 mm) were used. Fluorescence quantum yields for solutions were determined using reference substance (for BODIPY compounds fluorescein in 0.1 m NaOH Φ^F^_ref_ = 0.95,^[^ ^73^ ^]^ for **ref‐COU** quinine sulfate in 0.1 m H_2_SO_4_ Φ^F^_ref_ = 0.577^[^ ^73^ ^]^ using the following formula:

$$\phi^{F}=\phi_{ref}^{F}\cdot \frac{F_{x}}{F_{ref}}\cdot \frac{1-10^{-A_{ref}}}{1-10^{-A_{x}}}\cdot \frac{n_{x}^{2}}{n_{ref}^{2}}$$

where:

*F* is the emission integral over the emissive range of sample (x) and reference (ref), *A* is the absorbance at the excitation wavelength, *n* is the refractive index of the used solvents. All measurements were carried out at room temperature.

Fluorescence lifetime measurements were acquired using Time Correlated Single Photon Counting (TCSPC) system equipped with a picosecond pulsed 340 nm EPLED source. The singlet oxygen emission was measured using a thermoelectric cooled NIR‐PMP unit (Hamamatsy, wavelength range 950–1650 nm) integrated with FS5 spectrofluorometer.

Multiphoton spectroscopy was carried out using a tunable femtosecond pulsed laser (680 – 1300 nm, Coherent Discovery TPC, 100 fs, 80 MHz) and an Ocean Optics HR2000Pro (2048‐pixel linear CCD Sony ILX5 chip, 200 µm slit, H3 grating, 350 – 850 nm spectral region) spectrometer. The spectrometer has also been equipped with a perpendicularly mounted 365 nm LED (nichia, 1 W) and been operated using a time resolved detection and accumulation algorithm written in Labview2013 program. Cross‐section determination was using methods and reference standards as reported throughout.^[^ ^57^ ^]^

TPA σ^2^ was determined via the following expression:

$$\sigma^{2}=\frac{F_{s}}{\phi_{s}c_{s}}\times \frac{\phi_{R}c_{R}\sigma_{R}^{2}}{F_{R}}$$

*F* is the emission integral over the entire emissive range, Φ is the quantum yield as previously, and *c* is the molar sample concentration.

Quantum yield of photogeneration of singlet oxygen was investigated indirectly with UV–Vis spectroscopy and a specific trap of ^1^O_2_ – 2,3,4,5‐tetraphenylcyclopentadienone (TPCPD, used for excitation at 375 nm) and diphenylisobenzofuran (DPBF, used for excitation at 505 nm). The DCM solution containing a singlet oxygen trap and an investigated compound (at concentration of 0.02 mm) was constantly illuminated with Oxxius diode laser: 375 nm (LBX‐375–70‐CSB‐PPA) or 505 nm (LBX‐505–70‐CSB‐PPA) operating at 20 and 10 mW, respectively. UV–Vis spectra of the solution were collected in even time periods with AvaSpec‐ULS2048XL‐EVO‐RS‐UA spectrometer. A drop in the absorbance of TPCPD at 510 nm (excitation at 375 nm) and DPBF at 410 nm (excitation at 505 nm) in time was observed, indicating photogeneration of singlet oxygen. The quantum yield of singlet oxygen photogeneration was determined with respect to phenalenone (PN) for laser 375 nm (DCM), methylene blue (MB) for laser 505 nm (DCM and DMF) or 2,6‐diiodoBODIPY for laser 505 nm (toluene) by the relative method with the following equation:

$$\phi \Delta_{x}=\phi \Delta_{r}\frac{k_{x}1-10^{-A_{r}}}{k_{r}1-10^{-A_{x}}}$$

where: x and r stands for substance under study and reference (PN, MB or 2,6‐diiodoBODIPY), respectively; Φ_Δ_ is singlet oxygen quantum yield (for PN in DCM: 0.95,^[^ ^74^ ^]^ for MB in DCM: 0.57,^[^ ^75^ ^]^ for MB in DMF: 0.49,^[^ ^76^ ^]^ for 2,6‐diiodoBODIPY in toluene: 0.85,^[^ ^77^ ^]^ *k* denotes to TPCPD or DPBF consumption rates, *A* represents the absorbance of the investigated compound or reference at 375 or 505 nm.

A detailed protocol for the photooxidation of furoic acid is provided in the Supporting Information.

### Microscopy

LSCM experiments were conducted using NIH 3T3 embryonic mouse skin fibroblast cell line, sourced from ATCC (CRL‐1658), and were established and maintained in a category 2 cell culture facility. Cells were maintained in exponential growth as monolayers in F‐12/DMEM (Dulbecco's Modified Eagle Medium) 1:1 that was supplemented with 10% foetal bovine serum (FBS). Cells were grown in 75 cm^2^ plastic culture flasks, with no prior surface treatment. Cultures were incubated at 37 °C, 10% average humidity, and 5% (v/v) CO_2_. Cells were harvested by treatment with 0.25% (v/v) trypsin solution for 5 min at 37 °C. Dosing of cells with dyes was as reported throughout. Acquisition of microscopy images used a commercial LSCM (SP5 II, Leica Microsystems) microscope with excitation provided by a fibre coupled 80 mW variable power 355 nm Nd:YAG CW laser. The intensity of emission was quantified by fibre‐ 74 coupled (200 micron) high performance matched tandem avalanche photodiodes (Leica ADPs, Becker & Hickl ID‐120).

Dosing of cells was from 1 mm stock solutions of the dyes in DMSO which were diluted appropriately to concentration of 5 µm for one‐photon excitation. Dilutions to make the dosing solutions were into F‐12/DMEM (1:1) lacking phenol red and maintaining the DMSO concentration to < 1% v/v.

Microscopy was conducted using ibidi well slides. Cells were grown under standard conditions in the channels to ≈80% confluency before dosing with the dyes and incubation. Prior to imaging, the dye‐containing media was removed and replaced with media lacking phenol red.

### Cytotoxicity studies

Cytotoxicity of the **COU‐BDP‐BF** dye was determined via an Chemometec Via1‐Cassette assay containing the fluorophores acridine orange (AO) and 4′,6‐diamidino‐2‐phenylindole (DAPI), which are immobilized within the cassette's channels. NIH‐3T3 cells were grown in 24‐well plates at 37 °C and 5% CO_2_ to circa 90% confluency in 500 µL media. The media was removed, and cells were dosed with 500 µL of the selected dye as a solution in media without phenol red at the desired concentration. Incubation for the required time was followed by removal of the dye, rinse with 400 µL phosphate buffer solution (PBS), and addition of 400 µL of 5% Trypsin in PBS. After 3 min, 1 mL live cell was added to inhibit the activity of Trypsin, and the solution was agitated with the pipette to suspend the cells. The solution was placed in a cryovial, shaken, and a Via1‐cassette withdrew 60 µL of cell suspension. The cassette was placed in a NucleoCounter NC‐3000 cell counter to assess the % viability. 3 replicates for each set of conditions were made.

## Supporting Information

The authors have cited additional references within the Supporting Information.^[^ ^30^, ^31^ ^]^

## Conflict of Interest

The authors declare no conflict of interest.

## Supporting information



Supporting Information

Supporting Information

## Data Availability

The data supporting this article have been included as part of Supporting Information.
