# Supplementary material for: Heavy‐Atom Free Bodipy‐Borafluorene Photosensitizer Decorated with Coumarin Antenna Selectively Staining Endoplasmic Reticulum for Application in PDT
Source: Chemistry. 2025 Jul 23;31(45):e01949. doi: 10.1002/chem.202501949 (PMC12351428; doi:10.1002/chem.202501949)
Supplement: Supplementary file 1 — Supporting Information [file CHEM-31-e01949-s002.docx]

**SUPPORTING INFORMATION**

**for**

**Heavy-atom free BODIPY-borafluorene photosensitizer decorated with coumarin antenna selectively staining endoplasmic reticulum for application in PDT**

Karolina Wrochna,^a^ Dawid R. Natkowski,^a^ Agata Blacha-Grzechnik,^b,c^ Sandra Pluczyk-Malek,^b,c^ Connor B. Armstrong,^d^ Paolo J. Mastroeni,^d^ Dominic J. Black,^d^ Robert Pal,^d^ Krzysztof Durka,^a^ Paulina H. Marek-Urban,^a^* Patrycja Stachelek ^d^*

^a^ *Faculty of Chemistry, Warsaw University of Technology, Noakowskiego 3, 00-664 Warsaw, Poland*

^b^ *Faculty of Chemistry, Silesian University of Technology, Strzody 9, 44-100 Gliwice, Poland.*

^c^ *Centre for Organic and Nanohybrid Electronics, Silesian University of Technology, Konarskiego 22B, 44-100 Gliwice, Poland*

^d^ *Department of Chemistry, Durham University, South Road, Durham, UK*

**Table of contents**

[Synthesis 2](#_Toc196205007)

[Crystallographic studies 3](#_Toc196205008)

[Theoretical computations 5](#_Toc196205009)

[Optical properties 15](#_Toc196205010)

[Photocatalytic activity and photostability studies 16](#_Toc196205011)

[Microscopy 19](#_Toc196205012)

[Cytotoxicity Studies 20](#_Toc196205013)

[NMR spectra and HRMS data 20](#_Toc196205014)

[References for Supporting Information 32](#_Toc196205015)

# Synthesis

*Ethyl 2-(7-(dimethylamino)-2-oxo-2H-chromen-4-yl)acetate* (**ref-COU**)
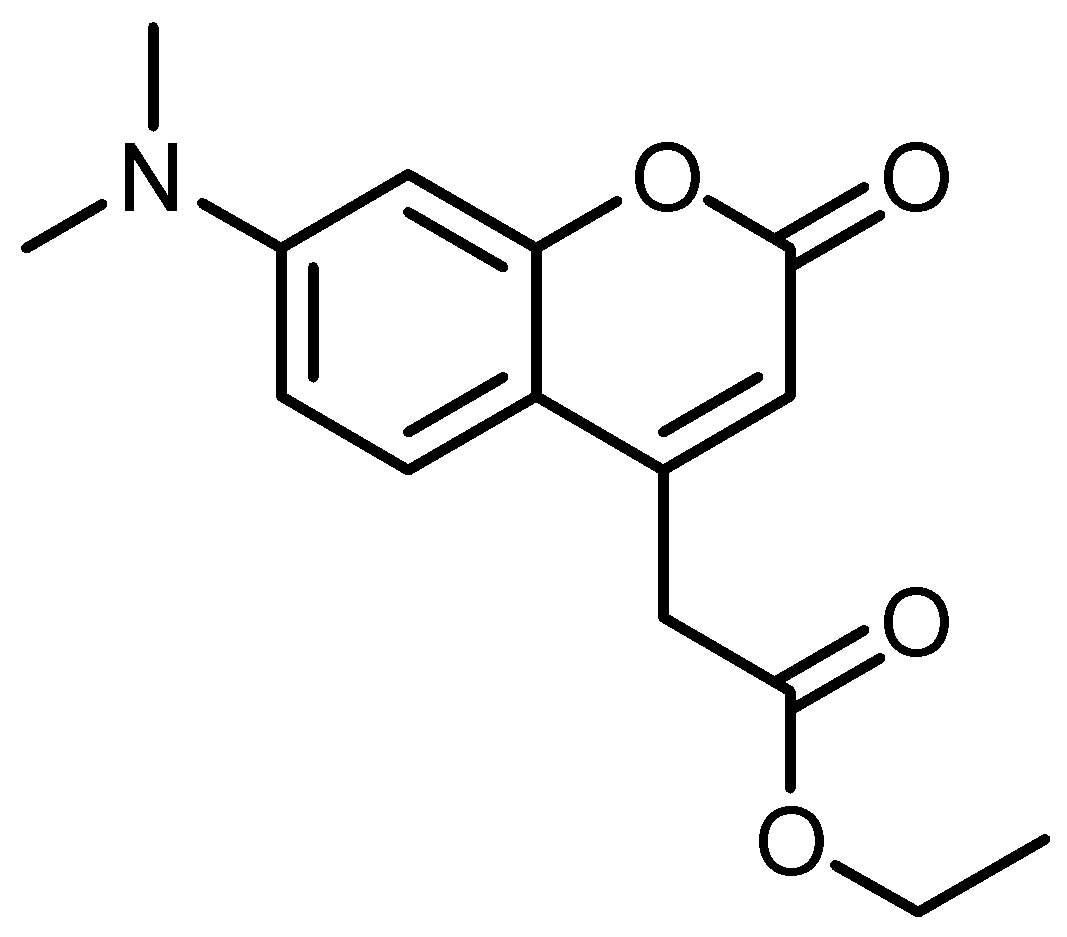


Synthesis was performed following literature procedure.^1^ 3-(dimethyloamino)phenol (4.29 g, 31 mmol) was placed in 3-neck round-bottom flask which was further purged with argon. The solid was dissolved in EtOH (15 mL), then diethyl 1,3-acetonedicarboxylate (6.25 mL, 34.0 mmol, 1.1 eqv.) and ZnCl_2_ (5.22 g, 37.5 mmol, 1.2 eqv.) were added and the reaction mixture was heated in reflux for c.a. 20 h. Then, the resulting suspension was diluted with DCM (40 mL) and was poured into ice cold water (c.a. 200 mL) to give red solution with brown oil at the bottom. The reaction mixture was extracted with DCM (3x50 mL) and the combined organic phase was dried over anhydrous MgSO_4_, filtered and concentrated to give dark purple oil which was diluted with EtOH and left in the fridge overnight to precipitate yellow soild. The precipitate was filtered, washed with EtOH and purified by crystallization from EtOH (60 mL) to give yellow crystalline solid in two fractions (A: 3.53 g, B: 0.46 g, ∑ 3.99 g, 46.8% yield). ^1^H NMR spectra in accordance with literature.^2^ ^1^H NMR (400 MHz, CDCl_3_) δ = 7.40 (d, *J* = 9.0 Hz, 1H), 6.61 (dd, *J* = 9.0, 2.6 Hz, 1H), 6.51 (d, *J* = 2.6 Hz, 1H), 6.05 (d, *J* = 0.9 Hz, 1H), 4.17 (q, *J* = 7.1 Hz, 2H), 3.67 (d, *J* = 0.8 Hz, 2H), 3.05 (s, 6H), 1.24 (t, *J* = 7.1 Hz, 3H) ppm. ^13^C{^1^H} NMR (151 MHz, CDCl_3_) δ = 169.18, 161.81, 156.05, 153.05, 148.50, 125.37, 110.85, 109.06, 108.61, 98.46, 61.66, 40.17, 38.35, 14.17 ppm. HRMS (ESI, positive ion mode), calculated for C_15_H_17_NO_4_^+^ [MH^+^]: 276.12303; found: 276.12293.

*2-(7-(dimethylamino)-2-oxo-2H-chromen-4-yl)acetic acid* (**1**)
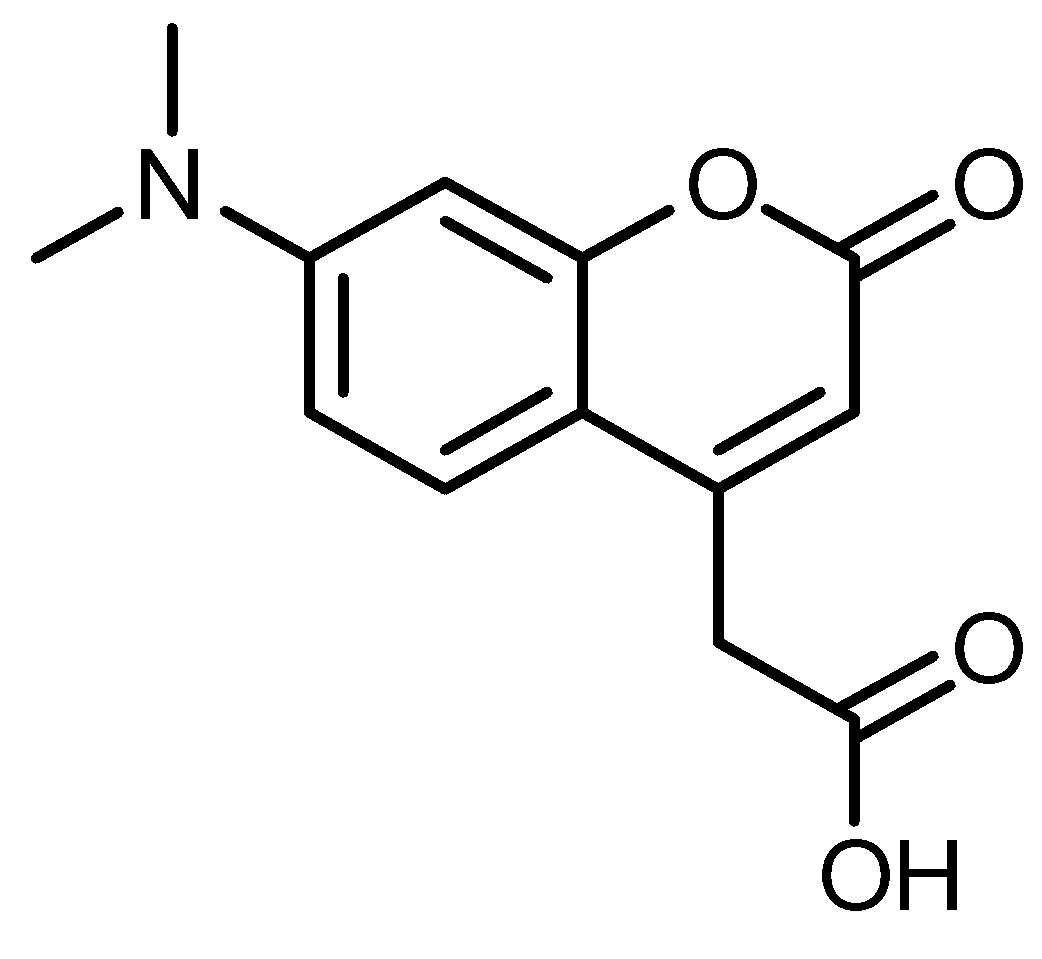


Synthesis was performed following literature procedure.^2^ **ref-COU** (3.53 g, 12.8 mmol) was dissolved in THF (100 mL), then the solution
of LiOH ∙ H_2_O (1.07 g, 25.6 mmol, 2 eqv.) in H_2_O (60 mL) was added and the solution changed color from pink to orange. After c.a. 3 h of stirring the reaction mixture was diluted with H_2_O (100 mL) and was extracted with Et_2_O (3x150 mL).
The aqueous phase was acidified to pH ≈ 1 using 0.1M HCl to precipitate yellow solid which was further filtered and washed with MeCN (3x60 mL). The product was obtained as yellow needles (2.66 g 84.0% yield) after filtration. ^1^H NMR spectra in accordance with literature.^3^ ^1^H NMR (400 MHz, DMSO-D6) δ = 7.43 (d, *J* = 8.9 Hz, 1H), 6.70 (d, *J* = 8.9 Hz, 1H), 6.52 (s, 1H), 6.02 (s, 1H), 3.75 (s, 2H), 3.35 (s, 1H), 2.98 (s, 6H) ppm. ^13^C{^1^H} NMR (151 MHz, DMSO-*d*6) δ = 171.33, 161.22, 155.97, 153.38, 150.77, 126.58, 126.51, 110.15, 109.67, 108.65, 98.04, 37.79 ppm. HRMS (ESI, positive ion mode), calculated for C_13_H_13_NO_4_^+^ [MH^+^]: 248.09173; found: 248.09153.

*4-(tert-butyldiphenylsilyloxy)benzaldehyde*

Synthesis was performed following literature procedure.^3^ 4-hydroxybenzaldehyde (8.74 g, 71.6 mmol) and imidazole (5.36 g, 78.7 mmol, 1.1 eqv.) were dissolved in dry DMF (150 mL). After 15 minutes of stirring DPTBSCl (20.45 mL, 78.7 mmol, 1.1 eqv.) was added and stirring was continued for 3 h. Then, the reaction mixture was poured into water (white suspension, warmed) and extracted with AcOEt (3x120 mL). The combined organic phase was washed with saturated aqueous solution
of LiCl (3x100 mL) and brine (100 mL), dried over anhydrous MgSO_4_, filtered and concentrated
in vacuo. The crude product was further purified by column chromatography (SiO_2_, CHCl_3_/hexane 1:1 as eluent) to give light brown oil (8.64 g, 33.5% yield) after concentration. ^1^H NMR spectra in accordance with literature.^4^ ^1^H NMR (400 MHz, CDCl_3_) δ = 9.81 (s, 1H), 7.73 – 7.62 (m, 6H), 7.53 – 7.34 (m, 6H), 6.87 (d, J = 8.6 Hz, 2H), 1.12 (s, 9H) ppm.
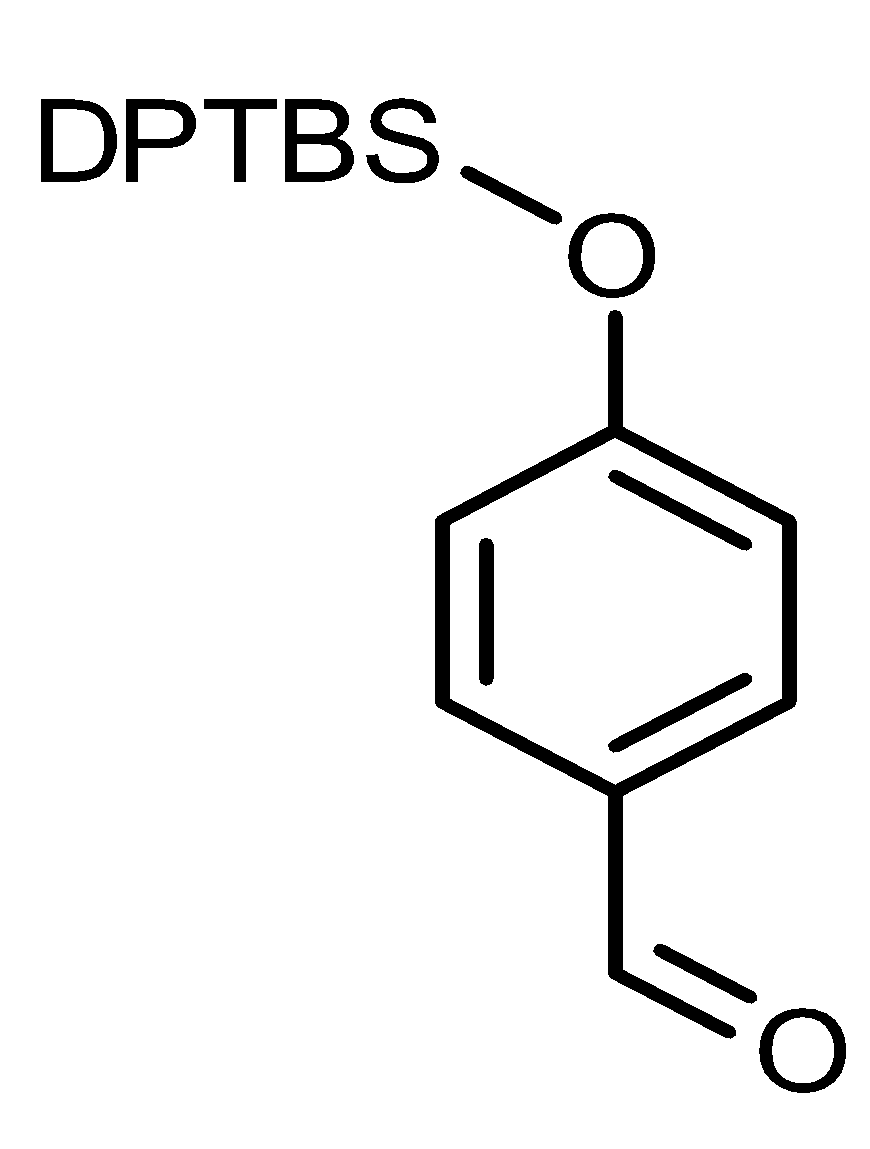


*(Z)-2-((4-((tert-butyldiphenylsilyl)oxy)phenyl)(3,5-dimethyl-2H-pyrrol-2-ylidene)methyl)-3,5-dimethyl-1H-pyrrole*

4-(*tert*-butyldiphenylsilyloxy)benzaldehyde (8.64 g, 24.0 mmol) was dissolved in dry DCM (120 mL), then 2,4-dimethylpyrrole (4.95 mL, 48.0 mmol, 2 eqv.) and
*p*-toluenesulfonic acid were added. The solution became dark red and was left stirring overnight. Then, DCM (50 mL) and DDQ (5.45 g, 24.0 mmol, 1 eqv.) were added. After 0.5 h of stirring the reaction was extracted with DCM (3x50mL). The combined organic phase was dried over anhydrous MgSO4, filtered and concentrated in vacuo to give dark solid (3.87 g, 30.4% yield). The crude product was used in the next step without further purification.
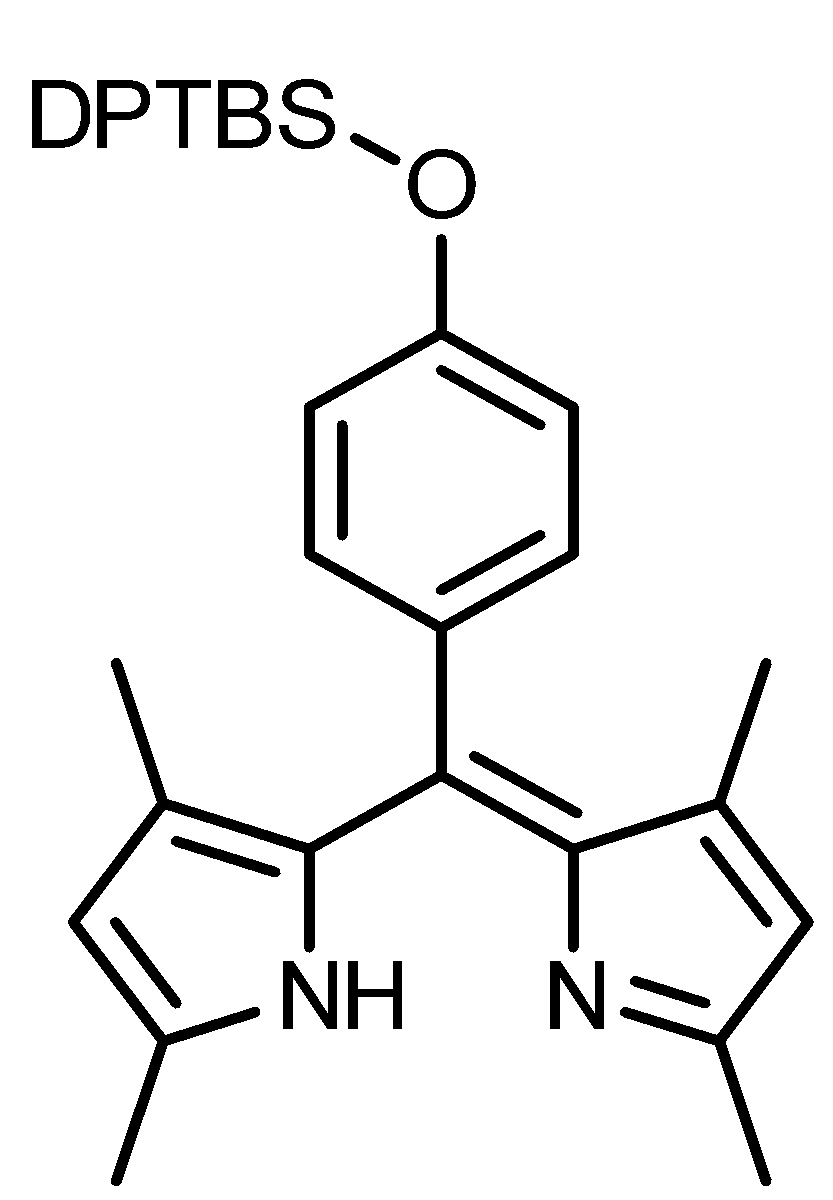


*4-(1',3',7',9'-tetramethyl-4'λ^4^,5λ^4^-spiro[dibenzo[b,d]borole-5,5'-dipyrrolo[1,2-c:2',1'-f][1,3,2]diazaborinin]-10'-yl)phenyl acetate* (**ref-BDP-BF**)

Compound **3** (144 mg, 0.32 mmol) was dissolved in dry DCM (10 mL). Then, NEt_3_ (0.05 mL, 0.35 mmol, 1.1 eqv.) and AcCl (0.02 mL, 0.33 mmol, 1.05 eqv.) were added and the reaction was stirred until the substrate consumption, which was monitored with TLC. Then, the reaction mixture was poured into water and extracted with CHCl_3_ (3x50 mL). The combined organic layer was diluted with water and KHCO_3_ was added until the pH ≈ 8, then extracted again with CHCl_3_ (3x50 mL), dried over anhydrous MgSO_4_, filtered and concentrated in vacuo. The crude product was further purified by filtration through silica (10% AcOEt/hexane as eluent) to give orange solid (108 mg, 68.0% yield). ^1^H NMR (400 MHz, CDCl_3_) δ = 7.63 (d, *J* = 7.6 Hz, 2H), 7.46 (d, *J* = 8.3 Hz, 2H), 7.35 – 7.29 (m, 3H), 7.28 – 7.22 (m, 3H), 7.11 (t, *J* = 7.1 Hz, 2H), 5.83 (s, 2H), 2.36 (s, 3H), 1.48 (d, *J* = 9.6 Hz, 12H) ppm. ^13^C{^1^H} NMR (101 MHz, CDCl_3_) δ = 169.03, 154.46, 151.10, 150.40, 139.95, 133.47, 131.32, 130.14, 129.59, 127.30, 127.11, 122.33, 121.90, 118.74, 21.25, 14.91, 14.68 ppm ^11^B NMR (96 MHz, CDCl_3_) δ = –0.32 ppm. HRMS (ESI, positive ion mode), calculated for C_33_H_29_BN_2_O_2_^+^ [MH^+^]: 497.23949; found: 497.23925
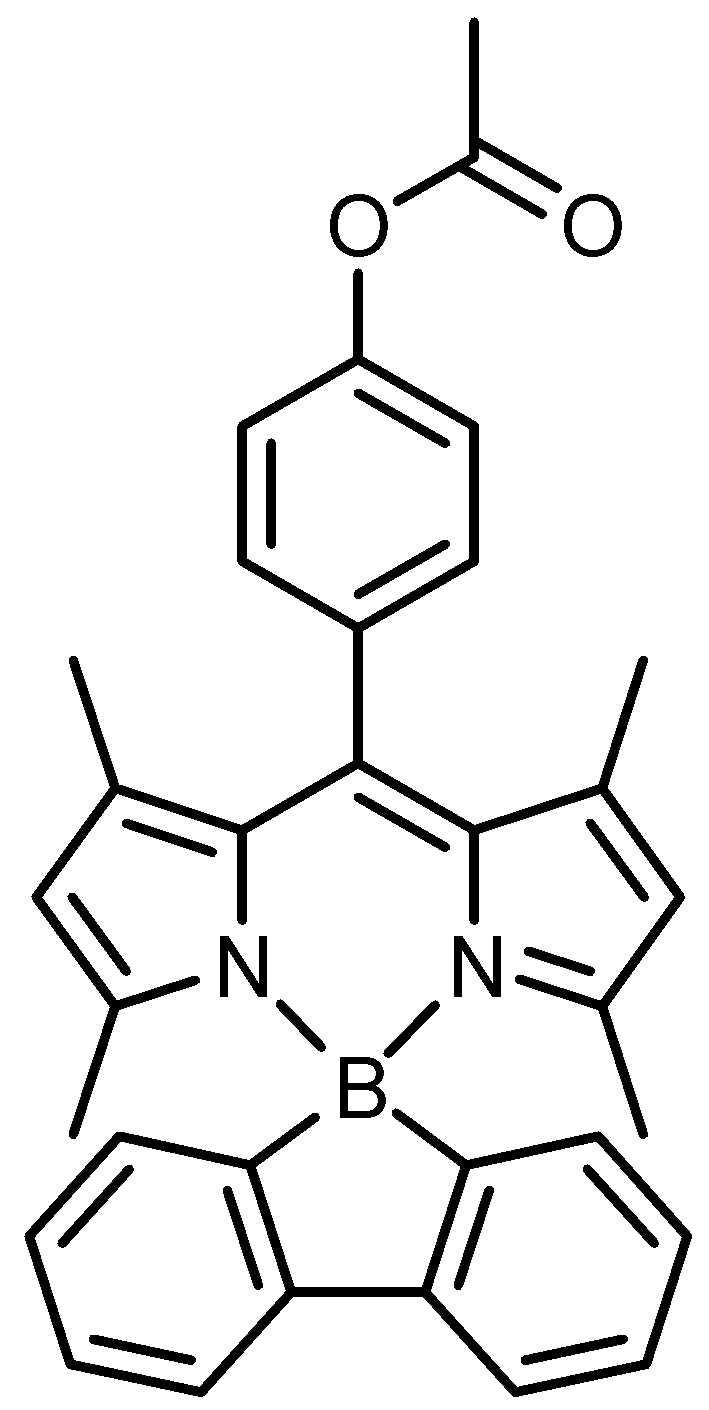


# Crystallographic studies


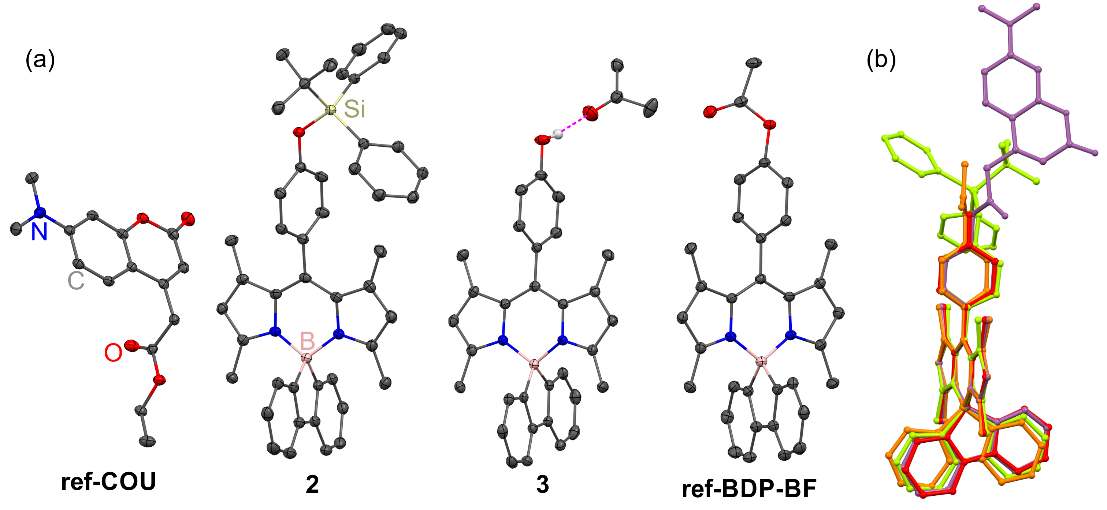


1. (a) ORTEP drawings of **ref-COU**, intermediate BODIPY complexes and **ref-BDP-BF**. Thermal ellipsoids were depicted on 50 % level of probability. Protons were omitted for clarity. (b) Overlay of the molecular structures of all BODIPY complexes: **3** (red), **2** (green), **ref-BDP-BF** (blue), **COU-BDP-BF** (plum).
2. Structural data for analysed crystal structures

| Identification code | **COU-BDP-BF** | **ref-BDP-BF** | **3** | **2** | **ref-COU** |
| --- | --- | --- | --- | --- | --- |
| Empirical formula | C_44_H_38_BN_3_O_4_ | C_33_H_29_BN_2_O_2_ | C_34_H_33_BN_2_O_2_ | C_47_H_45_BN_2_OSi | C_15_H_17_NO_4_ |
| Formula weight | 683.58 | 496.39 | 512.43 | 692.75 | 275.29 |
| Temperature/K | 95.15 | 100.15 | 100.00(10) | 100.01(10) | 100.01(10) |
| Crystal system | triclinic | triclinic | orthorhombic | triclinic | monoclinic |
| Space group | *P*-1 | *P*-1 | *P*2_1_2_1_2_1_ | *P*-1 | *P*2_1_/*c* |
| a/Å | 7.5121(11) | 7.4084(2) | 7.78120(10) | 9.56270(10) | 8.6451(2) |
| b/Å | 11.8460(14) | 11.8360(2) | 12.1047(2) | 13.7240(2) | 18.3161(3) |
| c/Å | 20.973(3) | 16.3982(4) | 28.9845(4) | 15.5040(2) | 8.50280(10) |
| α/° | 77.867(11) | 70.095(2) | 90 | 75.2780(10) | 90 |
| β/° | 82.662(12) | 87.089(2) | 90 | 87.6880(10) | 96.968(2) |
| γ/° | 71.745(12) | 73.519(2) | 90 | 70.3600(10) | 90 |
| Volume/Å^3^ | 1729.0(4) | 1294.77(6) | 2730.02(7) | 1851.16(4) | 1336.43(4) |
| Z | 2 | 2 | 4 | 2 | 4 |
| ρ_calc_g/cm^3^ | 1.313 | 1.273 | 1.247 | 1.243 | 1.368 |
| μ/mm^-1^ | 0.666 | 0.615 | 0.597 | 0.855 | 0.823 |
| F(000) | 720.0 | 524.0 | 1088.0 | 736.0 | 584.0 |
| Crystal size/mm^3^ | 0.1 × 0.044 × 0.032 | 0.374 × 0.294 × 0.182 | 0.183 × 0.122 × 0.063 | 0.496 × 0.341 × 0.037 | 0.411 × 0.304 × 0.194 |
| Radiation | CuKα (λ = 1.54184) | CuKα (λ = 1.54184) | Cu Kα (λ = 1.54184) | Cu Kα (λ = 1.54184) | Cu Kα (λ = 1.54184) |
| 2Θ range for data collection/° | 7.99 to 143.984 | 8.286 to 150.992 | 7.916 to 155.3 | 7.074 to 147.984 | 9.658 to 148.96 |
| Index ranges | -9 ≤ *h* ≤ 9,  -13 ≤ *k* ≤ 14,  -25 ≤ *l* ≤ 25 | -9 ≤ *h* ≤ 9,  -13 ≤ *k* ≤ 14,  -20 ≤ *l* ≤ 20 | -6 ≤ *h* ≤ 9,  -15 ≤ *k* ≤ 15,  -36 ≤ *l* ≤ 36 | -11 ≤ *h* ≤ 10,  -17 ≤ *k* ≤ 17,  -19 ≤ *l* ≤ 19 | -10 ≤ *h* ≤ 10,  -22 ≤ *k* ≤ 22,  -7 ≤ *l* ≤ 10 |
| Reflections collected | 18070 | 26848 | 11842 | 26149 | 9432 |
| Independent reflections | 6770 [*R*_int_ = 0.1321, *R*_sigma_ = 0.1453] | 5344 [*R*_int_ = 0.0276, *R*_sigma_ = 0.0210] | 5416 [*R*_int_ = 0.0247, *R*_sigma_ = 0.0351] | 7486 [*R*_int_ = 0.0398, *R*_sigma_ = 0.0414] | 2726 [*R*_int_ = 0.0249, *R*_sigma_ = 0.0251] |
| Data/restraints/parameters | 6770/0/476 | 5344/0/349 | 5416/0/359 | 7486/0/476 | 2726/0/185 |
| Goodness-of-fit on F^2^ | 1.056 | 1.026 | 1.055 | 1.080 | 1.041 |
| Final R indexes [I>=2σ (I)] | *R*_1_ = 0.1339,  *wR*_2_ = 0.3819 | *R*_1_ = 0.0373,  *wR*_2_ = 0.0952 | *R*_1_ = 0.0336,  *wR*_2_ = 0.0812 | *R*_1_ = 0.0393,  *wR*_2_ = 0.1040 | *R*_1_ = 0.0339,  *wR*_2_ = 0.0946 |
| Final R indexes [all data] | *R*_1_ = 0.2161,  *wR*_2_ = 0.4228 | *R*_1_ = 0.0383,  *wR*_2_ = 0.0970 | *R*_1_ = 0.0371,  *wR*_2_ = 0.0841 | *R*_1_ = 0.0436,  *wR*_2_ = 0.1080 | *R*_1_ = 0.0368,  *wR*_2_ = 0.0967 |
| Largest diff. peak/hole / e Å^-3^ | 0.46/-0.46 | 0.36/-0.19 | 0.20/-0.14 | 0.38/-0.35 | 0.29/-0.19 |
| Flack parameter | - | - | -0.05(13) | - | - |

# Theoretical computations


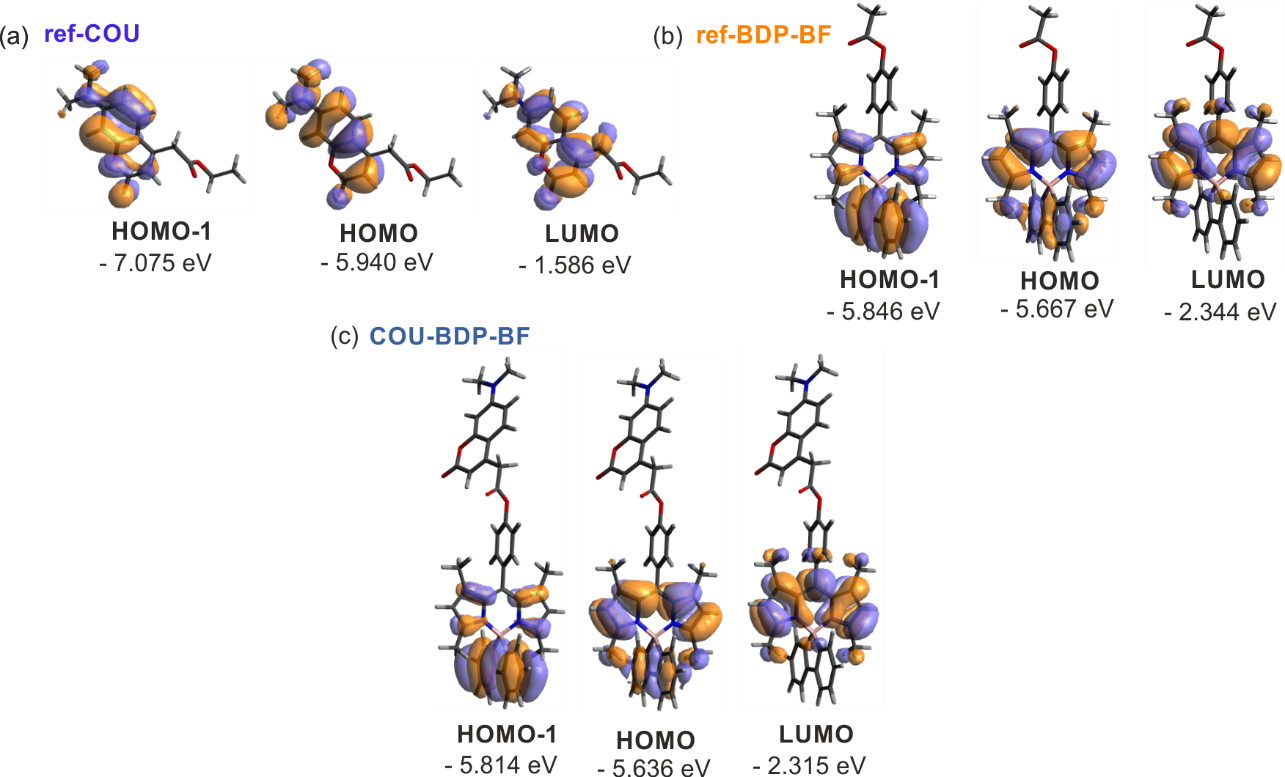


1. Frontier molecular orbitals for (a) **ref-COU**, (b) **ref-BDP-BF** and (c) **COU-BDP-BF**.


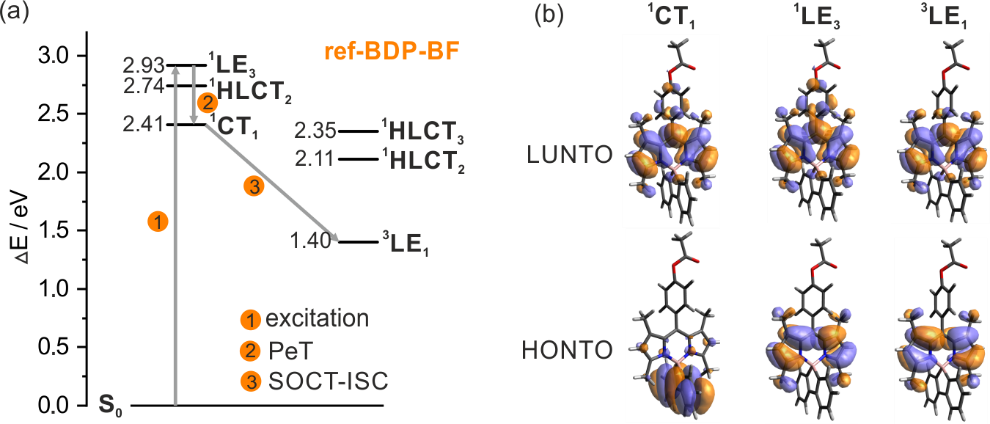


1. (a) Calculated energy diagram demonstrating the photophysical processes in **ref-BDP-BF**. (b) Visualization of natural transition orbitals in **ref-BDP-BF**.


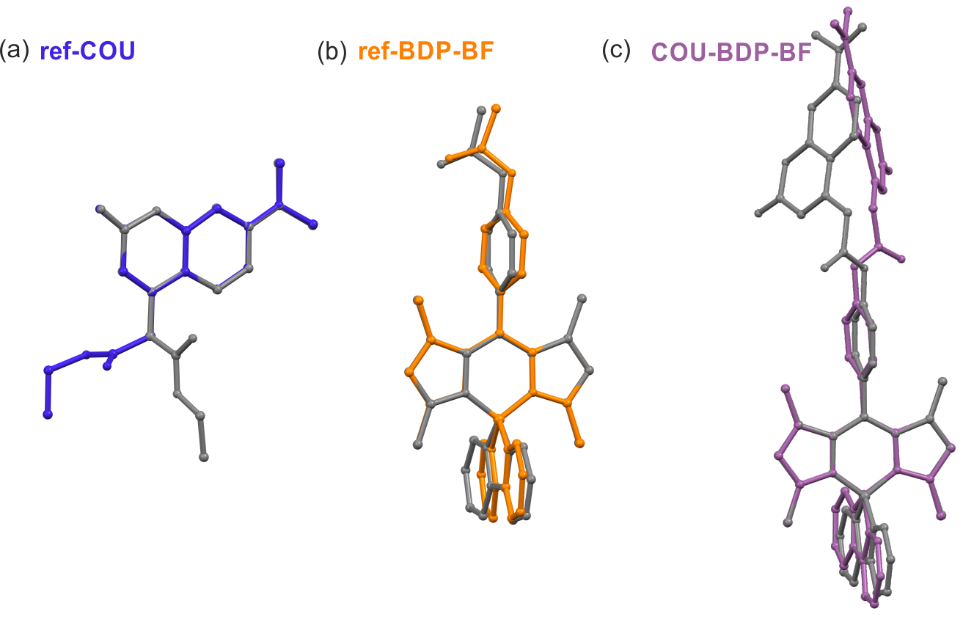


1. Overlay of (a) **ref-COU**, (b) **ref-BDP-BF** and (c) **COU-BDP-BF** structures taken from X-ray diffraction studies and after DFT optimization in ground state (grey).
2. Atomic coordinates for optimized structure of **ref-COU** in its ground electronic state

|  | x | y | z |  | x | y | z |  | x | y | z |
| --- | --- | --- | --- | --- | --- | --- | --- | --- | --- | --- | --- |
| O | 7.2061 | 12.3995 | 9.1420 | H | 8.1974 | 14.5996 | 8.5271 | H | 8.9676 | 17.9100 | 11.7053 |
| O | 3.9563 | 10.1513 | 13.2847 | C | 6.1227 | 12.0728 | 11.6939 | H | 7.4443 | 18.4264 | 10.9467 |
| O | 6.6643 | 10.3169 | 8.6289 | C | 6.7131 | 13.3286 | 11.3063 | C | 5.5450 | 11.9464 | 13.0798 |
| O | 6.0900 | 9.8208 | 13.9111 | C | 6.7955 | 14.4713 | 12.1158 | H | 6.2965 | 12.2601 | 13.8104 |
| N | 8.4895 | 16.8910 | 9.9228 | H | 6.3935 | 14.4422 | 13.1230 | H | 4.6819 | 12.6131 | 13.1702 |
| C | 6.0926 | 11.0578 | 10.7991 | C | 5.0812 | 10.5541 | 13.4275 | C | 5.7929 | 8.4553 | 14.2577 |
| H | 5.6480 | 10.0959 | 11.0214 | C | 7.2404 | 13.4384 | 10.0104 | H | 6.7486 | 7.9389 | 14.1570 |
| C | 6.6461 | 11.1771 | 9.4673 | C | 7.9055 | 15.7361 | 10.3661 | H | 5.0866 | 8.0536 | 13.5280 |
| C | 8.9440 | 16.9857 | 8.5549 | C | 7.3669 | 15.6426 | 11.6733 | C | 5.2484 | 8.3492 | 15.6637 |
| H | 9.6958 | 16.2205 | 8.3319 | H | 7.3976 | 16.4943 | 12.3396 | H | 5.1004 | 7.2965 | 15.9213 |
| H | 9.4061 | 17.9598 | 8.3986 | C | 8.4665 | 18.0780 | 10.7455 | H | 5.9457 | 8.7820 | 16.3857 |
| H | 8.1209 | 16.8775 | 7.8355 | H | 9.0000 | 18.8767 | 10.2314 | H | 4.2857 | 8.8576 | 15.7480 |
| C | 7.8214 | 14.6038 | 9.5410 |  |  |  |  |  |  |  |  |

1. Atomic coordinates for optimized structure of **ref-BDP-BF** in its ground electronic state

|  | x | y | z |  | x | y | z |  | x | y | z |
| --- | --- | --- | --- | --- | --- | --- | --- | --- | --- | --- | --- |
| O | 4.7445 | 9.8564 | 12.6205 | C | 4.2035 | -0.5042 | 17.5254 | C | 7.5329 | 6.0077 | 17.6112 |
| O | 5.6525 | 10.0905 | 14.6772 | C | 5.8757 | 7.7085 | 14.2856 | H | 7.7509 | 6.2226 | 16.5618 |
| N | 3.3156 | 2.8121 | 16.3907 | H | 6.4069 | 7.8849 | 13.3586 | H | 8.4759 | 6.0195 | 18.1625 |
| N | 5.4495 | 2.8856 | 17.7060 | C | 5.6481 | 6.4152 | 14.7358 | H | 6.9157 | 6.8313 | 17.9801 |
| C | 5.6451 | 4.1897 | 17.2465 | H | 6.0117 | 5.5734 | 14.1551 | C | 6.6521 | 1.2609 | 19.1834 |
| C | 3.5719 | 4.1197 | 15.9712 | C | 1.5360 | 1.0878 | 16.0356 | H | 5.8131 | 1.0570 | 19.8548 |
| C | 5.4000 | 8.7758 | 15.0344 | H | 2.1998 | 0.2861 | 15.6997 | H | 7.5755 | 1.2682 | 19.7647 |
| C | 1.6415 | 3.4637 | 15.0473 | H | 0.6025 | 1.0319 | 15.4733 | H | 6.6932 | 0.4375 | 18.4646 |
| H | 0.7177 | 3.4158 | 14.4871 | H | 1.3235 | 0.9044 | 17.0928 | C | 3.4367 | 1.4336 | 18.6628 |
| C | 4.7154 | 8.5659 | 16.2215 | C | 4.9585 | 6.1812 | 15.9246 | C | 5.1717 | -2.1253 | 16.0413 |
| H | 4.3624 | 9.4198 | 16.7891 | C | 5.3871 | 0.2333 | 15.5614 | H | 5.3702 | -3.1591 | 15.7753 |
| C | 6.8700 | 4.6824 | 17.7908 | H | 5.7577 | 1.0256 | 14.9146 | C | 3.4686 | 0.0253 | 18.6897 |
| C | 4.7182 | 4.7914 | 16.3945 | C | 4.4506 | -1.8340 | 17.1971 | C | 2.5066 | 4.5382 | 15.1155 |
| C | 2.1587 | 2.4192 | 15.8364 | H | 4.0897 | -2.6420 | 17.8276 | C | 2.8598 | -0.6876 | 19.7186 |
| C | 6.4855 | 2.5651 | 18.4967 | C | 4.4980 | 7.2678 | 16.6646 | H | 2.8853 | -1.7737 | 19.7377 |
| C | 7.3735 | 3.6545 | 18.5646 | H | 3.9632 | 7.0962 | 17.5933 | C | 2.2101 | 0.0089 | 20.7352 |
| H | 8.2937 | 3.6673 | 19.1325 | C | 2.7838 | 2.1087 | 19.6858 | H | 1.7317 | -0.5369 | 21.5428 |
| C | 2.2984 | 5.8372 | 14.4109 | H | 2.7453 | 3.1959 | 19.6877 | B | 4.2275 | 1.9719 | 17.3488 |
| H | 2.2559 | 6.6814 | 15.1040 | C | 2.1708 | 1.4002 | 20.7214 | C | 5.6619 | 11.9787 | 13.2917 |
| H | 1.3558 | 5.8045 | 13.8599 | H | 1.6613 | 1.9325 | 21.5193 | H | 5.4058 | 12.3176 | 12.2901 |
| H | 3.0975 | 6.0564 | 13.6980 | C | 5.6388 | -1.0986 | 15.2256 | H | 5.1292 | 12.5763 | 14.0356 |
| C | 4.6704 | 0.5451 | 16.7093 | H | 6.1996 | -1.3377 | 14.3268 | H | 6.7311 | 12.1085 | 13.4744 |
| C | 5.2881 | 10.5373 | 13.4407 |  |  |  |  |  |  |  |  |

1. Atomic coordinates for optimized structure of **ref-BDP-BF** in its S_1_ singlet excited electronic state

|  | x | y | z |  | x | y | z |  | x | y | z |
| --- | --- | --- | --- | --- | --- | --- | --- | --- | --- | --- | --- |
| O | 4.2642 | 10.1079 | 12.9024 | C | 4.2199 | -0.4915 | 17.5752 | C | 7.6544 | 5.9050 | 17.5003 |
| O | 5.7755 | 10.0910 | 14.5839 | C | 5.6018 | 7.7210 | 14.0890 | H | 7.6534 | 6.1761 | 16.4418 |
| N | 3.3724 | 2.7967 | 16.3184 | H | 5.8812 | 7.8951 | 13.0568 | H | 8.6944 | 5.7795 | 17.8154 |
| N | 5.4135 | 2.9258 | 17.7636 | C | 5.3599 | 6.4354 | 14.5524 | H | 7.2500 | 6.7667 | 18.0439 |
| C | 5.6623 | 4.2010 | 17.2551 | H | 5.4590 | 5.5944 | 13.8738 | C | 6.5618 | 1.3029 | 19.3474 |
| C | 3.5973 | 4.1169 | 15.9265 | C | 1.6025 | 1.0176 | 15.9129 | H | 5.7150 | 1.1327 | 20.0208 |
| C | 5.4743 | 8.7871 | 14.9670 | H | 2.2728 | 0.2199 | 15.5756 | H | 7.4656 | 1.3327 | 19.9597 |
| C | 1.6629 | 3.4118 | 15.0127 | H | 0.6940 | 0.9581 | 15.3098 | H | 6.6414 | 0.4299 | 18.6906 |
| H | 0.7353 | 3.3706 | 14.4569 | H | 1.3217 | 0.7967 | 16.9482 | C | 3.3789 | 1.4587 | 18.6040 |
| C | 5.1210 | 8.5812 | 16.2911 | C | 5.0007 | 6.1907 | 15.8826 | C | 5.2814 | -2.1421 | 16.2067 |
| H | 5.0312 | 9.4337 | 16.9557 | C | 5.5193 | 0.2323 | 15.6694 | H | 5.5148 | -3.1725 | 15.9616 |
| C | 6.8990 | 4.6442 | 17.7688 | H | 5.9244 | 1.0099 | 15.0302 | C | 3.4330 | 0.0262 | 18.6529 |
| C | 4.7496 | 4.8164 | 16.3626 | C | 4.4967 | -1.8471 | 17.2949 | C | 2.5163 | 4.5253 | 15.1172 |
| C | 2.1946 | 2.3740 | 15.7492 | H | 4.1017 | -2.6405 | 17.9207 | C | 2.7722 | -0.7117 | 19.6588 |
| C | 6.4518 | 2.5845 | 18.5974 | C | 4.8894 | 7.2883 | 16.7414 | H | 2.8229 | -1.7952 | 19.6833 |
| C | 7.3624 | 3.6200 | 18.6148 | H | 4.6080 | 7.1204 | 17.7759 | C | 2.0589 | -0.0249 | 20.6108 |
| H | 8.2847 | 3.6297 | 19.1810 | C | 2.6478 | 2.1150 | 19.5829 | H | 1.5384 | -0.5594 | 21.3981 |
| C | 2.2333 | 5.8508 | 14.4887 | H | 2.5830 | 3.1981 | 19.5767 | B | 4.2405 | 2.0275 | 17.3304 |
| H | 2.4315 | 6.6863 | 15.1642 | C | 1.9968 | 1.3865 | 20.5722 | C | 5.6108 | 12.0559 | 13.3268 |
| H | 1.1804 | 5.8971 | 14.1961 | H | 1.4251 | 1.9037 | 21.3370 | H | 5.1074 | 12.4911 | 12.4658 |
| H | 2.8300 | 6.0321 | 13.5877 | C | 5.7922 | -1.1034 | 15.3957 | H | 5.4135 | 12.6572 | 14.2178 |
| C | 4.7312 | 0.5713 | 16.7590 | H | 6.4106 | -1.3651 | 14.5423 | H | 6.6920 | 12.0502 | 13.1706 |
| C | 5.1159 | 10.6573 | 13.5409 |  |  |  |  |  |  |  |  |

1. Atomic coordinates for optimized structure of **ref-BDP-BF** in its S_2_ singlet excited electronic state

|  | x | y | z |  | x | y | z |  | x | y | z |
| --- | --- | --- | --- | --- | --- | --- | --- | --- | --- | --- | --- |
| O | 4.1340 | 10.1336 | 13.0137 | C | 4.2345 | -0.4814 | 17.5597 | C | 7.7243 | 5.8083 | 17.4663 |
| O | 5.7924 | 10.0748 | 14.5487 | C | 5.5143 | 7.7164 | 14.0531 | H | 7.6588 | 6.0956 | 16.4148 |
| N | 3.4065 | 2.7898 | 16.3086 | H | 5.7110 | 7.8929 | 13.0024 | H | 8.7736 | 5.6235 | 17.7118 |
| N | 5.3879 | 2.9307 | 17.7857 | C | 5.2759 | 6.4339 | 14.5233 | H | 7.4004 | 6.6796 | 18.0485 |
| C | 5.6682 | 4.1929 | 17.2687 | H | 5.2970 | 5.5983 | 13.8310 | C | 6.4695 | 1.2955 | 19.4209 |
| C | 3.6138 | 4.1105 | 15.9264 | C | 1.6711 | 0.9641 | 15.8756 | H | 5.6049 | 1.1827 | 20.0814 |
| C | 5.4949 | 8.7777 | 14.9474 | H | 2.3736 | 0.2035 | 15.5224 | H | 7.3713 | 1.3016 | 20.0364 |
| C | 1.6715 | 3.3725 | 15.0280 | H | 0.7593 | 0.8907 | 15.2796 | H | 6.5045 | 0.4136 | 18.7749 |
| H | 0.7409 | 3.3328 | 14.4785 | H | 1.4209 | 0.7212 | 16.9124 | C | 3.3450 | 1.4467 | 18.6008 |
| C | 5.2546 | 8.5663 | 16.2965 | C | 5.0259 | 6.1844 | 15.8799 | C | 5.3675 | -2.0777 | 16.1661 |
| H | 5.2443 | 9.4141 | 16.9730 | C | 5.5764 | 0.2862 | 15.6930 | H | 5.6117 | -3.1067 | 15.9217 |
| C | 6.9162 | 4.5956 | 17.7715 | H | 5.9803 | 1.0896 | 15.0842 | C | 3.4036 | 0.0461 | 18.6439 |
| C | 4.7694 | 4.8196 | 16.3641 | C | 4.5422 | -1.8041 | 17.2536 | C | 2.5164 | 4.5104 | 15.1489 |
| C | 2.2181 | 2.3368 | 15.7352 | H | 4.1471 | -2.6173 | 17.8559 | C | 2.7183 | -0.6441 | 19.6398 |
| C | 6.4173 | 2.5602 | 18.6447 | C | 5.0254 | 7.2772 | 16.7559 | H | 2.7567 | -1.7288 | 19.6874 |
| C | 7.3497 | 3.5647 | 18.6456 | H | 4.8232 | 7.1091 | 17.8088 | C | 1.9820 | 0.0704 | 20.5810 |
| H | 8.2712 | 3.5612 | 19.2118 | C | 2.6171 | 2.1581 | 19.5353 | H | 1.4450 | -0.4612 | 21.3602 |
| C | 2.1851 | 5.8414 | 14.5718 | H | 2.5717 | 3.2426 | 19.5006 | B | 4.2473 | 2.0594 | 17.3228 |
| H | 2.4695 | 6.6636 | 15.2313 | C | 1.9290 | 1.4598 | 20.5323 | C | 5.5584 | 12.0499 | 13.3152 |
| H | 1.1106 | 5.9040 | 14.3803 | H | 1.3489 | 2.0065 | 21.2695 | H | 4.9863 | 12.5019 | 12.5075 |
| H | 2.6969 | 6.0191 | 13.6182 | C | 5.8828 | -1.0441 | 15.3905 | H | 5.4616 | 12.6505 | 14.2230 |
| C | 4.7549 | 0.5550 | 16.7710 | H | 6.5249 | -1.2693 | 14.5444 | H | 6.6194 | 12.0192 | 13.0566 |
| C | 5.0527 | 10.6633 | 13.5701 |  |  |  |  |  |  |  |  |

1. Atomic coordinates for optimized structure of **ref-BDP-BF** in its S_3_ singlet excited electronic state

|  | x | y | z |  | x | y | z |  | x | y | z |
| --- | --- | --- | --- | --- | --- | --- | --- | --- | --- | --- | --- |
| O | 4.2719 | 10.1084 | 12.8997 | C | 4.2173 | -0.4917 | 17.5732 | C | 7.6564 | 5.9023 | 17.4983 |
| O | 5.7778 | 10.0912 | 14.5861 | C | 5.6018 | 7.7213 | 14.0898 | H | 7.6544 | 6.1739 | 16.4399 |
| N | 3.3707 | 2.7979 | 16.3195 | H | 5.8803 | 7.8955 | 13.0574 | H | 8.6967 | 5.7758 | 17.8121 |
| N | 5.4133 | 2.9248 | 17.7627 | C | 5.3594 | 6.4358 | 14.5530 | H | 7.2533 | 6.7641 | 18.0427 |
| C | 5.6627 | 4.2000 | 17.2544 | H | 5.4572 | 5.5949 | 13.8739 | C | 6.5618 | 1.3004 | 19.3449 |
| C | 3.5963 | 4.1181 | 15.9280 | C | 1.5990 | 1.0204 | 15.9150 | H | 5.7159 | 1.1311 | 20.0195 |
| C | 5.4760 | 8.7872 | 14.9683 | H | 2.2682 | 0.2222 | 15.5764 | H | 7.4667 | 1.3291 | 19.9558 |
| C | 1.6604 | 3.4148 | 15.0157 | H | 0.6897 | 0.9619 | 15.3130 | H | 6.6394 | 0.4275 | 18.6878 |
| H | 0.7322 | 3.3746 | 14.4608 | H | 1.3193 | 0.7991 | 16.9506 | C | 3.3787 | 1.4584 | 18.6043 |
| C | 5.1238 | 8.5809 | 16.2927 | C | 5.0013 | 6.1907 | 15.8834 | C | 5.2762 | -2.1421 | 16.2024 |
| H | 5.0354 | 9.4333 | 16.9577 | C | 5.5153 | 0.2326 | 15.6666 | H | 5.5085 | -3.1725 | 15.9563 |
| C | 6.9003 | 4.6420 | 17.7670 | H | 5.9203 | 1.0103 | 15.0276 | C | 3.4319 | 0.0259 | 18.6521 |
| C | 4.7496 | 4.8164 | 16.3631 | C | 4.4928 | -1.8473 | 17.2916 | C | 2.5148 | 4.5276 | 15.1198 |
| C | 2.1920 | 2.3764 | 15.7513 | H | 4.0978 | -2.6408 | 17.9173 | C | 2.7718 | -0.7123 | 19.6582 |
| C | 6.4522 | 2.5824 | 18.5954 | C | 4.8917 | 7.2881 | 16.7427 | H | 2.8217 | -1.7959 | 19.6820 |
| C | 7.3637 | 3.6171 | 18.6123 | H | 4.6111 | 7.1201 | 17.7775 | C | 2.0600 | -0.0257 | 20.6116 |
| H | 8.2865 | 3.6259 | 19.1776 | C | 2.6493 | 2.1146 | 19.5845 | H | 1.5400 | -0.5603 | 21.3991 |
| C | 2.2323 | 5.8536 | 14.4921 | H | 2.5852 | 3.1977 | 19.5791 | B | 4.2393 | 2.0275 | 17.3301 |
| H | 2.4318 | 6.6887 | 15.1678 | C | 1.9989 | 1.3858 | 20.5740 | C | 5.6173 | 12.0562 | 13.3287 |
| H | 1.1791 | 5.9009 | 14.2007 | H | 1.4283 | 1.9029 | 21.3397 | H | 5.1156 | 12.4921 | 12.4671 |
| H | 2.8282 | 6.0348 | 13.5906 | C | 5.7869 | -1.1031 | 15.3917 | H | 5.4188 | 12.6570 | 14.2197 |
| C | 4.7286 | 0.5714 | 16.7573 | H | 6.4043 | -1.3647 | 14.5375 | H | 6.6988 | 12.0501 | 13.1742 |
| C | 5.1215 | 10.6576 | 13.5411 |  |  |  |  |  |  |  |  |

1. Atomic coordinates for optimized structure of **ref-BDP-BF** in its T_1_ triplet excited electronic state

|  | x | y | z |  | x | y | z |  | x | y | z |
| --- | --- | --- | --- | --- | --- | --- | --- | --- | --- | --- | --- |
| O | 4.6750 | 9.8979 | 12.6424 | C | 4.1978 | -0.5088 | 17.5291 | C | 7.5443 | 5.9860 | 17.6207 |
| O | 5.6674 | 10.1225 | 14.6604 | C | 5.8886 | 7.7402 | 14.2762 | H | 7.7580 | 6.1896 | 16.5673 |
| N | 3.3340 | 2.8231 | 16.3897 | H | 6.4188 | 7.9173 | 13.3482 | H | 8.4863 | 6.0199 | 18.1730 |
| N | 5.4468 | 2.8920 | 17.6991 | C | 5.6616 | 6.4477 | 14.7305 | H | 6.9127 | 6.8073 | 17.9727 |
| C | 5.6424 | 4.1625 | 17.2532 | H | 6.0270 | 5.6066 | 14.1491 | C | 6.6515 | 1.2400 | 19.1952 |
| C | 3.5820 | 4.0933 | 15.9824 | C | 1.5357 | 1.0710 | 16.0291 | H | 5.8031 | 1.0415 | 19.8578 |
| C | 5.4138 | 8.8079 | 15.0240 | H | 2.2095 | 0.2744 | 15.6977 | H | 7.5703 | 1.2285 | 19.7839 |
| C | 1.6253 | 3.4454 | 15.0321 | H | 0.6003 | 0.9982 | 15.4718 | H | 6.6840 | 0.4200 | 18.4708 |
| H | 0.7030 | 3.3981 | 14.4698 | H | 1.3322 | 0.8905 | 17.0894 | C | 3.4334 | 1.4329 | 18.6625 |
| C | 4.7314 | 8.5983 | 16.2120 | C | 4.9720 | 6.2008 | 15.9206 | C | 5.1652 | -2.1347 | 16.0498 |
| H | 4.3787 | 9.4525 | 16.7797 | C | 5.3892 | 0.2224 | 15.5681 | H | 5.3613 | -3.1695 | 15.7858 |
| C | 6.9002 | 4.6589 | 17.8165 | H | 5.7646 | 1.0129 | 14.9215 | C | 3.4606 | 0.0242 | 18.6914 |
| C | 4.7309 | 4.8116 | 16.3908 | C | 4.4415 | -1.8398 | 17.2035 | C | 2.4865 | 4.5182 | 15.1016 |
| C | 2.1353 | 2.4048 | 15.8164 | H | 4.0767 | -2.6463 | 17.8337 | C | 2.8470 | -0.6866 | 19.7186 |
| C | 6.5122 | 2.5480 | 18.5208 | C | 4.5156 | 7.2985 | 16.6529 | H | 2.8693 | -1.7728 | 19.7391 |
| C | 7.3966 | 3.6314 | 18.5884 | H | 3.9806 | 7.1291 | 17.5824 | C | 2.1960 | 0.0125 | 20.7330 |
| H | 8.3157 | 3.6416 | 19.1580 | C | 2.7789 | 2.1096 | 19.6840 | H | 1.7137 | -0.5315 | 21.5395 |
| C | 2.3042 | 5.8239 | 14.4129 | H | 2.7435 | 3.1972 | 19.6854 | B | 4.2303 | 1.9683 | 17.3505 |
| H | 2.2664 | 6.6598 | 15.1176 | C | 2.1605 | 1.4039 | 20.7184 | C | 5.6432 | 12.0095 | 13.2740 |
| H | 1.3698 | 5.8118 | 13.8473 | H | 1.6500 | 1.9378 | 21.5147 | H | 5.3480 | 12.3512 | 12.2841 |
| H | 3.1199 | 6.0444 | 13.7179 | C | 5.6380 | -1.1107 | 15.2341 | H | 5.1507 | 12.6137 | 14.0399 |
| C | 4.6699 | 0.5390 | 16.7136 | H | 6.2008 | -1.3530 | 14.3373 | H | 6.7208 | 12.1265 | 13.4098 |
| C | 5.2591 | 10.5721 | 13.4404 |  |  |  |  |  |  |  |  |

1. Atomic coordinates for optimized structure of **ref-BDP-BF** in its T_2_ triplet excited electronic state

|  | x | y | z |  | x | y | z |  | x | y | z |
| --- | --- | --- | --- | --- | --- | --- | --- | --- | --- | --- | --- |
| O | 4.3934 | 9.9980 | 12.8023 | C | 4.2194 | -0.4698 | 17.5375 | C | 7.6249 | 5.9100 | 17.5874 |
| O | 5.7321 | 10.0854 | 14.6215 | C | 5.7013 | 7.7072 | 14.1512 | H | 7.6607 | 6.1983 | 16.5339 |
| N | 3.3629 | 2.8039 | 16.3571 | H | 6.0654 | 7.8789 | 13.1456 | H | 8.6513 | 5.8152 | 17.9511 |
| N | 5.4241 | 2.9035 | 17.7353 | C | 5.4656 | 6.4185 | 14.6105 | H | 7.1610 | 6.7463 | 18.1238 |
| C | 5.6630 | 4.1870 | 17.2616 | H | 5.6559 | 5.5738 | 13.9561 | C | 6.5666 | 1.2363 | 19.2991 |
| C | 3.5931 | 4.1140 | 15.9449 | C | 1.5930 | 0.9981 | 15.9744 | H | 5.7156 | 1.0681 | 19.9653 |
| C | 5.4531 | 8.7785 | 14.9970 | H | 2.2703 | 0.2097 | 15.6345 | H | 7.4770 | 1.2369 | 19.9020 |
| C | 1.6551 | 3.3744 | 15.0335 | H | 0.6706 | 0.9292 | 15.3940 | H | 6.6147 | 0.3892 | 18.6090 |
| H | 0.7317 | 3.3283 | 14.4718 | H | 1.3526 | 0.7954 | 17.0218 | C | 3.3957 | 1.4642 | 18.6272 |
| C | 4.9888 | 8.5760 | 16.2872 | C | 4.9955 | 6.1834 | 15.9048 | C | 5.2622 | -2.0786 | 16.0897 |
| H | 4.8079 | 9.4328 | 16.9271 | C | 5.4709 | 0.2823 | 15.6078 | H | 5.4820 | -3.1102 | 15.8333 |
| C | 6.8947 | 4.6298 | 17.8020 | H | 5.8537 | 1.0805 | 14.9785 | C | 3.4428 | 0.0610 | 18.6625 |
| C | 4.7459 | 4.8024 | 16.3807 | C | 4.4958 | -1.7957 | 17.2173 | C | 2.5154 | 4.5064 | 15.1180 |
| C | 2.1703 | 2.3531 | 15.7827 | H | 4.1197 | -2.6053 | 17.8362 | C | 2.7977 | -0.6333 | 19.6818 |
| C | 6.4705 | 2.5355 | 18.5865 | C | 4.7656 | 7.2807 | 16.7363 | H | 2.8282 | -1.7186 | 19.7185 |
| C | 7.3605 | 3.5745 | 18.6311 | H | 4.3983 | 7.1154 | 17.7441 | C | 2.1073 | 0.0779 | 20.6597 |
| H | 8.2784 | 3.5818 | 19.2034 | C | 2.7061 | 2.1673 | 19.5990 | H | 1.6017 | -0.4566 | 21.4578 |
| C | 2.2491 | 5.8092 | 14.4480 | H | 2.6643 | 3.2524 | 19.5750 | B | 4.2467 | 2.0484 | 17.3233 |
| H | 2.4239 | 6.6624 | 15.1079 | C | 2.0598 | 1.4679 | 20.6215 | C | 5.6025 | 12.0174 | 13.3061 |
| H | 1.2104 | 5.8461 | 14.1101 | H | 1.5185 | 2.0120 | 21.3894 | H | 5.1660 | 12.4125 | 12.3911 |
| H | 2.8858 | 5.9627 | 13.5688 | C | 5.7479 | -1.0496 | 15.2889 | H | 5.2931 | 12.6250 | 14.1601 |
| C | 4.7110 | 0.5675 | 16.7284 | H | 6.3454 | -1.2821 | 14.4127 | H | 6.6929 | 12.0589 | 13.2546 |
| C | 5.1524 | 10.6029 | 13.5031 |  |  |  |  |  |  |  |  |

1. Atomic coordinates for optimized structure of **ref-BDP-BF** in its T_3_ triplet excited electronic state

|  | x | y | z |  | x | y | z |  | x | y | z |
| --- | --- | --- | --- | --- | --- | --- | --- | --- | --- | --- | --- |
| O | 4.3021 | 10.0807 | 12.8706 | C | 4.2177 | -0.4879 | 17.5659 | C | 7.6408 | 5.9121 | 17.5223 |
| O | 5.7646 | 10.0904 | 14.5946 | C | 5.6291 | 7.7174 | 14.1042 | H | 7.6455 | 6.1920 | 16.4662 |
| N | 3.3642 | 2.7955 | 16.3283 | H | 5.9326 | 7.8907 | 13.0787 | H | 8.6792 | 5.7928 | 17.8448 |
| N | 5.4174 | 2.9162 | 17.7574 | C | 5.3882 | 6.4310 | 14.5663 | H | 7.2245 | 6.7657 | 18.0699 |
| C | 5.6613 | 4.1992 | 17.2550 | H | 5.5126 | 5.5886 | 13.8935 | C | 6.5662 | 1.2876 | 19.3343 |
| C | 3.5943 | 4.1169 | 15.9300 | C | 1.5953 | 1.0161 | 15.9294 | H | 5.7207 | 1.1141 | 20.0080 |
| C | 5.4682 | 8.7853 | 14.9746 | H | 2.2649 | 0.2172 | 15.5942 | H | 7.4729 | 1.3134 | 19.9426 |
| C | 1.6623 | 3.4045 | 15.0145 | H | 0.6839 | 0.9557 | 15.3309 | H | 6.6403 | 0.4208 | 18.6693 |
| H | 0.7361 | 3.3617 | 14.4563 | H | 1.3222 | 0.8020 | 16.9681 | C | 3.3876 | 1.4621 | 18.6091 |
| C | 5.0831 | 8.5806 | 16.2900 | C | 4.9973 | 6.1888 | 15.8872 | C | 5.2710 | -2.1280 | 16.1799 |
| H | 4.9682 | 9.4347 | 16.9488 | C | 5.5104 | 0.2420 | 15.6538 | H | 5.5003 | -3.1585 | 15.9303 |
| C | 6.8920 | 4.6452 | 17.7749 | H | 5.9119 | 1.0245 | 15.0177 | C | 3.4360 | 0.0320 | 18.6549 |
| C | 4.7466 | 4.8123 | 16.3662 | C | 4.4913 | -1.8378 | 17.2764 | C | 2.5187 | 4.5223 | 15.1150 |
| C | 2.1897 | 2.3698 | 15.7583 | H | 4.1005 | -2.6355 | 17.8998 | C | 2.7800 | -0.6986 | 19.6632 |
| C | 6.4525 | 2.5733 | 18.5930 | C | 4.8529 | 7.2870 | 16.7393 | H | 2.8238 | -1.7826 | 19.6892 |
| C | 7.3577 | 3.6127 | 18.6191 | H | 4.5469 | 7.1198 | 17.7669 | C | 2.0768 | -0.0061 | 20.6226 |
| H | 8.2784 | 3.6231 | 19.1878 | C | 2.6684 | 2.1262 | 19.5958 | H | 1.5599 | -0.5403 | 21.4128 |
| C | 2.2413 | 5.8417 | 14.4732 | H | 2.6118 | 3.2101 | 19.5886 | B | 4.2383 | 2.0205 | 17.3322 |
| H | 2.4338 | 6.6829 | 15.1432 | C | 2.0206 | 1.4039 | 20.5888 | C | 5.6127 | 12.0472 | 13.3226 |
| H | 1.1916 | 5.8863 | 14.1696 | H | 1.4570 | 1.9235 | 21.3580 | H | 5.1280 | 12.4726 | 12.4461 |
| H | 2.8475 | 6.0141 | 13.5767 | C | 5.7801 | -1.0900 | 15.3701 | H | 5.3842 | 12.6501 | 14.2050 |
| C | 4.7254 | 0.5744 | 16.7516 | H | 6.3924 | -1.3489 | 14.5115 | H | 6.6977 | 12.0533 | 13.1958 |
| C | 5.1284 | 10.6441 | 13.5296 |  |  |  |  |  |  |  |  |

1. Atomic coordinates for optimized structure of **COU-BDP-BF** in its ground electronic state

|  | x | y | z |  | x | y | z |  | x | y | z |
| --- | --- | --- | --- | --- | --- | --- | --- | --- | --- | --- | --- |
| O | 6.6249 | 12.1890 | 9.5238 | H | 6.5140 | 14.6148 | 13.3705 | H | 1.5053 | 2.0035 | 21.2387 |
| O | 3.6240 | 10.6993 | 14.2343 | C | 7.3507 | 3.7876 | 18.6102 | C | 5.9003 | -1.0390 | 15.2349 |
| O | 5.7801 | 10.1618 | 9.2544 | H | 8.2333 | 3.8248 | 19.2341 | H | 6.5179 | -1.2799 | 14.3747 |
| O | 5.7942 | 10.1192 | 14.4885 | C | 2.5111 | 5.8209 | 14.1125 | C | 7.5275 | 6.1314 | 17.6371 |
| N | 3.4479 | 2.8459 | 16.2056 | H | 2.4082 | 6.6793 | 14.7812 | H | 7.8350 | 6.3231 | 16.6056 |
| N | 5.4983 | 2.9725 | 17.6439 | H | 1.6095 | 5.7614 | 13.4990 | H | 8.4176 | 6.1778 | 18.2687 |
| C | 5.6988 | 4.2743 | 17.1802 | H | 3.3542 | 6.0364 | 13.4506 | H | 6.8643 | 6.9509 | 17.9263 |
| C | 3.7081 | 4.1518 | 15.7827 | C | 4.8179 | 0.6067 | 16.6353 | C | 6.6348 | 1.3890 | 19.2155 |
| N | 8.5286 | 16.5172 | 9.7737 | C | 4.7876 | 10.9620 | 14.1428 | H | 5.7608 | 1.1803 | 19.8392 |
| C | 5.5388 | 11.1362 | 11.3932 | C | 4.3199 | -0.4399 | 17.4362 | H | 7.5219 | 1.4194 | 19.8501 |
| H | 5.0029 | 10.2614 | 11.7406 | C | 6.0506 | 7.7806 | 14.2272 | H | 6.7313 | 0.5568 | 18.5124 |
| C | 5.4904 | 8.8367 | 14.9277 | H | 6.6523 | 7.9830 | 13.3479 | C | 3.4557 | 1.4988 | 18.4998 |
| C | 1.8502 | 3.4493 | 14.7508 | C | 5.8306 | 6.4817 | 14.6672 | C | 7.4641 | 15.5623 | 11.7313 |
| H | 0.9647 | 3.3769 | 14.1343 | H | 6.2638 | 5.6475 | 14.1248 | H | 7.6752 | 16.4492 | 12.3137 |
| C | 4.7203 | 8.6217 | 16.0609 | C | 1.7212 | 1.0871 | 15.7685 | C | 5.4012 | -2.0630 | 16.0349 |
| H | 4.2875 | 9.4588 | 16.5949 | H | 2.4157 | 0.2919 | 15.4825 | H | 5.6317 | -3.0967 | 15.7951 |
| C | 6.8778 | 4.7966 | 17.7932 | H | 0.8230 | 1.0089 | 15.1540 | C | 3.5084 | 0.0917 | 18.5475 |
| C | 5.9570 | 11.0797 | 10.0087 | H | 1.4509 | 0.9140 | 16.8141 | C | 8.7778 | 17.7420 | 10.4984 |
| C | 8.8649 | 16.4374 | 8.3709 | C | 5.0551 | 6.2394 | 15.7988 | H | 9.3335 | 18.4264 | 9.8586 |
| H | 9.5090 | 15.5753 | 8.1623 | C | 5.6076 | 0.2927 | 15.5369 | H | 9.3805 | 17.5632 | 11.3964 |
| H | 9.4079 | 17.3362 | 8.0817 | H | 6.0040 | 1.0828 | 14.9029 | H | 7.8477 | 18.2408 | 10.8009 |
| H | 7.9717 | 16.3595 | 7.7371 | C | 6.8821 | 13.2771 | 10.2889 | C | 5.3575 | 12.2573 | 13.6290 |
| C | 7.5490 | 14.3158 | 9.6635 | C | 4.6074 | -1.7694 | 17.1413 | H | 6.2135 | 12.5339 | 14.2522 |
| H | 7.8105 | 14.1854 | 8.6224 | H | 4.2221 | -2.5754 | 17.7599 | H | 4.5868 | 13.0248 | 13.7454 |
| C | 4.8142 | 4.8488 | 16.2662 | C | 4.5054 | 7.3188 | 16.4891 | C | 2.6918 | 4.5391 | 14.8552 |
| C | 2.3341 | 2.4255 | 15.5871 | H | 3.9009 | 7.1361 | 17.3717 | C | 2.8508 | -0.6183 | 19.5479 |
| C | 5.7940 | 12.2027 | 12.1869 | C | 7.8620 | 15.4861 | 10.3731 | H | 2.8925 | -1.7036 | 19.5830 |
| C | 6.4902 | 13.3358 | 11.6356 | C | 2.7322 | 2.1756 | 19.4731 | C | 2.1307 | 0.0797 | 20.5147 |
| C | 6.4886 | 2.6811 | 18.5016 | H | 2.6759 | 3.2619 | 19.4579 | H | 1.6137 | -0.4640 | 21.2997 |
| C | 6.8030 | 14.5145 | 12.3297 | C | 2.0700 | 1.4699 | 20.4798 | B | 4.3146 | 2.0338 | 17.2278 |

1. Atomic coordinates for optimized structure of **COU-BDP-BF** in its S_1_ singlet excited electronic state

|  | x | y | z |  | x | y | z |  | x | y | z |
| --- | --- | --- | --- | --- | --- | --- | --- | --- | --- | --- | --- |
| O | 6.0541 | 12.4612 | 9.5105 | H | 7.0421 | 14.5419 | 13.4309 | H | 0.9977 | 1.9569 | 20.8182 |
| O | 3.9628 | 10.8847 | 14.6365 | C | 7.2195 | 3.7355 | 18.8551 | C | 6.1575 | -1.0805 | 15.5683 |
| O | 4.9508 | 10.5549 | 9.3063 | H | 8.0618 | 3.7740 | 19.5335 | H | 6.8839 | -1.3471 | 14.8063 |
| O | 6.0652 | 10.0718 | 14.4588 | C | 2.6179 | 5.7841 | 14.0631 | C | 7.6160 | 6.0011 | 17.7344 |
| N | 3.5737 | 2.7881 | 16.0839 | H | 2.7158 | 6.6362 | 14.7398 | H | 7.7445 | 6.2501 | 16.6782 |
| N | 5.4071 | 2.9871 | 17.7784 | H | 1.6085 | 5.8033 | 13.6425 | H | 8.6099 | 5.8996 | 18.1800 |
| C | 5.6972 | 4.2566 | 17.2779 | H | 3.3174 | 5.9578 | 13.2375 | H | 7.1344 | 6.8669 | 18.2036 |
| C | 3.8219 | 4.1057 | 15.6971 | C | 4.9019 | 0.5997 | 16.7466 | C | 6.3702 | 1.4185 | 19.5310 |
| N | 8.4406 | 16.5516 | 9.6046 | C | 5.1155 | 11.0256 | 14.3437 | H | 5.4472 | 1.2457 | 20.0946 |
| C | 5.2914 | 11.3525 | 11.5055 | C | 4.3100 | -0.4563 | 17.5161 | H | 7.1879 | 1.4778 | 20.2526 |
| H | 4.7537 | 10.5032 | 11.9088 | C | 5.9591 | 7.7304 | 14.0705 | H | 6.5473 | 0.5344 | 18.9092 |
| C | 5.7109 | 8.8005 | 14.9140 | H | 6.3667 | 7.9127 | 13.0819 | C | 3.3055 | 1.4967 | 18.3798 |
| C | 2.0340 | 3.3431 | 14.5585 | C | 5.6904 | 6.4425 | 14.5145 | C | 7.7157 | 15.5366 | 11.6851 |
| H | 1.1863 | 3.2722 | 13.8897 | H | 5.8873 | 5.5970 | 13.8635 | H | 8.1418 | 16.3466 | 12.2618 |
| C | 5.2064 | 8.6077 | 16.1905 | C | 1.9043 | 0.9647 | 15.4926 | C | 5.5679 | -2.1124 | 16.3332 |
| H | 5.0090 | 9.4576 | 16.8334 | H | 2.6273 | 0.1755 | 15.2607 | H | 5.8497 | -3.1425 | 16.1443 |
| C | 6.8511 | 4.7334 | 17.9343 | H | 1.0820 | 0.8747 | 14.7796 | C | 3.3802 | 0.0672 | 18.4701 |
| C | 5.3888 | 11.3799 | 10.0616 | H | 1.4972 | 0.7573 | 16.4880 | C | 8.9340 | 17.6995 | 10.3291 |
| C | 8.4226 | 16.5811 | 8.1606 | C | 5.1791 | 6.2080 | 15.7949 | H | 9.4304 | 18.3731 | 9.6314 |
| H | 8.8739 | 15.6760 | 7.7394 | C | 5.8271 | 0.2545 | 15.7731 | H | 9.6697 | 17.4081 | 11.0870 |
| H | 9.0050 | 17.4338 | 7.8134 | H | 6.2951 | 1.0265 | 15.1711 | H | 8.1291 | 18.2575 | 10.8270 |
| H | 7.4042 | 16.6749 | 7.7592 | C | 6.5894 | 13.4488 | 10.2669 | C | 5.7224 | 12.2908 | 13.7916 |
| C | 7.2156 | 14.4722 | 9.5762 | C | 4.6459 | -1.8112 | 17.3059 | H | 6.7210 | 12.4176 | 14.2207 |
| H | 7.2281 | 14.4096 | 8.4967 | H | 4.1891 | -2.5994 | 17.8952 | H | 5.0987 | 13.1262 | 14.1238 |
| C | 4.8954 | 4.8365 | 16.2632 | C | 4.9452 | 7.3143 | 16.6197 | C | 2.8451 | 4.4764 | 14.7489 |
| C | 2.4868 | 2.3302 | 15.3778 | H | 4.5413 | 7.1506 | 17.6136 | C | 2.6068 | -0.6634 | 19.3981 |
| C | 5.8257 | 12.3185 | 12.2887 | C | 7.8030 | 15.5406 | 10.2711 | H | 2.6747 | -1.7447 | 19.4554 |
| C | 6.5061 | 13.4264 | 11.6674 | C | 2.4381 | 2.1575 | 19.2366 | C | 1.7602 | 0.0277 | 20.2303 |
| C | 6.3358 | 2.6825 | 18.7448 | H | 2.3530 | 3.2384 | 19.1948 | H | 1.1503 | -0.5011 | 20.9547 |
| C | 7.0879 | 14.5063 | 12.3476 | C | 1.6760 | 1.4362 | 20.1489 | B | 4.3160 | 2.0568 | 17.2169 |

1. Atomic coordinates for optimized structure of **COU-BDP-BF** in its S_2_ singlet excited electronic state

|  | x | y | z |  | x | y | z |  | x | y | z |
| --- | --- | --- | --- | --- | --- | --- | --- | --- | --- | --- | --- |
| O | 5.8917 | 12.4322 | 9.6005 | H | 7.1637 | 14.5428 | 13.4231 | H | 0.8417 | 2.0139 | 20.6374 |
| O | 4.1099 | 10.9470 | 14.8738 | C | 7.1531 | 3.6639 | 18.9706 | C | 6.2889 | -0.9820 | 15.5604 |
| O | 4.7472 | 10.5420 | 9.4926 | H | 7.9746 | 3.6779 | 19.6740 | H | 7.0587 | -1.1995 | 14.8262 |
| O | 6.1738 | 10.0891 | 14.5347 | C | 2.6528 | 5.8325 | 14.1231 | C | 7.6779 | 5.9033 | 17.8428 |
| N | 3.6361 | 2.8115 | 16.0527 | H | 2.8222 | 6.6635 | 14.8106 | H | 7.7741 | 6.1811 | 16.7911 |
| N | 5.3589 | 2.9935 | 17.8210 | H | 1.6191 | 5.8775 | 13.7705 | H | 8.6783 | 5.7352 | 18.2506 |
| C | 5.6993 | 4.2562 | 17.3423 | H | 3.3004 | 6.0085 | 13.2553 | H | 7.2561 | 6.7744 | 18.3587 |
| C | 3.8811 | 4.1325 | 15.6961 | C | 4.9318 | 0.6027 | 16.7357 | C | 6.1943 | 1.3867 | 19.6197 |
| N | 8.3425 | 16.4830 | 9.4898 | C | 5.2388 | 11.0672 | 14.4937 | H | 5.2387 | 1.2653 | 20.1381 |
| C | 5.2379 | 11.3630 | 11.6536 | C | 4.3113 | -0.4391 | 17.4403 | H | 6.9887 | 1.4108 | 20.3683 |
| H | 4.7110 | 10.5289 | 12.1005 | C | 5.9889 | 7.7603 | 14.1165 | H | 6.3421 | 0.5011 | 18.9950 |
| C | 5.8168 | 8.8220 | 14.9907 | H | 6.3505 | 7.9501 | 13.1115 | C | 3.2386 | 1.4777 | 18.3164 |
| C | 2.1123 | 3.3587 | 14.5137 | C | 5.7054 | 6.4728 | 14.5487 | C | 7.7400 | 15.5043 | 11.6251 |
| H | 1.2793 | 3.3014 | 13.8266 | H | 5.8476 | 5.6357 | 13.8730 | H | 8.2165 | 16.3133 | 12.1625 |
| C | 5.3771 | 8.6174 | 16.2902 | C | 2.0170 | 0.9554 | 15.3704 | C | 5.6755 | -2.0211 | 16.2526 |
| H | 5.2355 | 9.4598 | 16.9570 | H | 2.7773 | 0.2047 | 15.1357 | H | 5.9719 | -3.0467 | 16.0565 |
| C | 6.8479 | 4.6814 | 18.0301 | H | 1.2092 | 0.8638 | 14.6416 | C | 3.3124 | 0.0785 | 18.3776 |
| C | 5.2450 | 11.3702 | 10.2063 | H | 1.6139 | 0.7140 | 16.3582 | C | 8.9175 | 17.6222 | 10.1667 |
| C | 8.2482 | 16.4866 | 8.0483 | C | 5.2538 | 6.2274 | 15.8520 | H | 9.3696 | 18.2831 | 9.4282 |
| H | 8.6723 | 15.5718 | 7.6191 | C | 5.9171 | 0.3441 | 15.8022 | H | 9.7052 | 17.3183 | 10.8654 |
| H | 8.8136 | 17.3307 | 7.6552 | H | 6.3977 | 1.1518 | 15.2583 | H | 8.1662 | 18.1984 | 10.7236 |
| H | 7.2101 | 16.5788 | 7.7014 | C | 6.4902 | 13.4199 | 10.3084 | C | 5.8293 | 12.3179 | 13.8959 |
| C | 7.0879 | 14.4234 | 9.5659 | C | 4.6853 | -1.7575 | 17.1952 | H | 6.8549 | 12.4315 | 14.2605 |
| H | 7.0311 | 14.3475 | 8.4887 | H | 4.2146 | -2.5749 | 17.7343 | H | 5.2409 | 13.1658 | 14.2590 |
| C | 4.9447 | 4.8622 | 16.3025 | C | 5.1004 | 7.3250 | 16.7104 | C | 2.9115 | 4.5106 | 14.7552 |
| C | 2.5578 | 2.3360 | 15.3052 | H | 4.7378 | 7.1563 | 17.7193 | C | 2.4915 | -0.6195 | 19.2589 |
| C | 5.8381 | 12.3282 | 12.3888 | C | 7.7354 | 15.4905 | 10.2082 | H | 2.5400 | -1.7031 | 19.3194 |
| C | 6.4964 | 13.4159 | 11.7119 | C | 2.3631 | 2.1804 | 19.1221 | C | 1.6060 | 0.0862 | 20.0691 |
| C | 6.2461 | 2.6451 | 18.8332 | H | 2.3059 | 3.2637 | 19.0735 | H | 0.9626 | -0.4514 | 20.7584 |
| C | 7.1390 | 14.4934 | 12.3397 | C | 1.5390 | 1.4743 | 20.0038 | B | 4.3188 | 2.1006 | 17.1914 |

1. Atomic coordinates for optimized structure of **COU-BDP-BF** in its S_3_ singlet excited electronic state

|  | x | y | z |  | x | y | z |  | x | y | z |
| --- | --- | --- | --- | --- | --- | --- | --- | --- | --- | --- | --- |
| O | 6.0471 | 12.4630 | 9.5134 | H | 7.0444 | 14.5422 | 13.4322 | H | 1.0092 | 1.9567 | 20.8266 |
| O | 3.9656 | 10.8880 | 14.6462 | C | 7.2271 | 3.7314 | 18.8470 | C | 6.1520 | -1.0814 | 15.5604 |
| O | 4.9420 | 10.5576 | 9.3111 | H | 8.0714 | 3.7688 | 19.5230 | H | 6.8760 | -1.3480 | 14.7962 |
| O | 6.0657 | 10.0716 | 14.4584 | C | 2.6132 | 5.7872 | 14.0699 | C | 7.6226 | 5.9972 | 17.7261 |
| N | 3.5726 | 2.7889 | 16.0856 | H | 2.7137 | 6.6388 | 14.7469 | H | 7.7481 | 6.2465 | 16.6696 |
| N | 5.4108 | 2.9853 | 17.7751 | H | 1.6026 | 5.8075 | 13.6523 | H | 8.6177 | 5.8946 | 18.1686 |
| C | 5.7007 | 4.2548 | 17.2745 | H | 3.3105 | 5.9610 | 13.2424 | H | 7.1433 | 6.8633 | 18.1970 |
| C | 3.8206 | 4.1066 | 15.6992 | C | 4.9009 | 0.5991 | 16.7433 | C | 6.3773 | 1.4151 | 19.5244 |
| N | 8.4371 | 16.5515 | 9.6036 | C | 5.1172 | 11.0271 | 14.3484 | H | 5.4558 | 1.2431 | 20.0907 |
| C | 5.2878 | 11.3544 | 11.5097 | C | 4.3104 | -0.4570 | 17.5137 | H | 7.1972 | 1.4731 | 20.2435 |
| H | 4.7504 | 10.5053 | 11.9140 | C | 5.9564 | 7.7306 | 14.0688 | H | 6.5515 | 0.5309 | 18.9018 |
| C | 5.7115 | 8.8004 | 14.9138 | H | 6.3614 | 7.9133 | 13.0792 | C | 3.3098 | 1.4962 | 18.3815 |
| C | 2.0289 | 3.3462 | 14.5652 | C | 5.6879 | 6.4427 | 14.5126 | C | 7.7154 | 15.5367 | 11.6853 |
| H | 1.1792 | 3.2765 | 13.8987 | H | 5.8823 | 5.5975 | 13.8605 | H | 8.1431 | 16.3463 | 12.2614 |
| C | 5.2105 | 8.6071 | 16.1916 | C | 1.9003 | 0.9672 | 15.4976 | C | 5.5637 | -2.1133 | 16.3262 |
| H | 5.0156 | 9.4566 | 16.8357 | H | 2.6222 | 0.1777 | 15.2631 | H | 5.8441 | -3.1435 | 16.1359 |
| C | 6.8571 | 4.7301 | 17.9277 | H | 1.0760 | 0.8782 | 14.7868 | C | 3.3836 | 0.0666 | 18.4707 |
| C | 5.3822 | 11.3821 | 10.0656 | H | 1.4957 | 0.7592 | 16.4940 | C | 8.9316 | 17.6995 | 10.3272 |
| C | 8.4152 | 16.5819 | 8.1597 | C | 5.1800 | 6.2077 | 15.7943 | H | 9.4277 | 18.3726 | 9.6289 |
| H | 8.8639 | 15.6763 | 7.7368 | C | 5.8233 | 0.2538 | 15.7670 | H | 9.6678 | 17.4081 | 11.0845 |
| H | 8.9982 | 17.4338 | 7.8114 | H | 6.2902 | 1.0259 | 15.1644 | H | 8.1273 | 18.2581 | 10.8256 |
| H | 7.3959 | 16.6777 | 7.7610 | C | 6.5847 | 13.4500 | 10.2690 | C | 5.7239 | 12.2919 | 13.7952 |
| C | 7.2103 | 14.4731 | 9.5772 | C | 4.6446 | -1.8120 | 17.3017 | H | 6.7235 | 12.4178 | 14.2223 |
| H | 7.2204 | 14.4108 | 8.4977 | H | 4.1888 | -2.6003 | 17.8917 | H | 5.1016 | 13.1278 | 14.1288 |
| C | 4.8965 | 4.8361 | 16.2625 | C | 4.9494 | 7.3135 | 16.6206 | C | 2.8414 | 4.4788 | 14.7541 |
| C | 2.4833 | 2.3324 | 15.3823 | H | 4.5482 | 7.1495 | 17.6155 | C | 2.6123 | -0.6640 | 19.4004 |
| C | 5.8243 | 12.3198 | 12.2921 | C | 7.8000 | 15.5409 | 10.2712 | H | 2.6794 | -1.7454 | 19.4568 |
| C | 6.5042 | 13.4274 | 11.6696 | C | 2.4453 | 2.1572 | 19.2412 | C | 1.7686 | 0.0273 | 20.2354 |
| C | 6.3421 | 2.6794 | 18.7387 | H | 2.3610 | 3.2382 | 19.2003 | H | 1.1603 | -0.5014 | 20.9611 |
| C | 7.0881 | 14.5067 | 12.3489 | C | 1.6852 | 1.4359 | 20.1551 | B | 4.3174 | 2.0563 | 17.2160 |

1. Atomic coordinates for optimized structure of **COU-BDP-BF** in its T_1_ triplet excited electronic state

|  | x | y | z |  | x | y | z |  | x | y | z |
| --- | --- | --- | --- | --- | --- | --- | --- | --- | --- | --- | --- |
| O | 6.6772 | 12.2164 | 9.5044 | H | 6.4772 | 14.6373 | 13.3503 | H | 1.5095 | 2.0404 | 21.2546 |
| O | 3.6241 | 10.6802 | 14.1527 | C | 7.3825 | 3.7491 | 18.6201 | C | 5.8680 | -1.0836 | 15.2640 |
| O | 5.8600 | 10.1801 | 9.2200 | H | 8.2670 | 3.7830 | 19.2413 | H | 6.4819 | -1.3367 | 14.4046 |
| O | 5.7956 | 10.1391 | 14.4708 | C | 2.5189 | 5.7849 | 14.1061 | C | 7.5497 | 6.0909 | 17.6251 |
| N | 3.4621 | 2.8444 | 16.2122 | H | 2.4254 | 6.6412 | 14.7805 | H | 7.8433 | 6.2716 | 16.5867 |
| N | 5.4994 | 2.9641 | 17.6329 | H | 1.6251 | 5.7454 | 13.4797 | H | 8.4449 | 6.1563 | 18.2480 |
| C | 5.7009 | 4.2313 | 17.1780 | H | 3.3776 | 5.9926 | 13.4606 | H | 6.8788 | 6.9087 | 17.9045 |
| C | 3.7164 | 4.1089 | 15.7948 | C | 4.8024 | 0.5833 | 16.6545 | C | 6.6411 | 1.3551 | 19.2213 |
| N | 8.5270 | 16.5663 | 9.7872 | C | 4.7857 | 10.9617 | 14.0939 | H | 5.7597 | 1.1531 | 19.8382 |
| C | 5.5757 | 11.1487 | 11.3564 | C | 4.2970 | -0.4539 | 17.4637 | H | 7.5251 | 1.3674 | 19.8612 |
| H | 5.0454 | 10.2671 | 11.6955 | C | 6.0615 | 7.7979 | 14.2106 | H | 6.7273 | 0.5247 | 18.5134 |
| C | 5.4983 | 8.8530 | 14.9095 | H | 6.6628 | 8.0009 | 13.3310 | C | 3.4516 | 1.5006 | 18.5146 |
| C | 1.8269 | 3.4155 | 14.7447 | C | 5.8434 | 6.4994 | 14.6547 | C | 7.4415 | 15.5976 | 11.7268 |
| H | 0.9407 | 3.3438 | 14.1294 | H | 6.2803 | 5.6671 | 14.1117 | H | 7.6322 | 16.4866 | 12.3129 |
| C | 4.7300 | 8.6362 | 16.0429 | C | 1.7087 | 1.0608 | 15.7845 | C | 5.3612 | -2.0969 | 16.0726 |
| H | 4.2954 | 9.4733 | 16.5762 | H | 2.4084 | 0.2671 | 15.5040 | H | 5.5821 | -3.1344 | 15.8403 |
| C | 6.9153 | 4.7567 | 17.8047 | H | 0.8052 | 0.9662 | 15.1799 | C | 3.4917 | 0.0931 | 18.5731 |
| C | 6.0151 | 11.0988 | 9.9783 | H | 1.4515 | 0.8970 | 16.8358 | C | 8.7409 | 17.7968 | 10.5132 |
| C | 8.8779 | 16.4951 | 8.3875 | C | 5.0686 | 6.2422 | 15.7876 | H | 9.3033 | 18.4865 | 9.8850 |
| H | 9.5290 | 15.6376 | 8.1823 | C | 5.5874 | 0.2528 | 15.5571 | H | 9.3238 | 17.6290 | 11.4260 |
| H | 9.4195 | 17.3980 | 8.1084 | H | 5.9902 | 1.0348 | 14.9166 | H | 7.7969 | 18.2842 | 10.7914 |
| H | 7.9921 | 16.4150 | 7.7435 | C | 6.9097 | 13.3066 | 10.2742 | C | 5.3466 | 12.2653 | 13.5900 |
| C | 7.5736 | 14.3539 | 9.6600 | C | 4.5720 | -1.7880 | 17.1786 | H | 6.1893 | 12.5530 | 14.2261 |
| H | 7.8523 | 14.2277 | 8.6228 | H | 4.1813 | -2.5863 | 17.8038 | H | 4.5642 | 13.0227 | 13.6944 |
| C | 4.8301 | 4.8517 | 16.2554 | C | 4.5208 | 7.3316 | 16.4705 | C | 2.6696 | 4.5006 | 14.8410 |
| C | 2.3028 | 2.3980 | 15.5789 | H | 3.9171 | 7.1500 | 17.3544 | C | 2.8301 | -0.6046 | 19.5794 |
| C | 5.8055 | 12.2174 | 12.1548 | C | 7.8620 | 15.5271 | 10.3752 | H | 2.8624 | -1.6899 | 19.6226 |
| C | 6.4963 | 13.3595 | 11.6147 | C | 2.7352 | 2.1895 | 19.4852 | C | 2.1174 | 0.1066 | 20.5425 |
| C | 6.5204 | 2.6503 | 18.5190 | H | 2.6885 | 3.2764 | 19.4630 | H | 1.5971 | -0.4272 | 21.3322 |
| C | 6.7838 | 14.5413 | 12.3141 | C | 2.0684 | 1.4970 | 20.4983 | B | 4.3139 | 2.0195 | 17.2378 |

1. Atomic coordinates for optimized structure of **COU-BDP-BF** in its T_2_ triplet excited electronic state

|  | x | y | z |  | x | y | z |  | x | y | z |
| --- | --- | --- | --- | --- | --- | --- | --- | --- | --- | --- | --- |
| O | 6.2602 | 12.3061 | 9.5314 | H | 6.8357 | 14.5904 | 13.4222 | H | 1.1667 | 2.0618 | 20.9378 |
| O | 3.8477 | 10.8266 | 14.5558 | C | 7.2571 | 3.6879 | 18.8212 | C | 6.0823 | -1.0008 | 15.3760 |
| O | 5.2657 | 10.3404 | 9.3267 | H | 8.1063 | 3.7161 | 19.4907 | H | 6.7739 | -1.2338 | 14.5722 |
| O | 5.9905 | 10.1069 | 14.5237 | C | 2.6058 | 5.7908 | 14.0695 | C | 7.5944 | 6.0184 | 17.7894 |
| N | 3.5438 | 2.8274 | 16.1336 | H | 2.6987 | 6.6579 | 14.7277 | H | 7.7488 | 6.2941 | 16.7432 |
| N | 5.4421 | 2.9741 | 17.7256 | H | 1.6090 | 5.8083 | 13.6216 | H | 8.5727 | 5.9481 | 18.2717 |
| C | 5.7103 | 4.2564 | 17.2675 | H | 3.3311 | 5.9344 | 13.2597 | H | 7.0559 | 6.8520 | 18.2556 |
| C | 3.7991 | 4.1377 | 15.7319 | C | 4.8732 | 0.6162 | 16.6744 | C | 6.4336 | 1.3408 | 19.4210 |
| N | 8.4386 | 16.5097 | 9.6315 | C | 5.0068 | 11.0179 | 14.3270 | H | 5.5161 | 1.1610 | 19.9884 |
| C | 5.3879 | 11.2457 | 11.5061 | C | 4.3130 | -0.4194 | 17.4400 | H | 7.2701 | 1.3631 | 20.1224 |
| H | 4.8559 | 10.3892 | 11.9016 | C | 6.0279 | 7.7642 | 14.1631 | H | 6.5745 | 0.4898 | 18.7485 |
| C | 5.6593 | 8.8299 | 14.9677 | H | 6.5101 | 7.9562 | 13.2107 | C | 3.3463 | 1.5133 | 18.4077 |
| C | 1.9845 | 3.3550 | 14.6231 | C | 5.7751 | 6.4690 | 14.5967 | C | 7.6009 | 15.5445 | 11.6923 |
| H | 1.1297 | 3.2878 | 13.9636 | H | 6.0623 | 5.6274 | 13.9747 | H | 7.9432 | 16.3967 | 12.2639 |
| C | 5.0527 | 8.6271 | 16.1977 | C | 1.8518 | 0.9899 | 15.5833 | C | 5.5298 | -2.0280 | 16.1347 |
| H | 4.7651 | 9.4729 | 16.8108 | H | 2.5725 | 0.2075 | 15.3300 | H | 5.7916 | -3.0588 | 15.9175 |
| C | 6.8676 | 4.7265 | 17.9358 | H | 0.9998 | 0.9009 | 14.9062 | C | 3.4107 | 0.1116 | 18.4667 |
| C | 5.6022 | 11.2179 | 10.0750 | H | 1.5006 | 0.7965 | 16.6006 | C | 8.8393 | 17.7006 | 10.3443 |
| C | 8.5516 | 16.4717 | 8.1917 | C | 5.1615 | 6.2308 | 15.8278 | H | 9.3502 | 18.3719 | 9.6551 |
| H | 9.0913 | 15.5784 | 7.8578 | C | 5.7518 | 0.3300 | 15.6445 | H | 9.5366 | 17.4664 | 11.1566 |
| H | 9.1113 | 17.3432 | 7.8541 | H | 6.1873 | 1.1266 | 15.0483 | H | 7.9819 | 18.2390 | 10.7701 |
| H | 7.5700 | 16.4835 | 7.6991 | C | 6.6872 | 13.3491 | 10.2830 | C | 5.5904 | 12.2926 | 13.7760 |
| C | 7.3200 | 14.3723 | 9.5990 | C | 4.6426 | -1.7443 | 17.1697 | H | 6.5431 | 12.4879 | 14.2780 |
| H | 7.4230 | 14.2666 | 8.5277 | H | 4.2140 | -2.5526 | 17.7555 | H | 4.9001 | 13.1044 | 14.0234 |
| C | 4.8861 | 4.8480 | 16.2842 | C | 4.8089 | 7.3270 | 16.6188 | C | 2.8141 | 4.5027 | 14.7867 |
| C | 2.4284 | 2.3510 | 15.4378 | H | 4.3264 | 7.1549 | 17.5756 | C | 2.6683 | -0.5814 | 19.4184 |
| C | 5.8147 | 12.2667 | 12.2857 | C | 7.7998 | 15.4961 | 10.2900 | H | 2.7109 | -1.6655 | 19.4725 |
| C | 6.4928 | 13.3795 | 11.6727 | C | 2.5414 | 2.2161 | 19.2866 | C | 1.8631 | 0.1296 | 20.3044 |
| C | 6.3945 | 2.6324 | 18.6897 | H | 2.4848 | 3.2998 | 19.2426 | H | 1.2808 | -0.4039 | 21.0491 |
| C | 6.9693 | 14.5129 | 12.3484 | C | 1.7978 | 1.5180 | 20.2417 | B | 4.3286 | 2.0963 | 17.1990 |

1. Atomic coordinates for optimized structure of **COU-BDP-BF** in its T_3_ triplet excited electronic state

|  | x | y | z |  | x | y | z |  | x | y | z |
| --- | --- | --- | --- | --- | --- | --- | --- | --- | --- | --- | --- |
| O | 6.1134 | 12.4247 | 9.5084 | H | 6.9890 | 14.5535 | 13.4300 | H | 1.0533 | 1.9672 | 20.8659 |
| O | 3.9272 | 10.8709 | 14.6067 | C | 7.2233 | 3.7307 | 18.8430 | C | 6.1440 | -1.0561 | 15.5212 |
| O | 5.0363 | 10.5041 | 9.2979 | H | 8.0679 | 3.7692 | 19.5186 | H | 6.8609 | -1.3167 | 14.7484 |
| O | 6.0402 | 10.0786 | 14.4673 | C | 2.6072 | 5.7850 | 14.0648 | C | 7.6015 | 6.0127 | 17.7393 |
| N | 3.5556 | 2.7938 | 16.0985 | H | 2.7046 | 6.6418 | 14.7356 | H | 7.7289 | 6.2721 | 16.6855 |
| N | 5.4143 | 2.9814 | 17.7668 | H | 1.5998 | 5.8036 | 13.6397 | H | 8.5954 | 5.9170 | 18.1859 |
| C | 5.6955 | 4.2594 | 17.2717 | H | 3.3110 | 5.9506 | 13.2410 | H | 7.1103 | 6.8695 | 18.2152 |
| C | 3.8074 | 4.1129 | 15.7063 | C | 4.8963 | 0.6092 | 16.7299 | C | 6.3906 | 1.4045 | 19.5048 |
| N | 8.4514 | 16.5425 | 9.6170 | C | 5.0827 | 11.0229 | 14.3316 | H | 5.4722 | 1.2265 | 20.0738 |
| C | 5.3175 | 11.3267 | 11.4960 | C | 4.3134 | -0.4494 | 17.4976 | H | 7.2154 | 1.4597 | 20.2184 |
| H | 4.7798 | 10.4753 | 11.8951 | C | 5.9657 | 7.7358 | 14.0855 | H | 6.5608 | 0.5273 | 18.8719 |
| C | 5.6901 | 8.8062 | 14.9202 | H | 6.3906 | 7.9191 | 13.1045 | C | 3.3228 | 1.5001 | 18.3907 |
| C | 2.0144 | 3.3458 | 14.5773 | C | 5.7004 | 6.4467 | 14.5285 | C | 7.6911 | 15.5390 | 11.6904 |
| H | 1.1639 | 3.2745 | 13.9119 | H | 5.9180 | 5.6010 | 13.8843 | H | 8.0949 | 16.3592 | 12.2687 |
| C | 5.1617 | 8.6128 | 16.1870 | C | 1.8821 | 0.9719 | 15.5228 | C | 5.5603 | -2.0904 | 16.2840 |
| H | 4.9437 | 9.4628 | 16.8230 | H | 2.6028 | 0.1817 | 15.2877 | H | 5.8385 | -3.1200 | 16.0852 |
| C | 6.8466 | 4.7381 | 17.9270 | H | 1.0520 | 0.8817 | 14.8192 | C | 3.3930 | 0.0724 | 18.4709 |
| C | 5.4477 | 11.3414 | 10.0545 | H | 1.4884 | 0.7704 | 16.5247 | C | 8.9161 | 17.7025 | 10.3414 |
| C | 8.4667 | 16.5578 | 8.1727 | C | 5.1656 | 6.2129 | 15.7986 | H | 9.4207 | 18.3750 | 9.6485 |
| H | 8.9380 | 15.6539 | 7.7711 | C | 5.8161 | 0.2746 | 15.7432 | H | 9.6378 | 17.4264 | 11.1182 |
| H | 9.0473 | 17.4138 | 7.8307 | H | 6.2768 | 1.0540 | 15.1445 | H | 8.0942 | 18.2562 | 10.8157 |
| H | 7.4569 | 16.6359 | 7.7471 | C | 6.6204 | 13.4255 | 10.2672 | C | 5.6859 | 12.2907 | 13.7823 |
| C | 7.2508 | 14.4492 | 9.5810 | C | 4.6469 | -1.7979 | 17.2712 | H | 6.6728 | 12.4338 | 14.2330 |
| H | 7.2885 | 14.3767 | 8.5027 | H | 4.1986 | -2.5928 | 17.8584 | H | 5.0444 | 13.1210 | 14.0926 |
| C | 4.8850 | 4.8386 | 16.2662 | C | 4.9041 | 7.3181 | 16.6153 | C | 2.8306 | 4.4825 | 14.7600 |
| C | 2.4673 | 2.3346 | 15.3988 | H | 4.4820 | 7.1536 | 17.6014 | C | 2.6302 | -0.6547 | 19.4040 |
| C | 5.8234 | 12.3058 | 12.2818 | C | 7.8104 | 15.5307 | 10.2787 | H | 2.6917 | -1.7368 | 19.4571 |
| C | 6.5056 | 13.4153 | 11.6656 | C | 2.4734 | 2.1656 | 19.2666 | C | 1.7987 | 0.0391 | 20.2535 |
| C | 6.3458 | 2.6739 | 18.7287 | H | 2.3965 | 3.2476 | 19.2302 | H | 1.1970 | -0.4924 | 20.9832 |
| C | 7.0598 | 14.5081 | 12.3485 | C | 1.7197 | 1.4467 | 20.1846 | B | 4.3134 | 2.0544 | 17.2171 |

# Optical properties

FRET is a radiationless process of a dipole-dipole nature, the energy transfer efficiency is often used to measure the relative distance between the donor and acceptor as it is strongly distance dependent. The rate of energy transfer depends on (i) the spectral overlap (J($v$)) - the greater the overlap, the higher the efficiency is, (ii) the photoluminescent quantum yield of the donor, (iii) the separation distance (R) between the donor and acceptor molecules, and (iv) the relative orientation of the donor emission and the acceptor excitation dipoles.

$$J(v)= \int_{0}^{\infty} F_{D}(v)\varepsilon_{A}(v)\frac{dv}{v^{4}}$$

$$R_{0}^{6}=\frac{9000(ln(10)\kappa^{2}Q_{D}}{128\pi^{5}Nn^{4}}J(v)$$

Where $\kappa^{2}$ is the mutual orientation of the dipoles in space, $R_{0}$ is the critical distance between donor and acceptor (at $R_{0}$ FRET efficiency is 50% which means that energy transfer and spontaneous deactivation of the donor are of equal probability), n is the refractive index of the solvent, $Q_{D}$ is the quantum yield of donor emission. $R_{0}$typically ranges from 50 to 100 Å.

# Photocatalytic activity and photostability studies

**
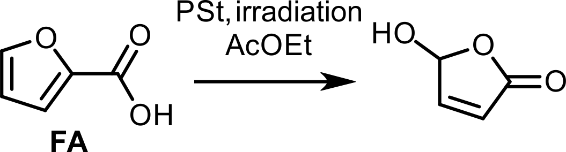
**

Scheme S1. Photocatalytic oxidation of 2-furoic acid.

**Photocatalytic reactions:** Catalytic reactions were performed using homemade photoreactor, which ensures stable and repeatable conditions and exchangeable light source. The reactor comprises of aluminium tube (Ø=150 mm) equipped with 26 W neutral-white light (CIE 1931 (0.38899; 0.37837)) LED stripe (54 diodes) or 365 nm LED stripes (26 W, 104 diodes) and plastic cover containing 8 holes for 4 mL vials allowing for parallelization of the experiment. The LED stripe was glued inside the tube. To the bottom of the aluminium tube three plastic legs were attached to ensure flow of air. The cover was equipped with a fan (Ø=60 mm) with air diffusor, while the aluminium tube was cooled by a copper coiled tube heat exchanger sticked to the outside wall of the reactor. Temperature inside reactor was controlled by placing Pt-100 thermometer into one of the reaction vials filled with AcOEt. Under such conditions the temperature inside the reactor was maintained at 25 °C. Vials containing 1.5 mL of AcOEt solutions of 2-furoic acid (12 mg ⋅ mL^−1^; 0.107 M) and studied photosensitizer (0.05 % mol with respect to substrate, 5.4×10^−5^ M) were placed in the photoreactor. The distance from the light source was the same for all samples (25 mm), providing the same irradiance of 1200 W⸱m^−2^. The reactor was placed on magnetic stirrer. Each vial was equipped with cross shaped stirrer bars to ensure vigorous stirring. Reaction progress was monitored by ^1^H NMR spectra analysis of the reaction mixture sampled after a given time. The control experiments show that reaction does not proceed neither in the absence of light nor photocatalyst.

**Electron Paramagnetic Resonance (EPR)**


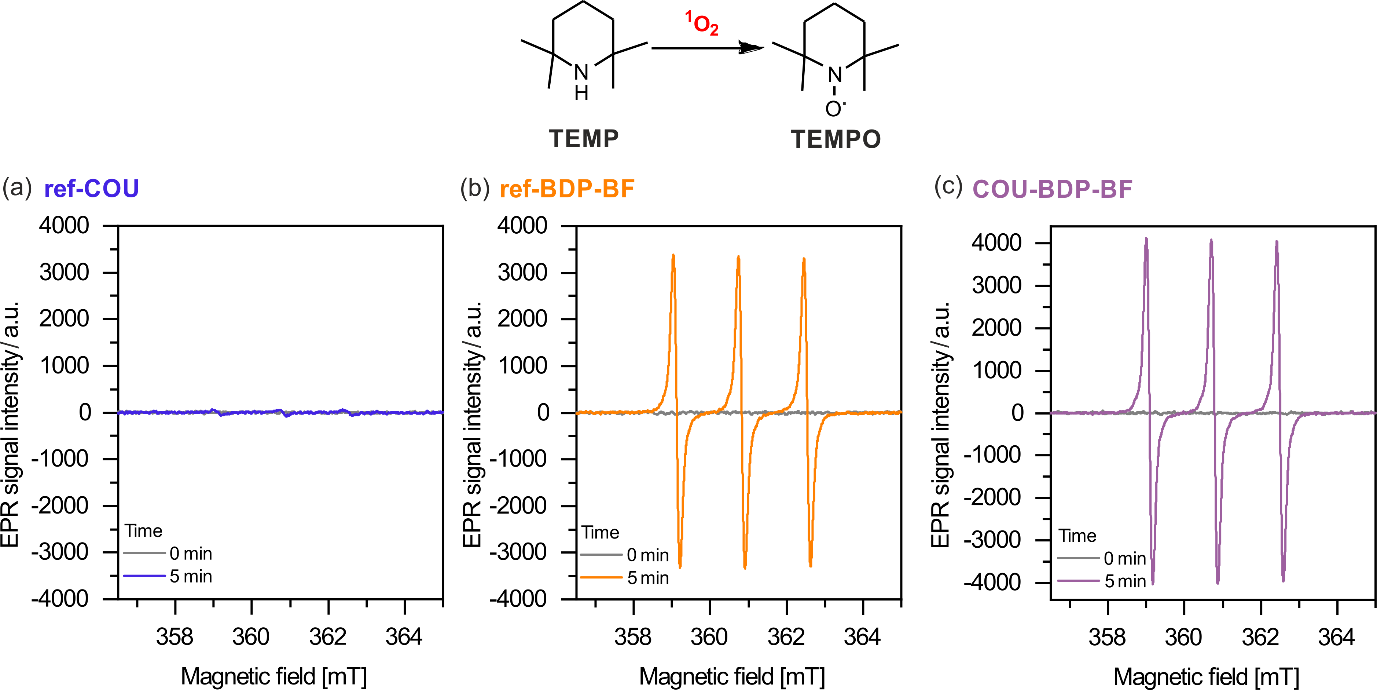


1. EPR spectra in DCM of TEMP spin-trapping adducts with ¹O_2_ produced by (a) **ref-COU**, (b) **ref-BDP-BF** and (c) **COU-BDP-BF** under irradiation.

**Singlet oxygen emission spectra** were recorded using Edinburgh Instruments FS5 spectrofluorimeter equipped with NIR-PMT detector. Measurements were performed for all analyzed compounds in AcOEt (*C* = 10^-4^ M) at room temperature using Suprasil quartz quvetes (10.00 mm). For better spectra resolution, each spectra was recorded trice consecutively, without replenishing measured solution with oxygen. **ref-COU** did not emit in 1250-1350 nm region.


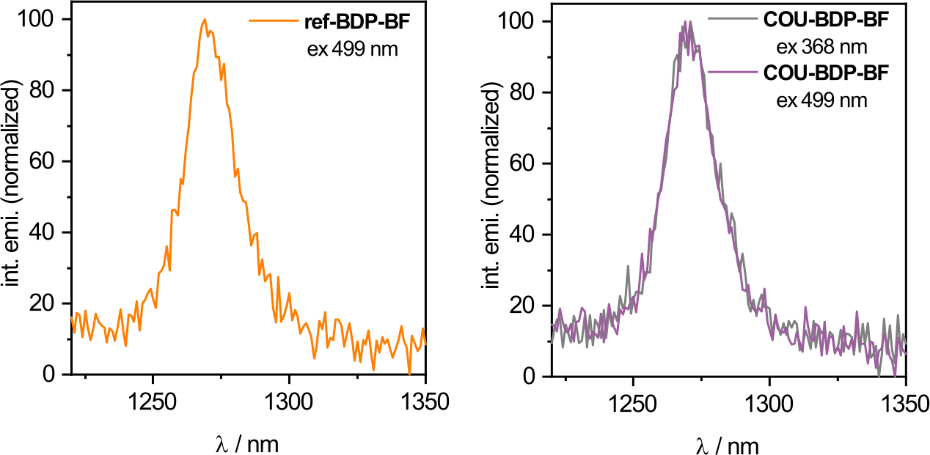


1. Emission of singlet oxygen generated by given photosensitizer excited with pointed wavelength.

**Photocatalytic stability** was determined with UV-Vis spectroscopy using a Hitachi U-2800 spectrophotometer. Experimental conditions were retained from photocatalytic test reactions. Concordantly, 4 mL vials containing 1.5 mL of AcOEt solutions of studied photosensitizer (c = 5.4×10^−5^ M, 1.5 mL) were placed in the photoreactor. Samples were irradiated with neutral-white light LED strips for 10 h straight. The decomposition process was monitored with UV-Vis spectroscopy with even 1 h time probing period (Figure S7). The parallel experiments were performed without light to determine hydrolytic stability. The half-time of pseudo-first order decomposition of **ref-BDP-BF** (15 h) and **COU-BDP-BF** (18.5 h) were estimated from the drop in absorption intensity at the absorption maximum wavelength (Figure S8).


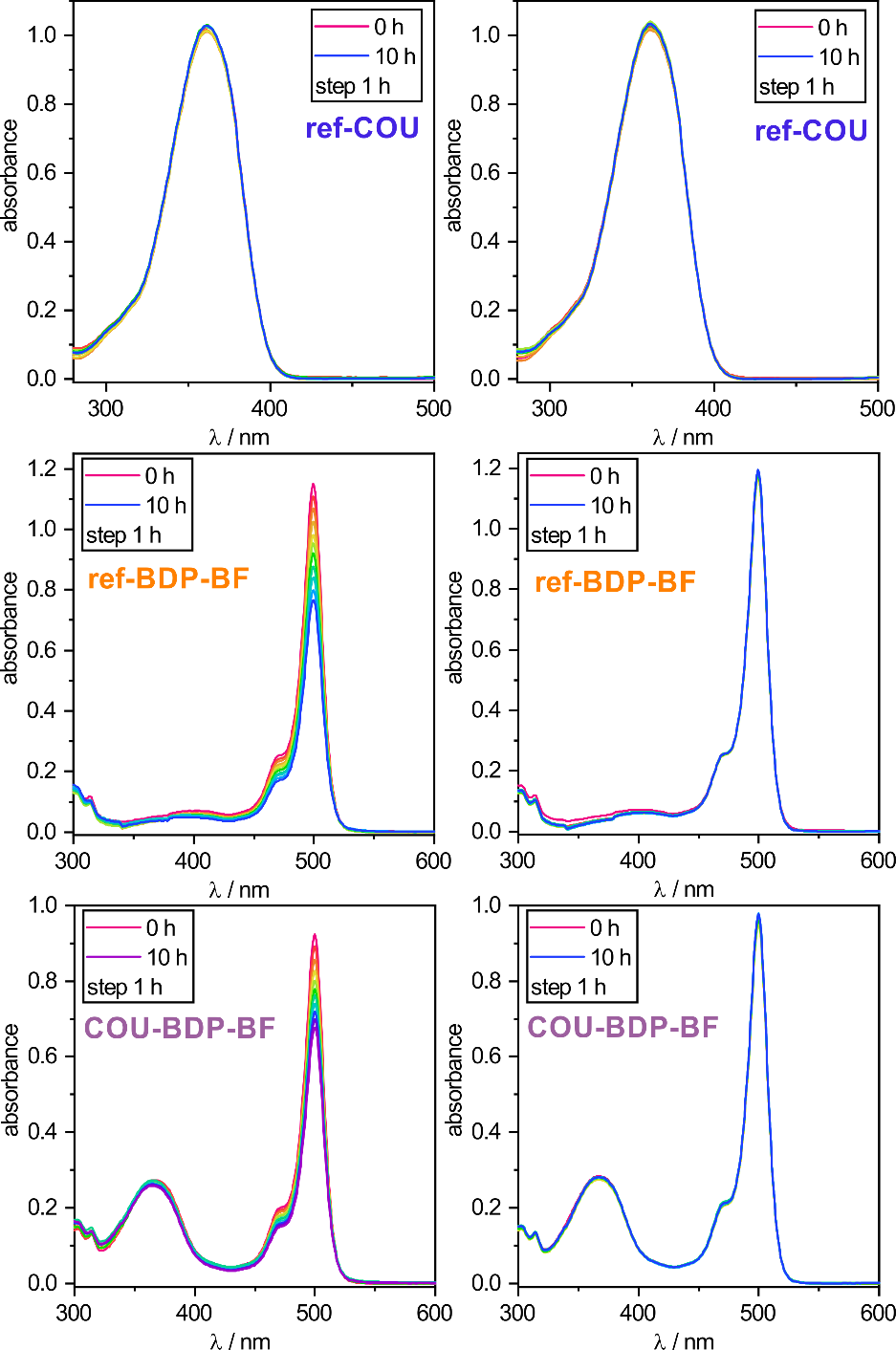


1. Overlay of absorption spectra of (a) **ref-COU-BF**, (b) **ref-BDP-BF** and (c) **COU-BDP-BF** in AcOEt upon irradiation (left) and in the darkroom (right).


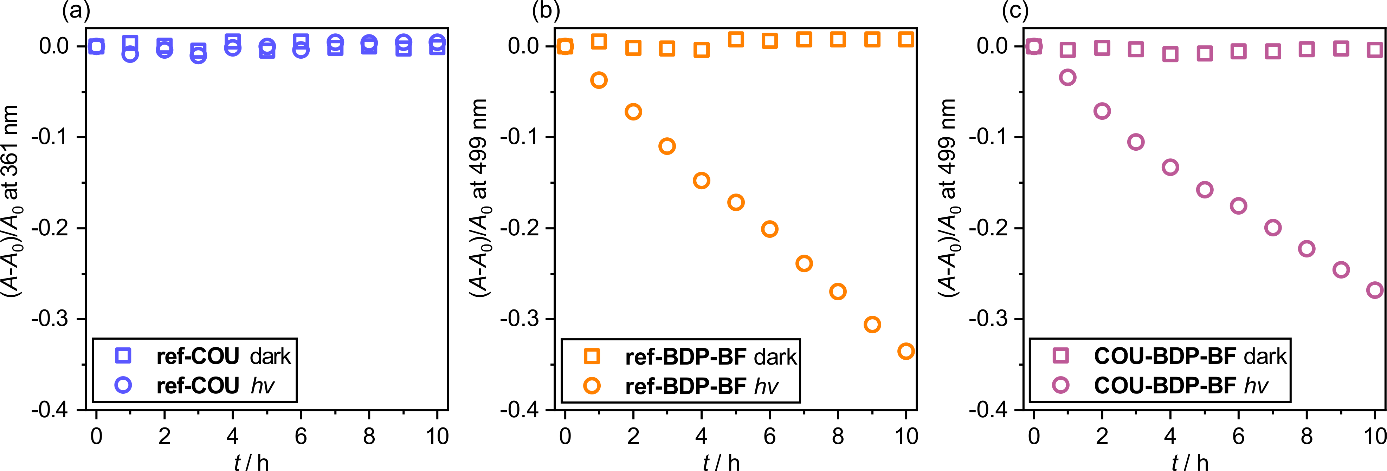


1. Drop in absorbance derived from photostability studies of (a) **ref-COU**, (b) **ref-BDP-BF** and (c) **COU-BDP-BF**.

# Microscopy


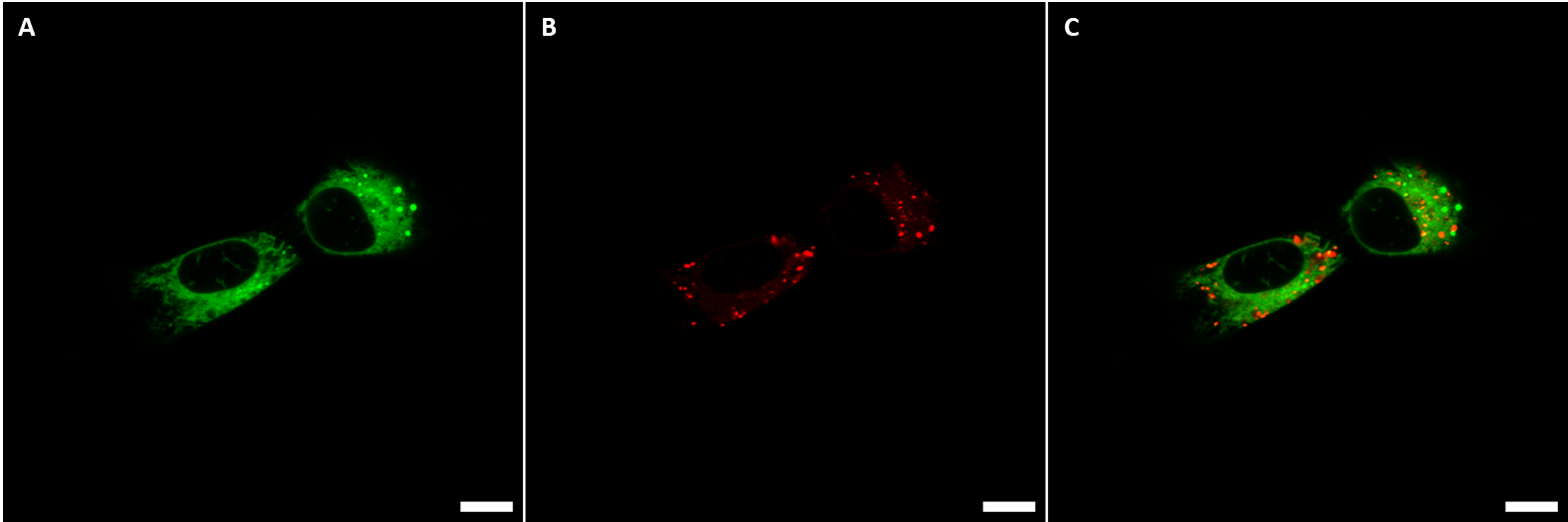


1. Localisation study of **COU-BDP-BF** with LysoTracker™ Red DND-99. 5 μM **COU-BDP-BF** solution incubated for 2 hours. A Image of **COU-BDP-BF** (λ_ex_ = 488 nm, λ_em_ = 500-540 nm, 3 mW). B Image of LysoTracker™ Red DND-99 (λ_ex_ = 543 nm, λ_em_ = 600-750 nm, 6 mW). C RGB merge of A and B. Scale bars = 10 μM.


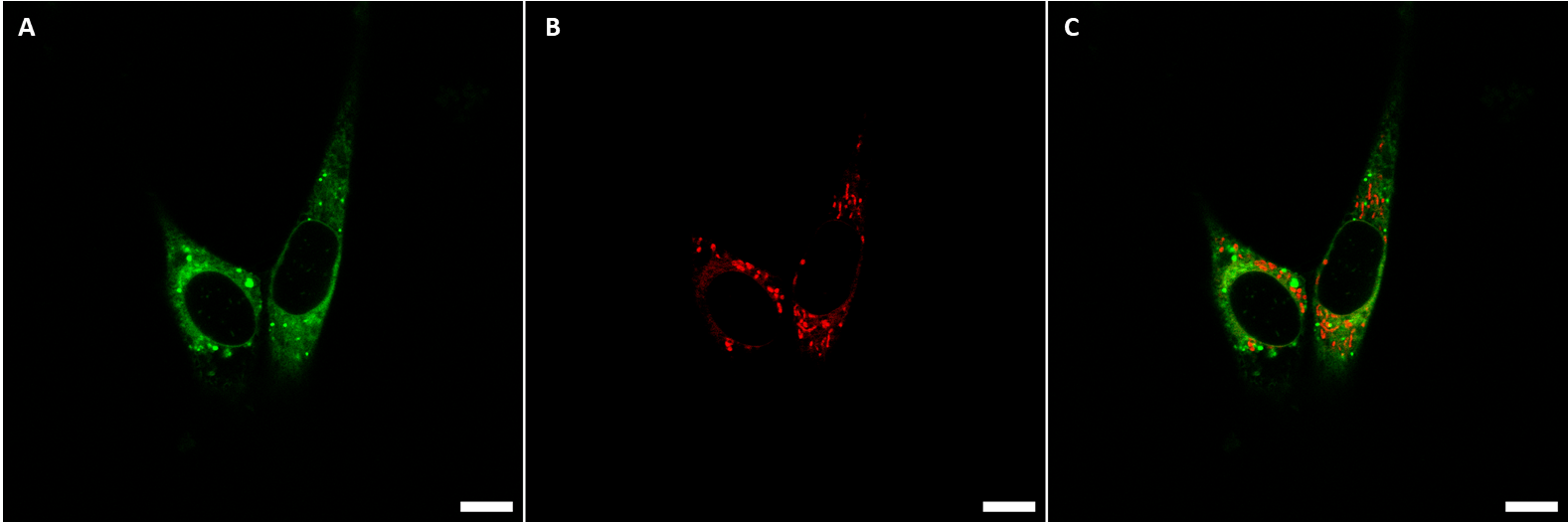


1. Localisation study of **COU-BDP-BF** with MitoTracker™ Red. 5 μM **COU-BDP-BF** solution incubated for 2 hours. A Image of **COU-BDP-BF** (λ_ex_ = 488 nm, λ_em_ = 500-540 nm, 3 mW). B Image of MitoTracker™ Red (λ_ex_ = 543 nm, λ_em_ = 600-750 nm, 6 mW). C RGB merge of A and B. Scale bars = 10 μM.


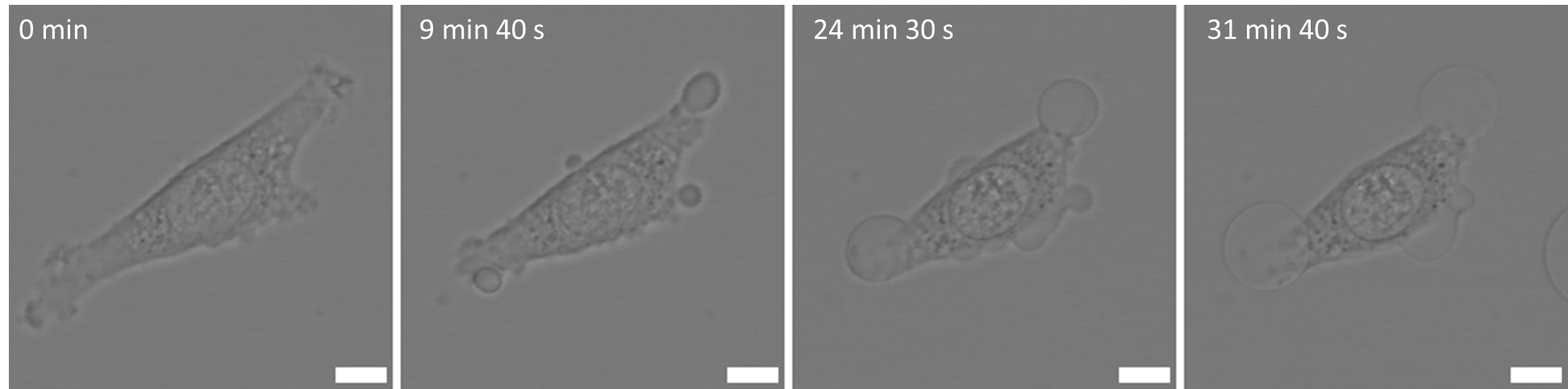


1. Microscopic observation of cell death caused by excitation at 488 nm (12 mW) (exposure times are shown for each image). Cells were incubated with 5 uM of **COU-BDP-BF** for 2h prior to the experiment. The images show blebbing and alteration to the cells’ overall morphology on the transmission images. Scale bars = 10 μm.

# Cytotoxicity Studies


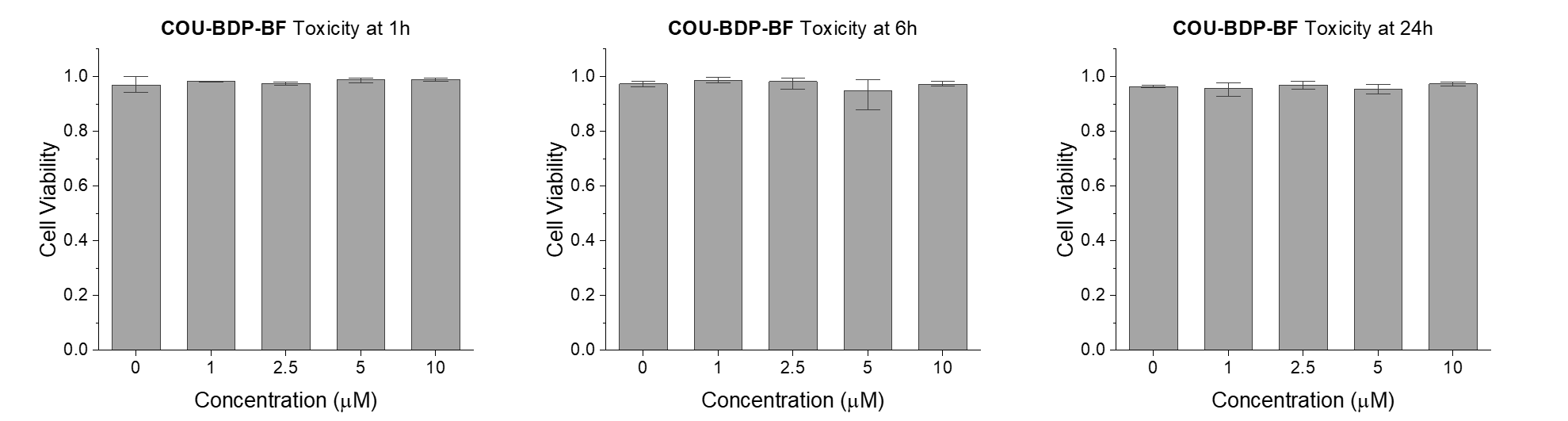


1. Cytotoxicity data for **COU-BDP-BF** after incubation at concentrations ranging from 0 to 10 μM at times 1 hour, 6 hours and 24 hours.

# NMR spectra and HRMS data

**
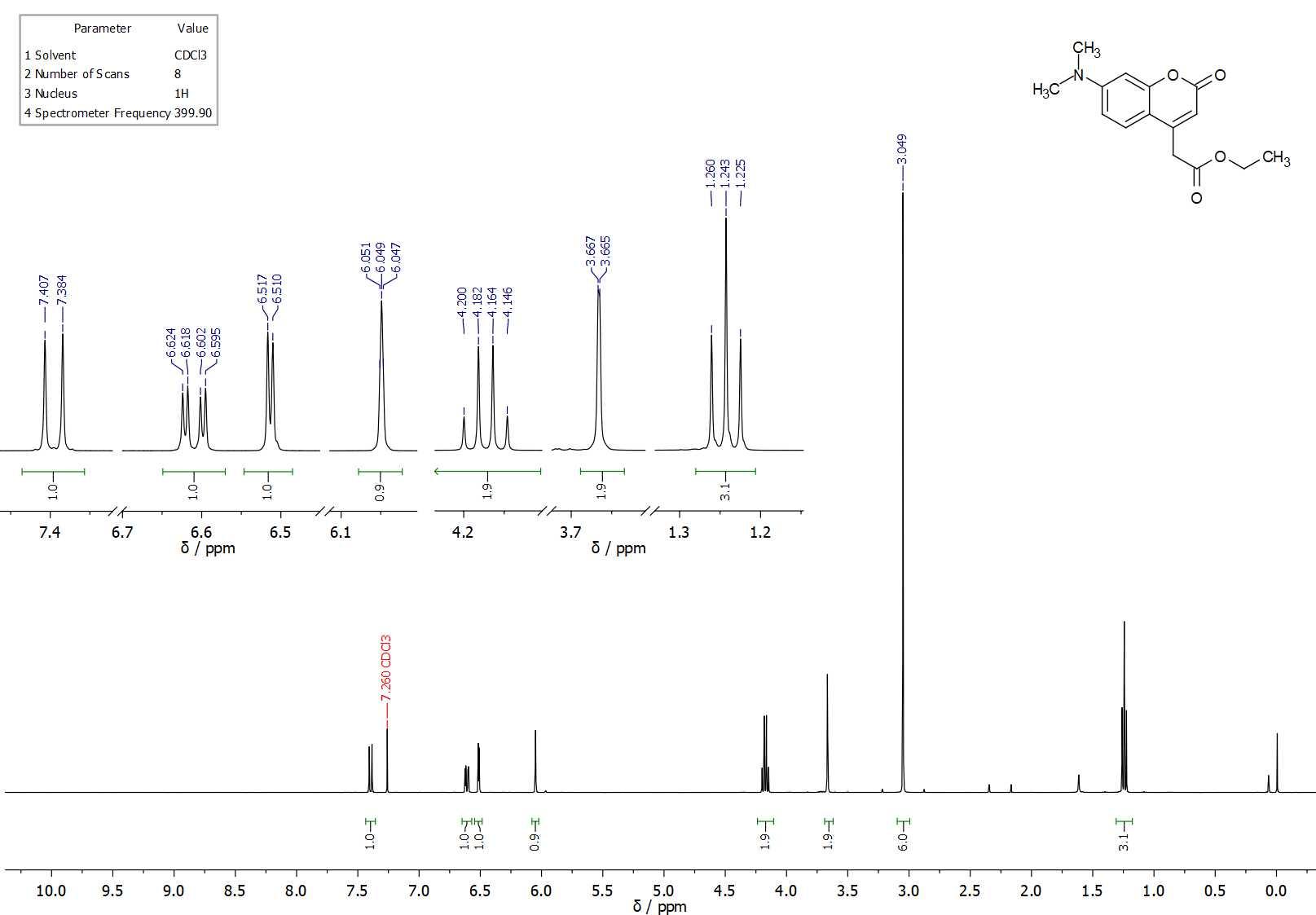
**

1. ^1^H NMR (400 MHz) spectra of **ref-COU** in CDCl_3_.

1. ^13^C{^1^H} NMR (151 MHz) spectra of **ref-COU** in CDCl_3_.

**
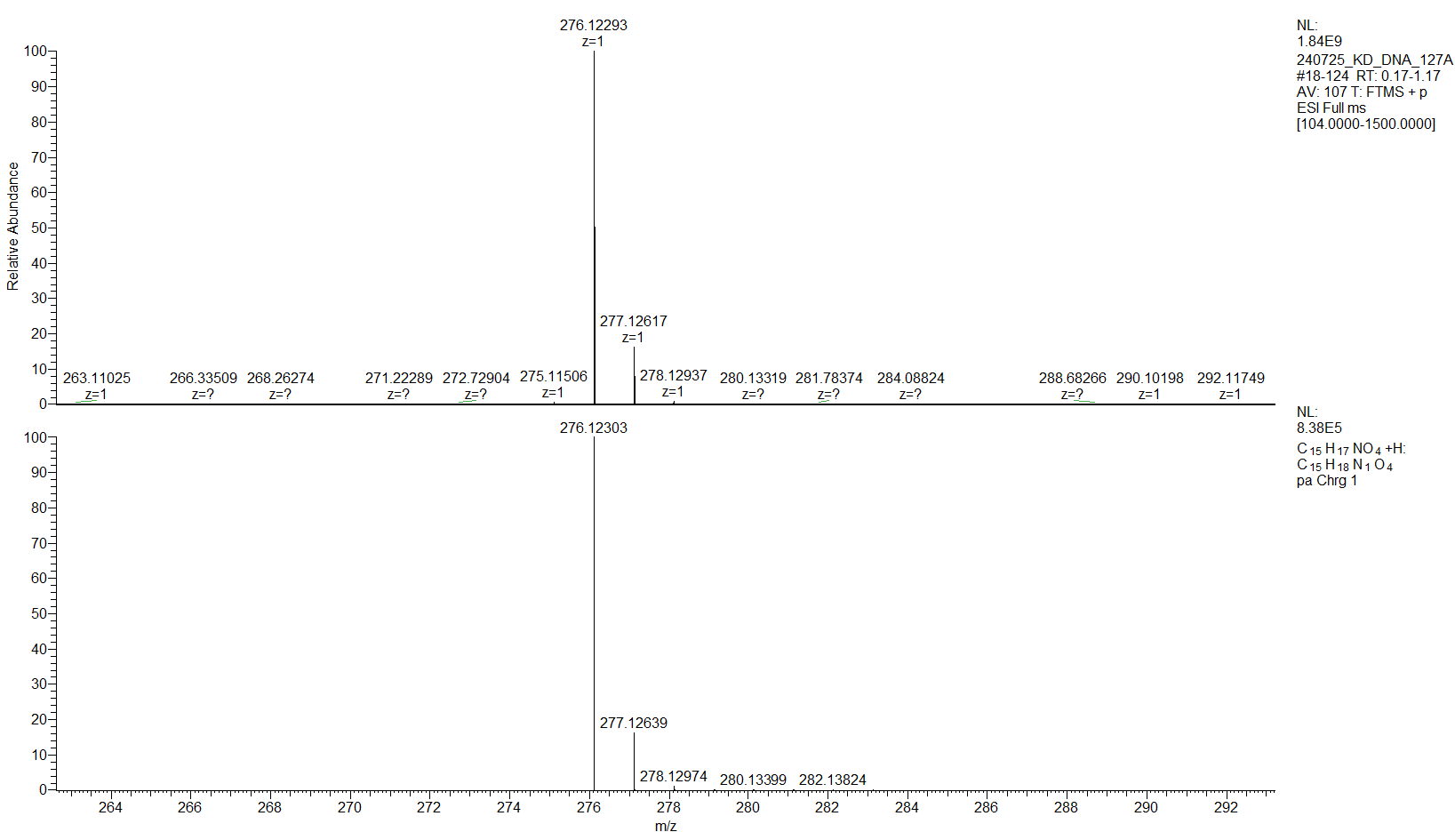
**

1. HRMS spectrum (ESI, positive ion mode) of **ref-COU**. The calculated spectrum of the formula C_15_H_17_NO_4_^+^ [MH^+^] is given in the bottom.


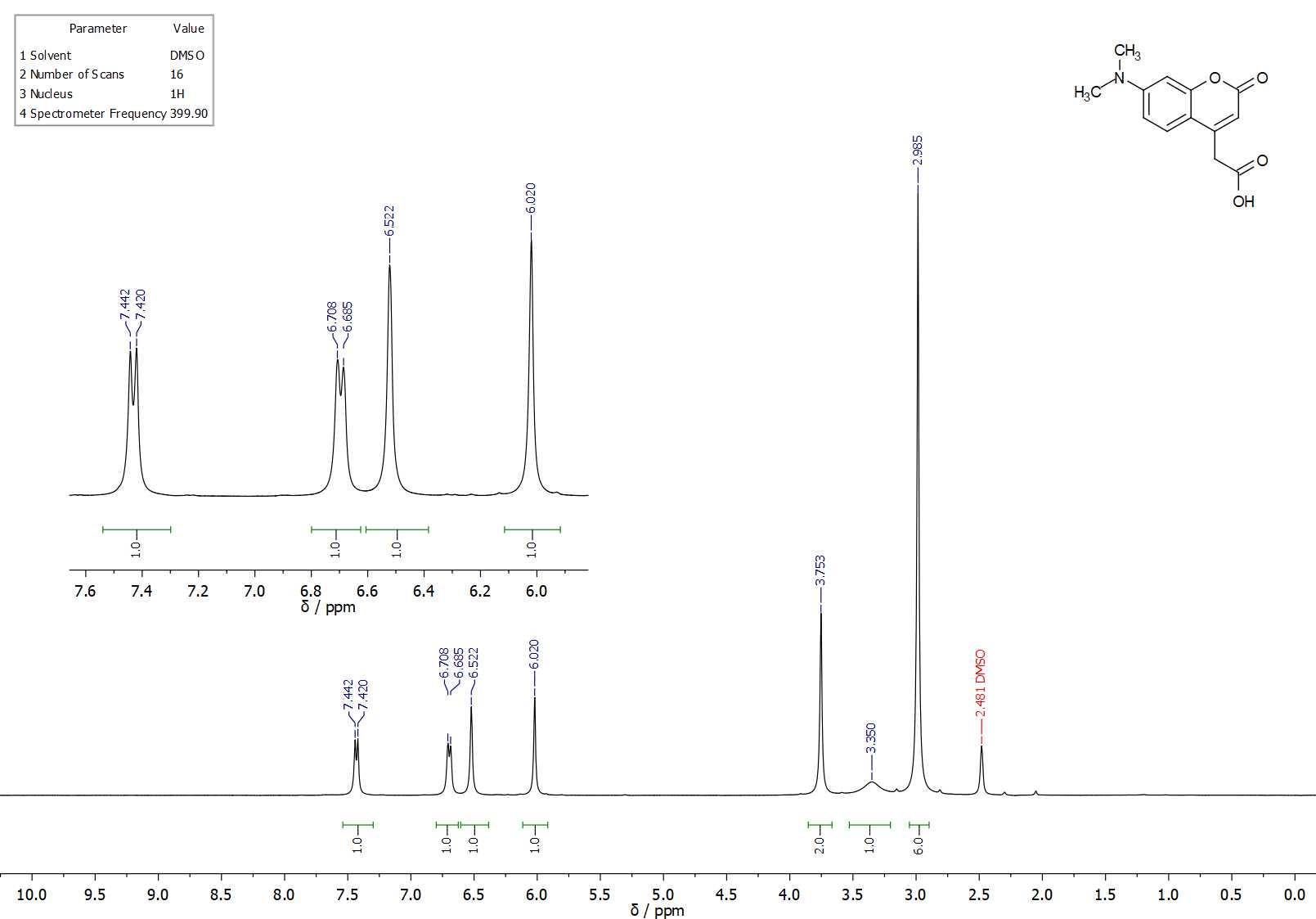


1. ^1^H NMR (400 MHz) spectra of compound **1** in DMSO-*d*6.

1. ^13^C{^1^H} NMR (151 MHz) spectra of compound **1** in DMSO-*d*6.


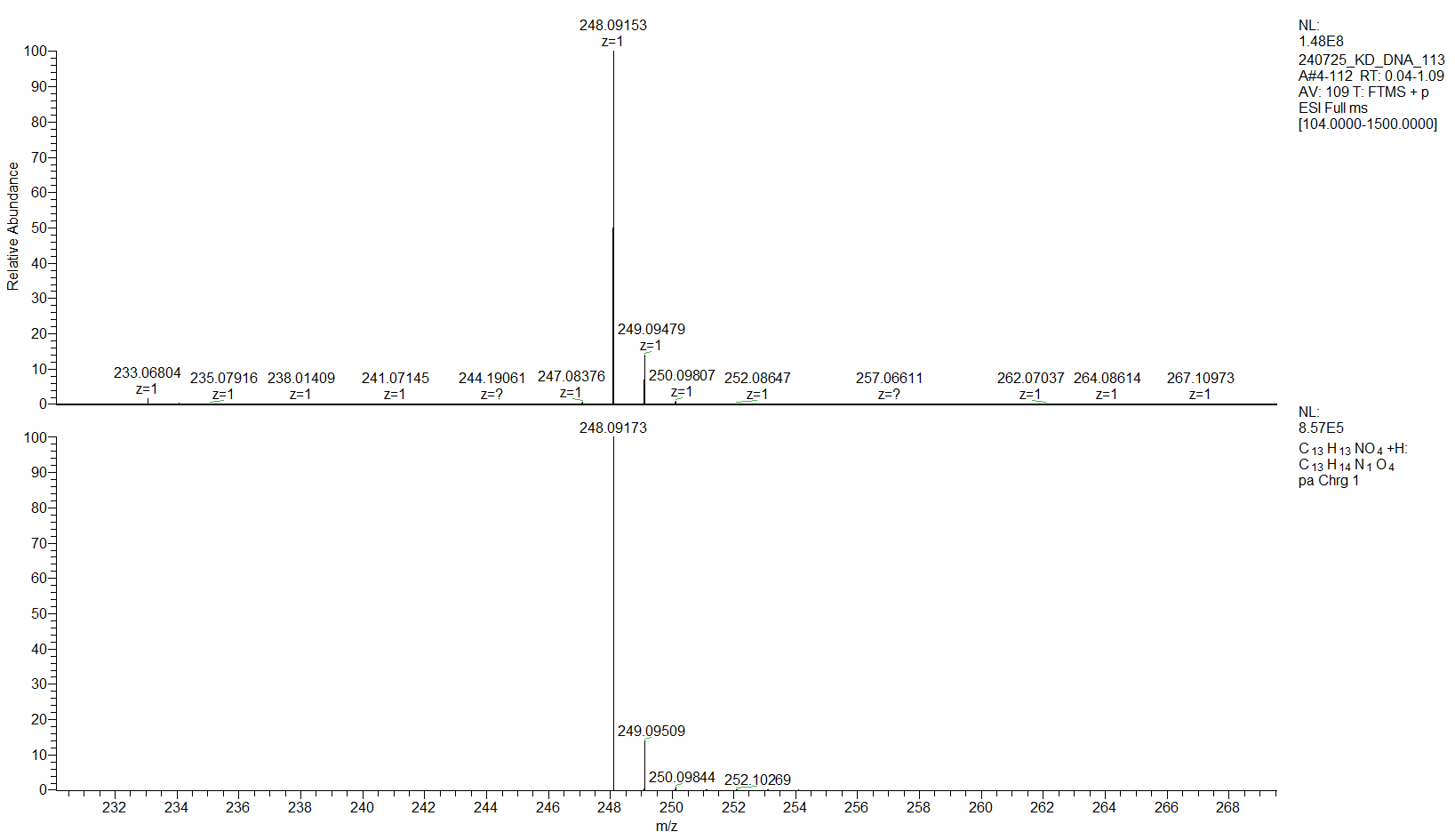


1. HRMS spectrum (ESI, positive ion mode) of compound **1**. The calculated spectrum of the formula C_13_H_13_NO_4_^+^ [MH^+^] is given in the bottom.


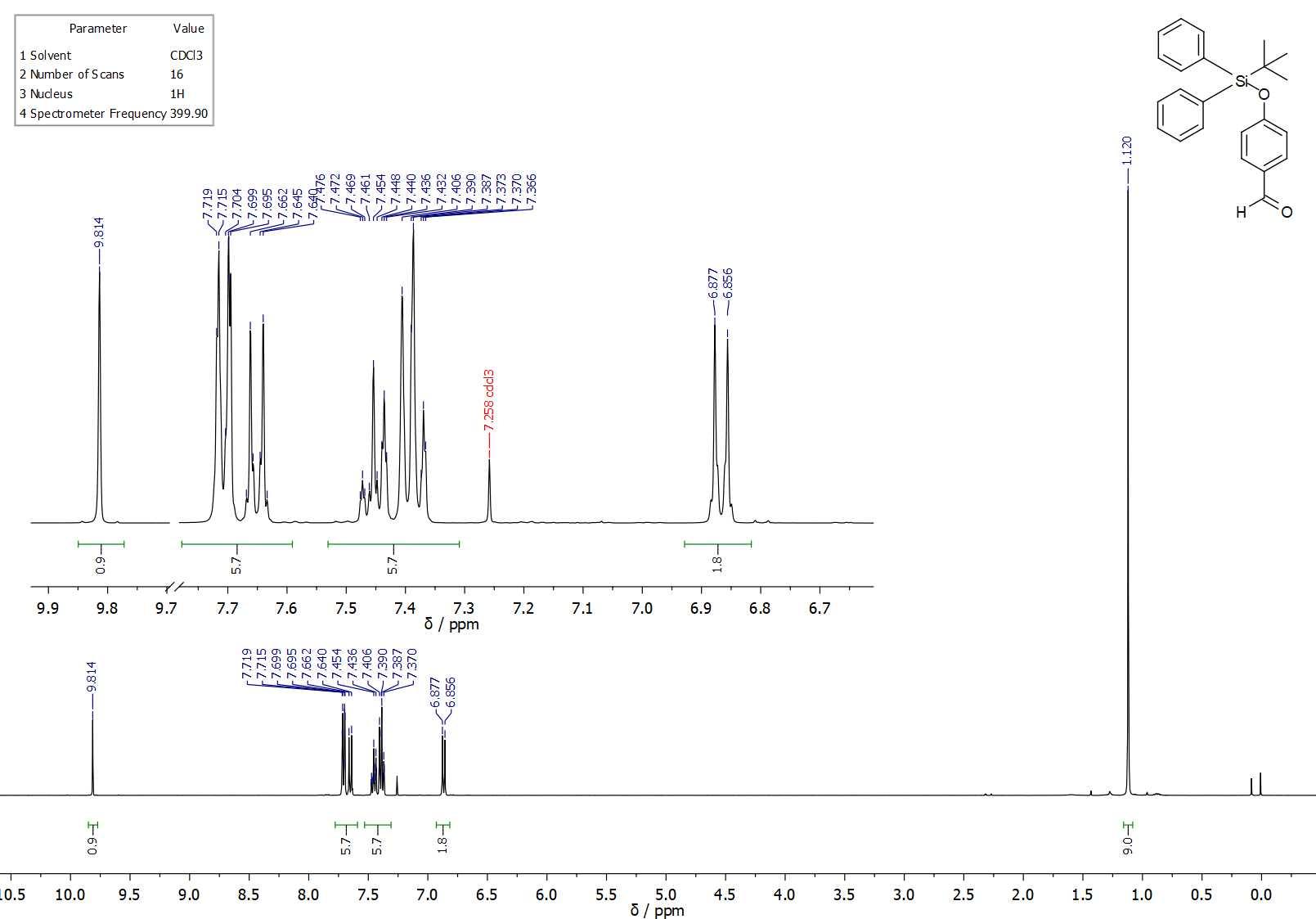


1. ^1^H NMR (400 MHz) spectra of 4-(*tert*-butyldiphenylsilyloxy)benzaldehyde in CDCl_3_.


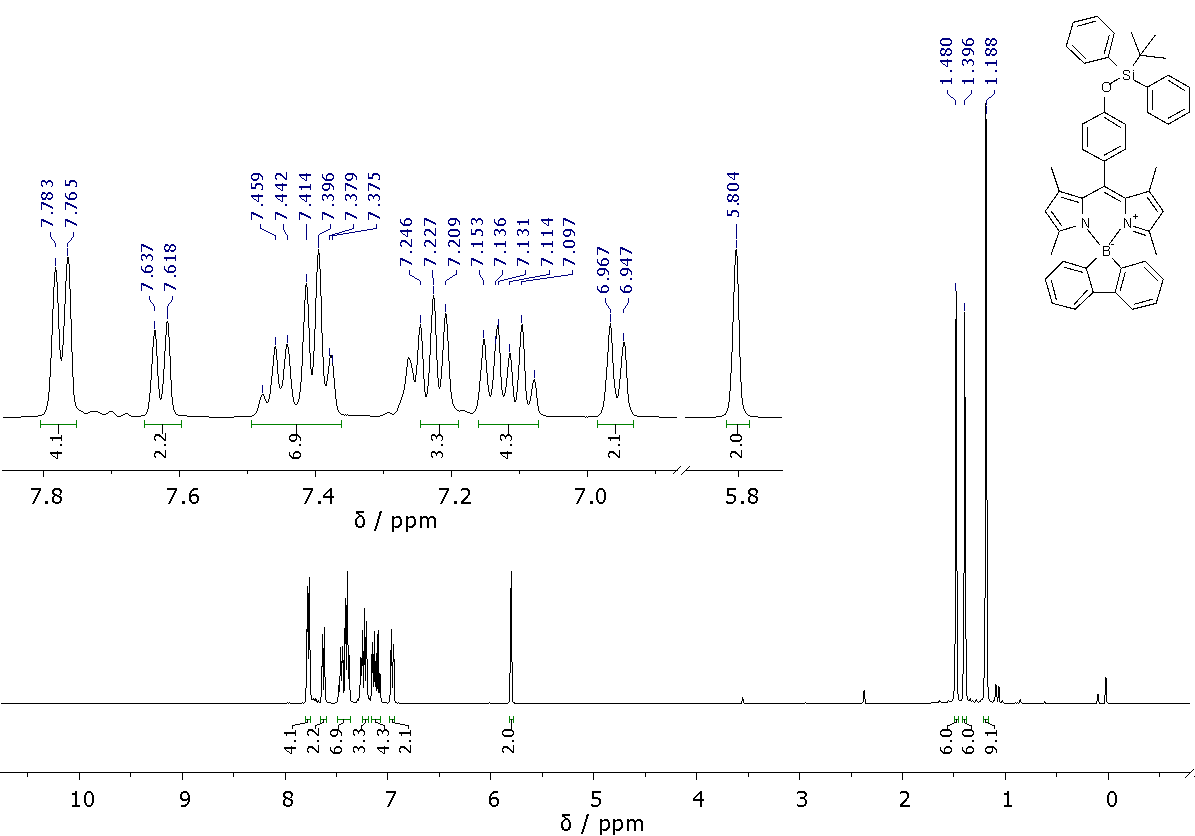


1. ^1^H NMR (300 MHz) spectra of **2** in CDCl_3_.


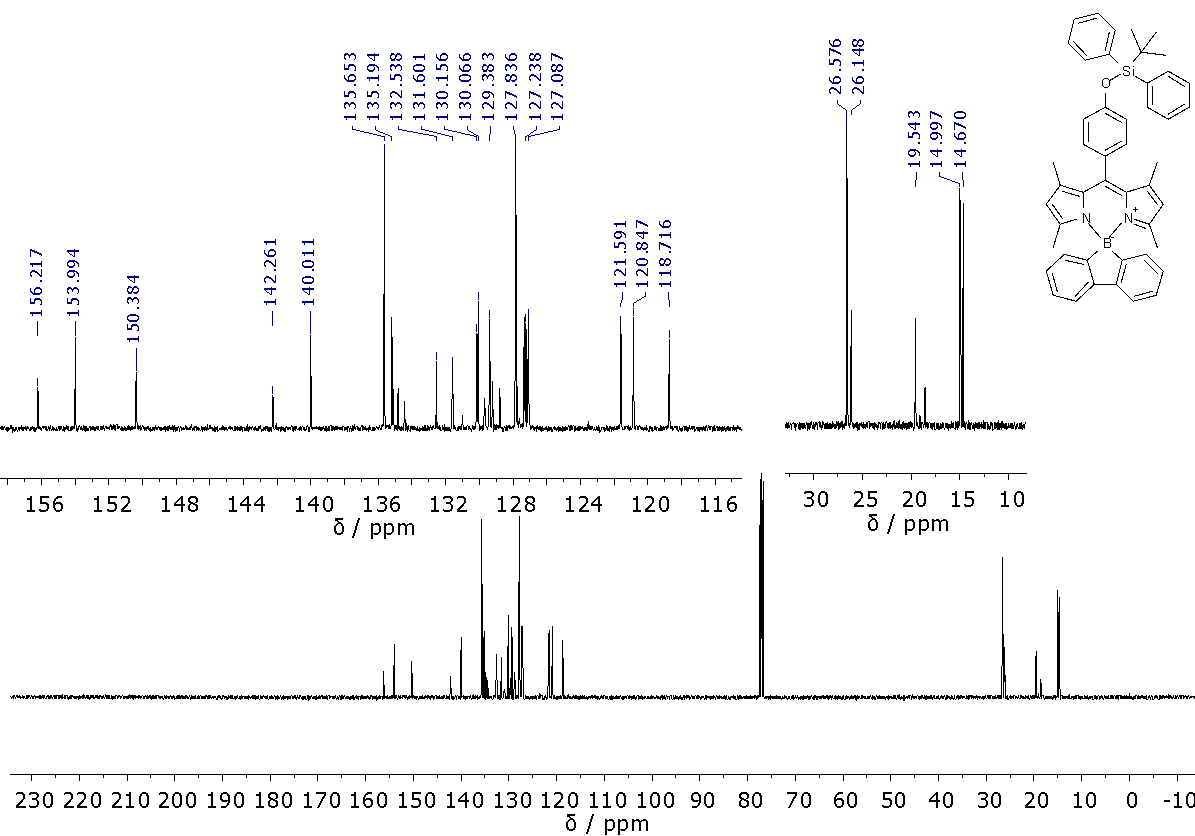


1. ^13^C{^1^H} NMR (101 MHz) spectra of **2** in CDCl_3_.


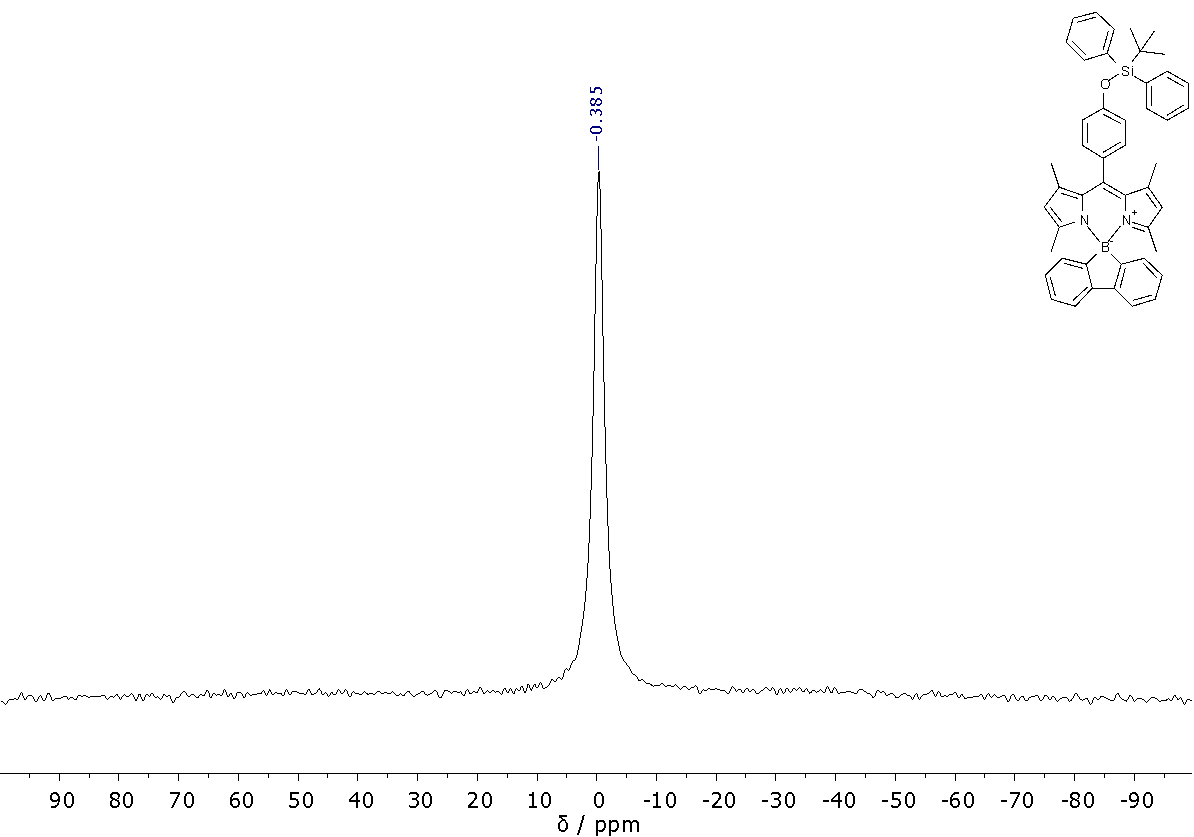


1. ^11^B NMR (96 MHz) spectra of **2** in CDCl_3_.


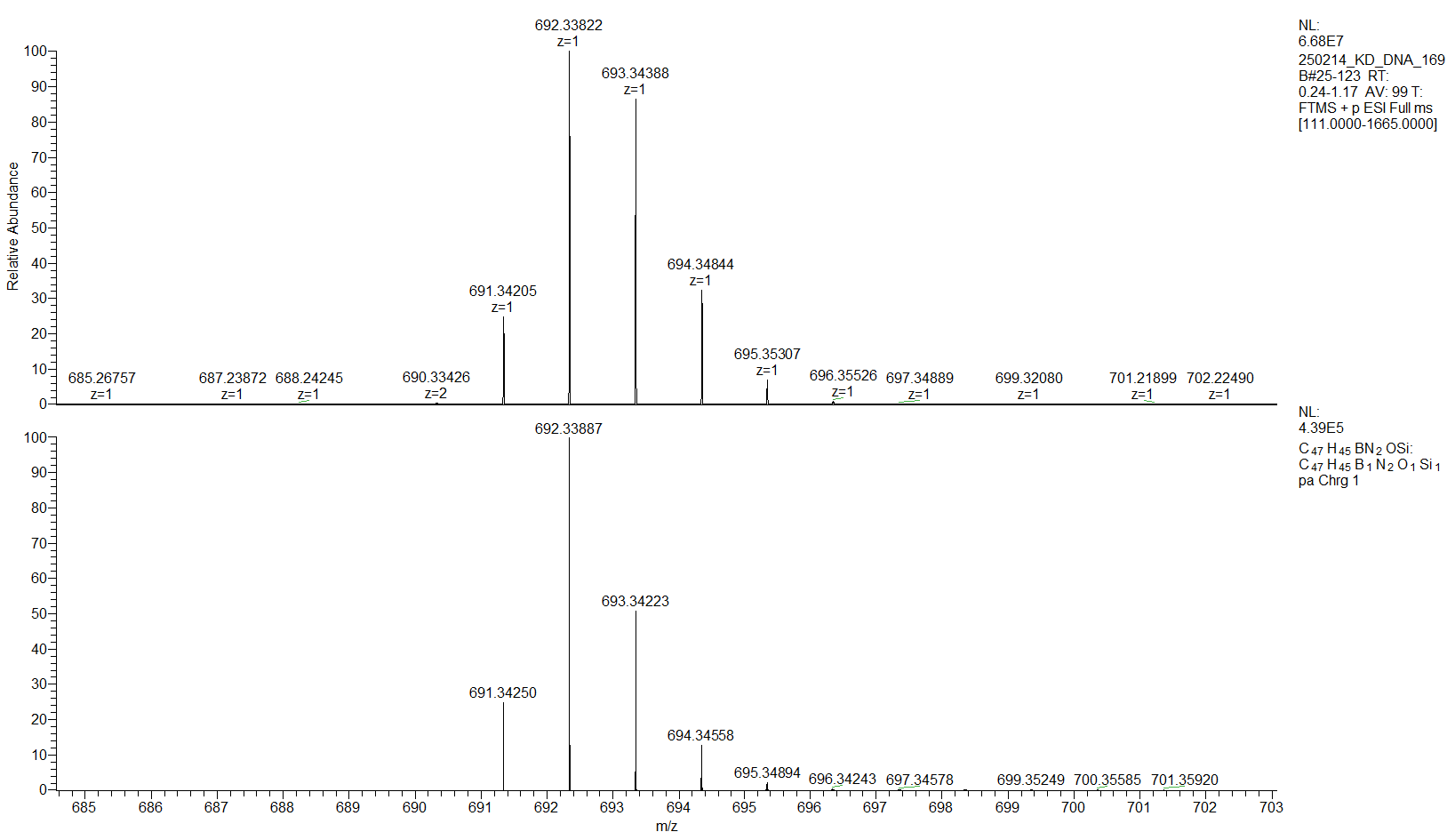


1. HRMS spectrum (ESI, positive ion mode) of **2**. The calculated spectrum of the formula C_47_H_45_BN_2_OSi^+^ [M^+^] is given in the bottom.


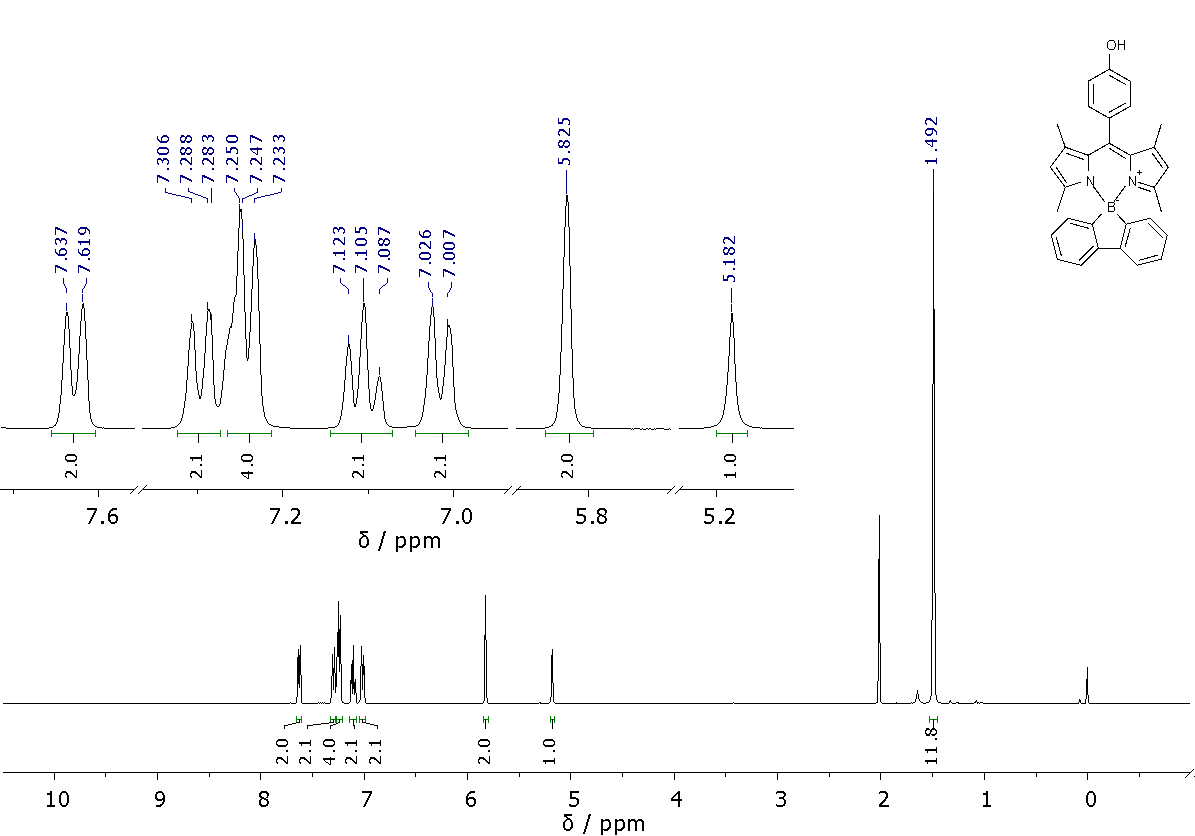


1. ^1^H NMR (400 MHz) spectra of **3** in CDCl_3_.


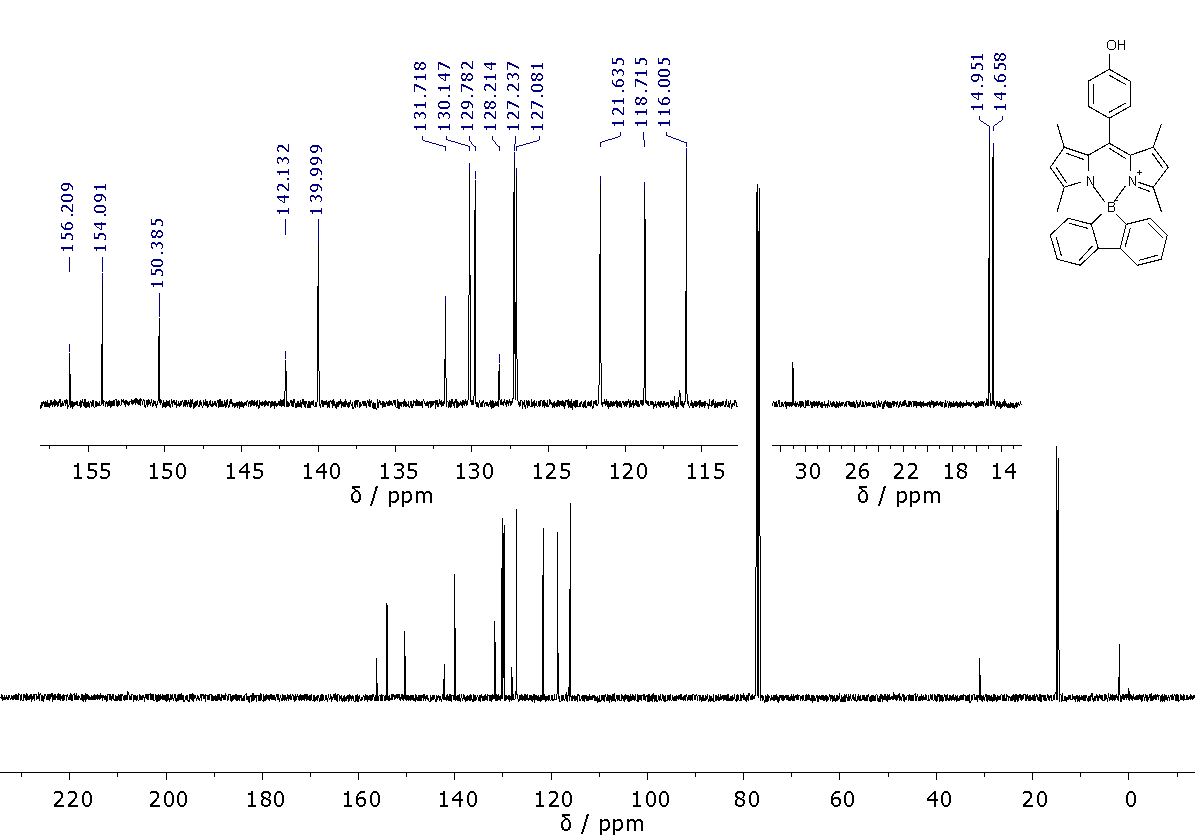


1. ^13^C{^1^H} NMR (101 MHz) spectra of **3** in CDCl_3_.


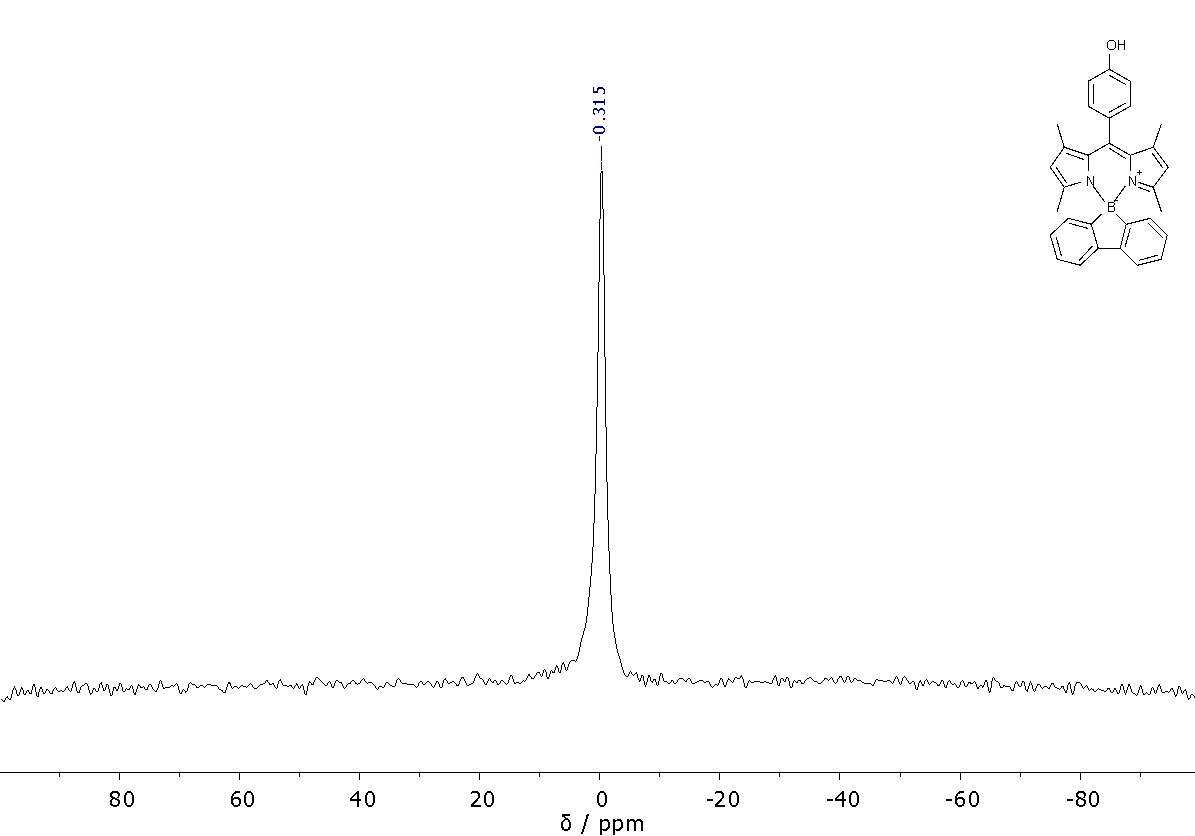


1. ^11^B NMR (96 MHz) spectra of **3** in CDCl_3_.


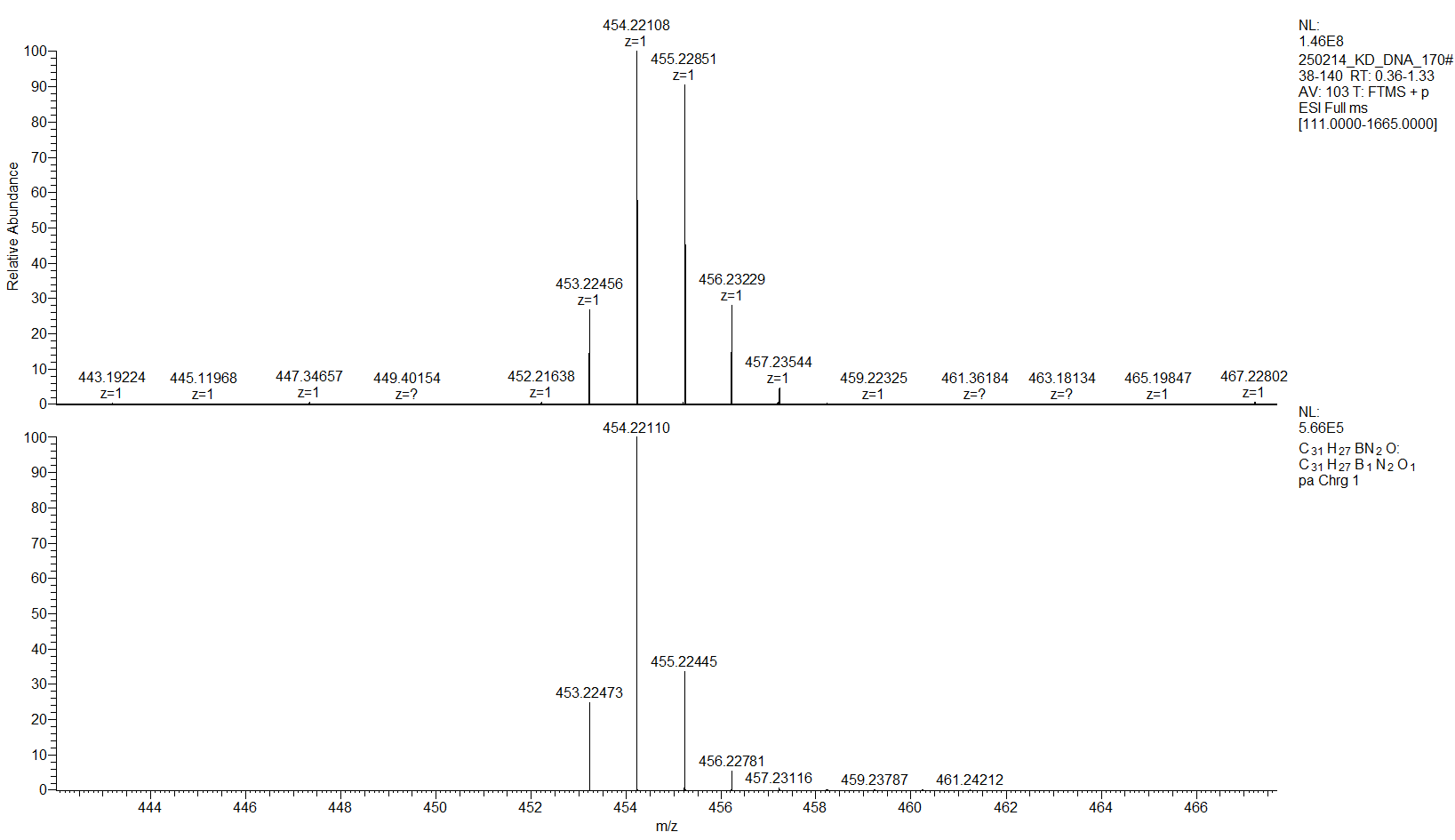


1. HRMS spectrum (ESI, positive ion mode) of **3**. The calculated spectrum of the formula C_31_H_27_BN_2_O^+^ [M^+^] is given in the bottom.


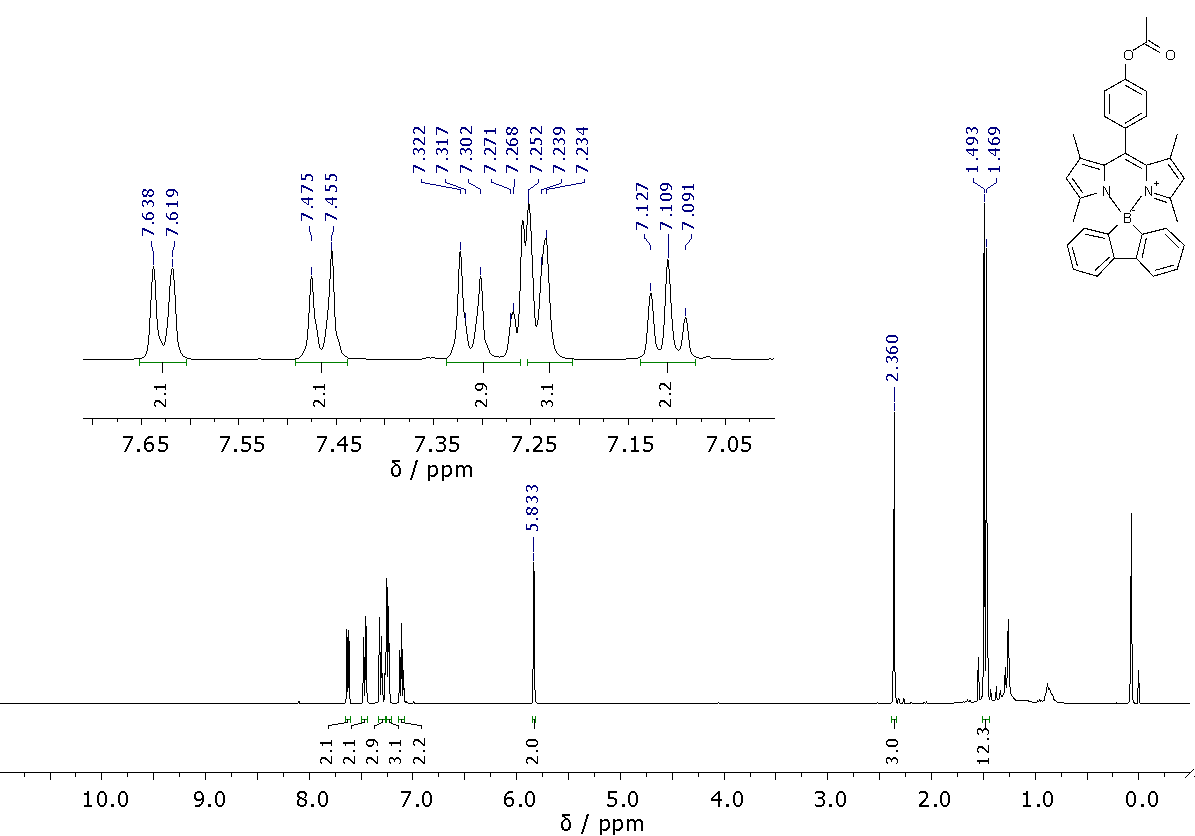


1. ^1^H NMR (400 MHz) spectra of **ref-BDP-BF** in CDCl_3_.


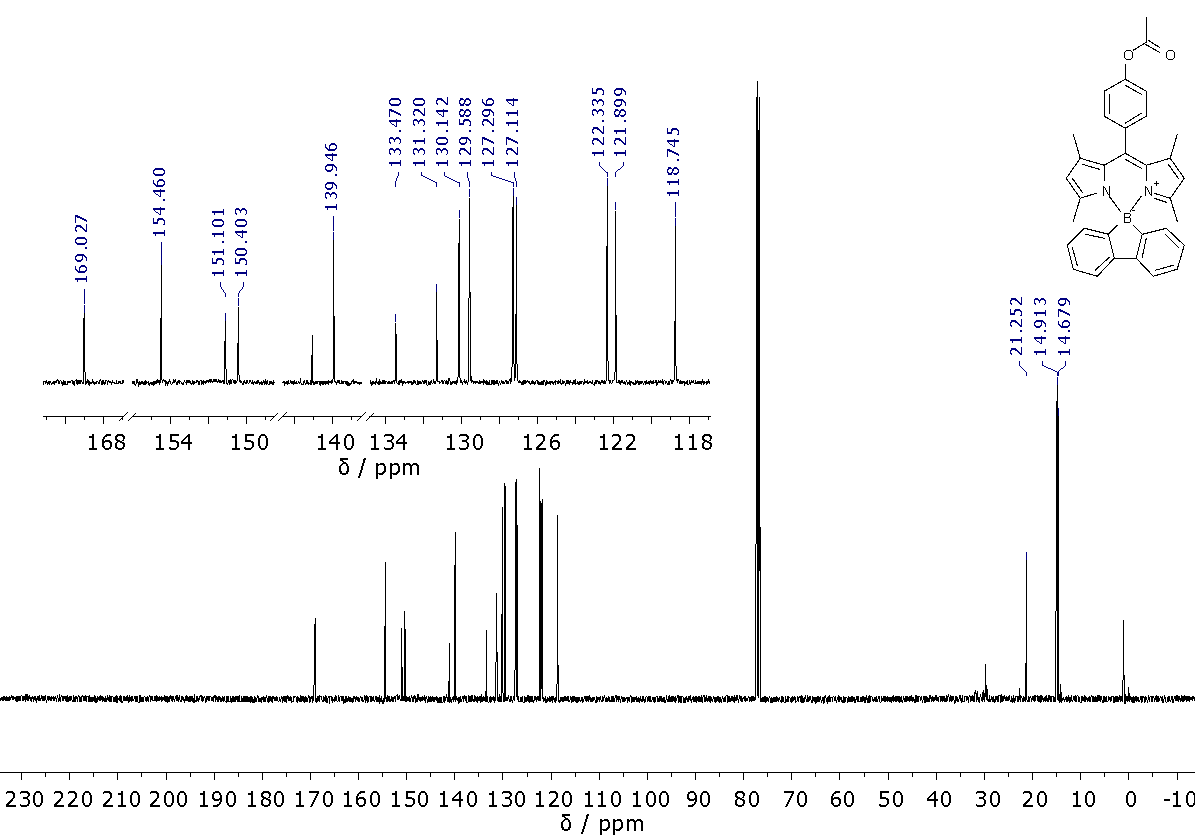


1. ^13^C{^1^H} NMR (100 MHz) spectra of **ref-BDP-BF** in CDCl_3_.


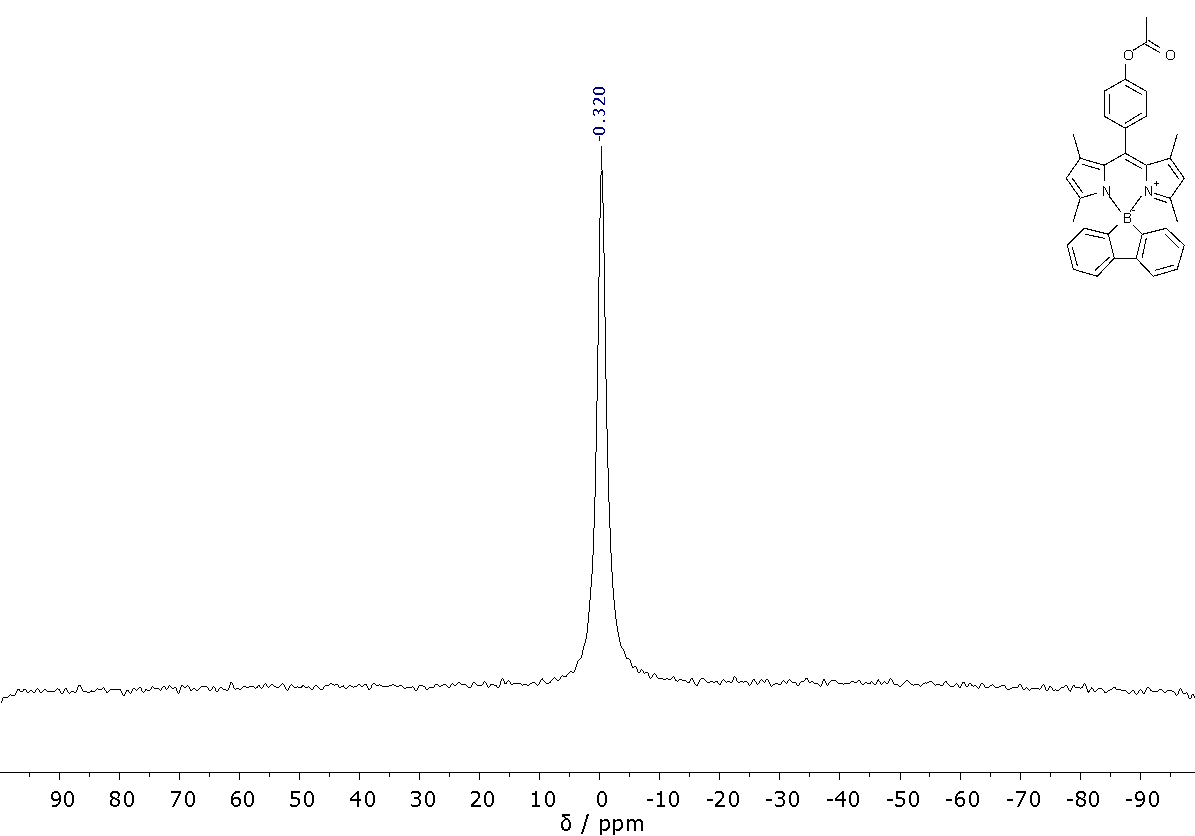


1. ^11^B NMR (96 MHz) spectra of **ref-BDP-BF** in CDCl_3_.

**
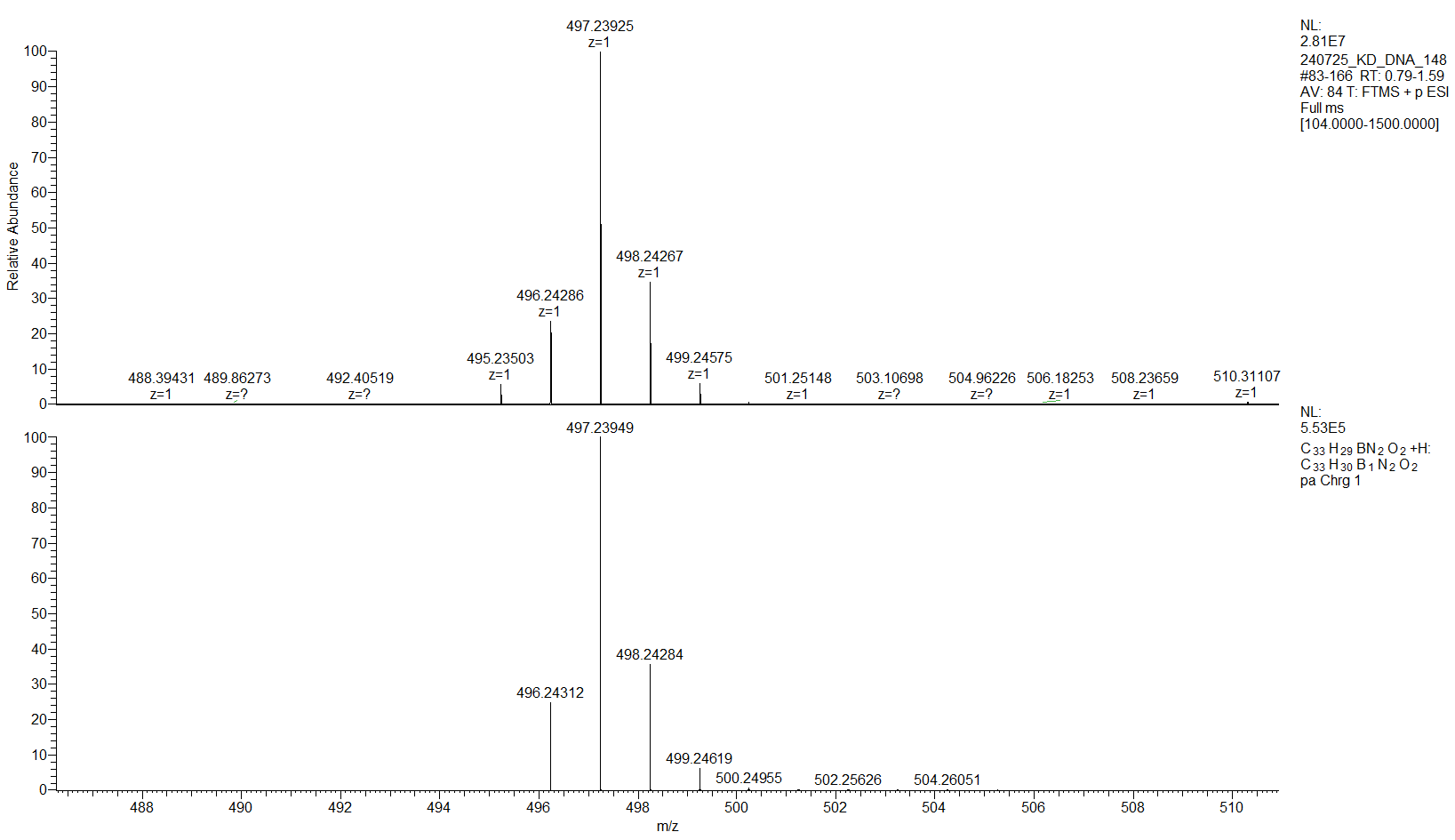
**

1. HRMS spectrum (ESI, positive ion mode) of **ref-BDP-BF**. The calculated spectrum of the formula C_33_H_29_BN_2_O_2_^+^ [MH^+^] is given in the bottom.


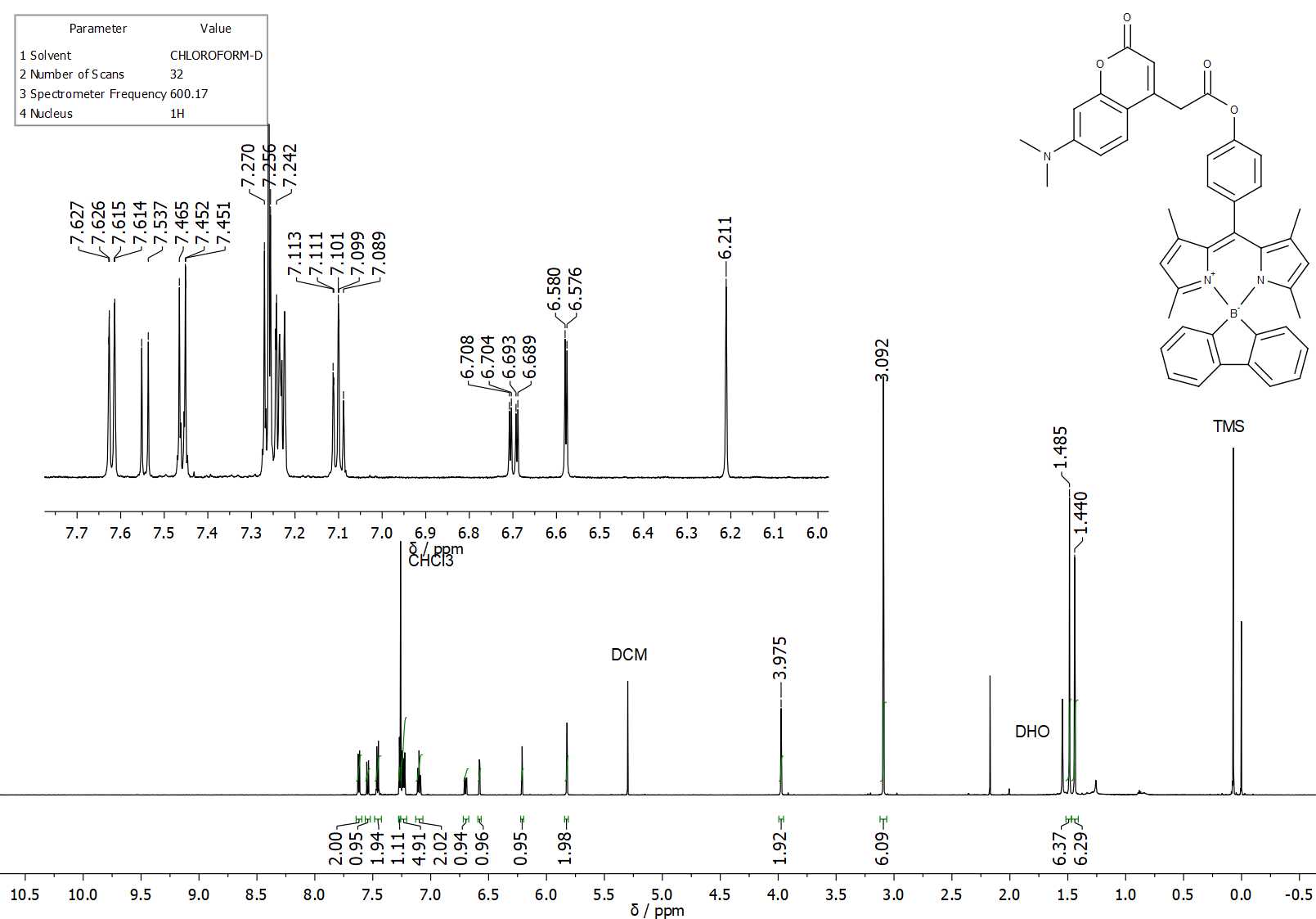


1. ^1^H NMR (600 MHz) spectra of **COU-BDP-BF** in CDCl_3_.


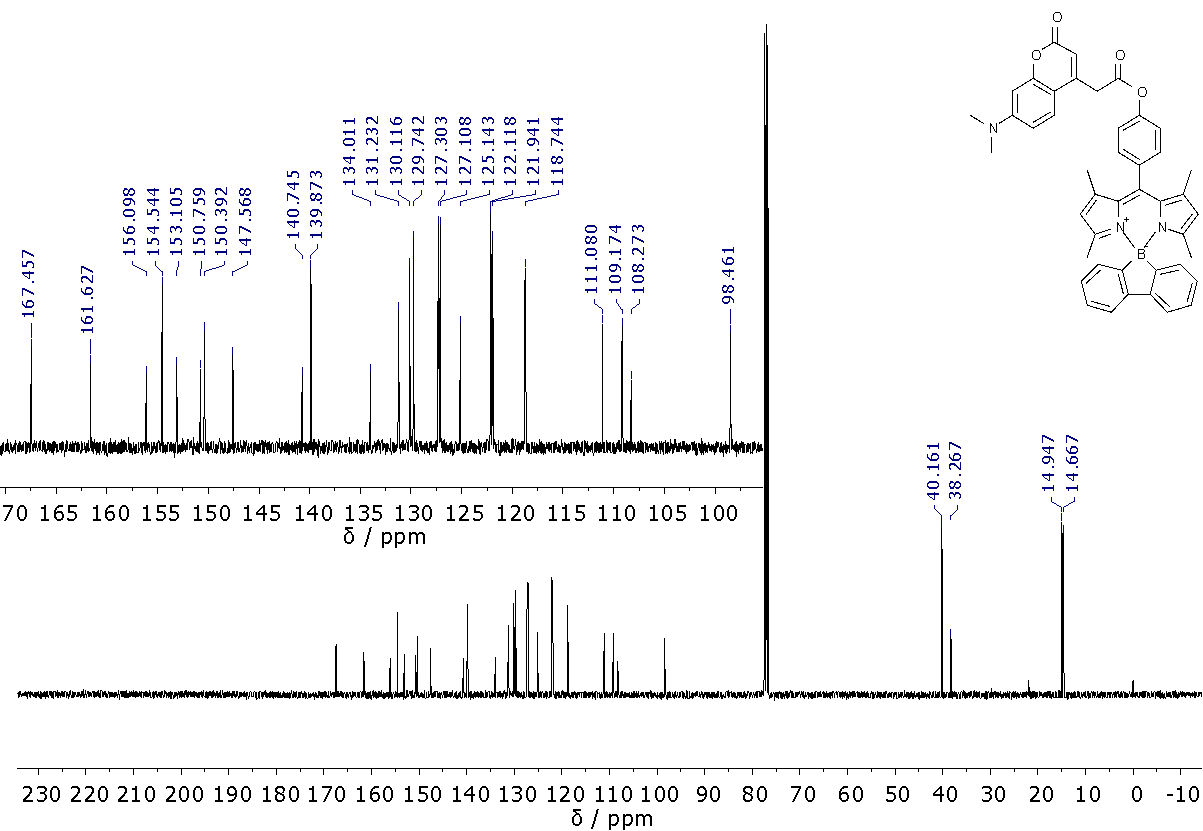


1. ^13^C{^1^H} NMR (100 MHz) spectra of **COU-BDP-BF** in CDCl_3_.


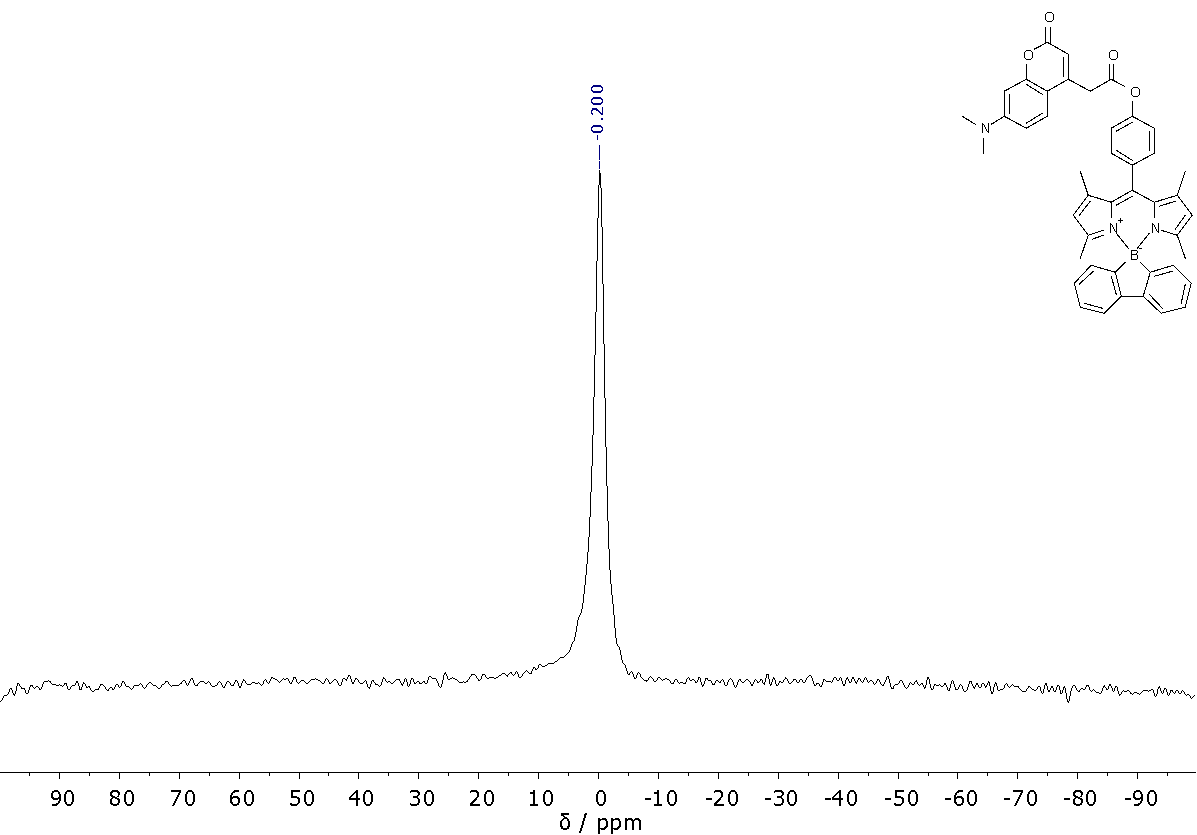


1. ^11^B NMR (96 MHz) spectra of **COU-BDP-BF** in CDCl_3_.


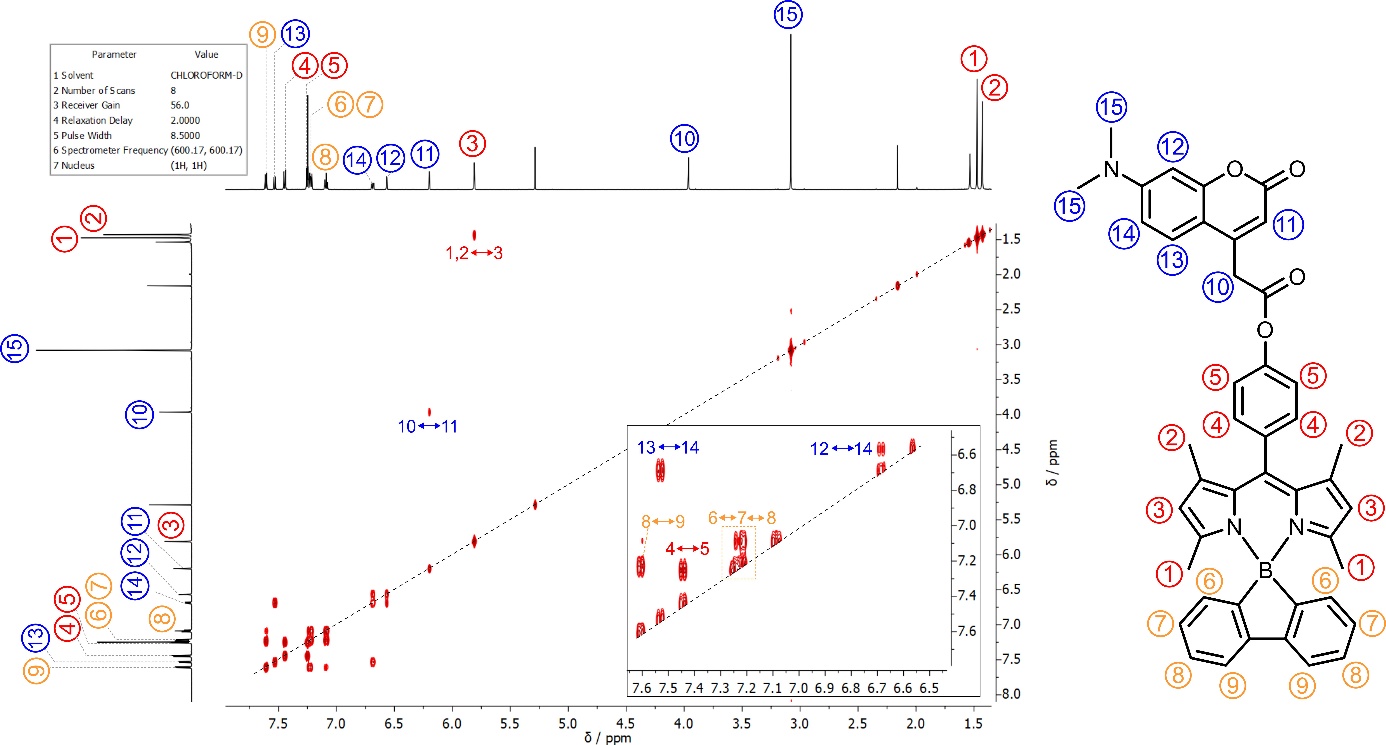


1. ^1^H-^1^H COSY (600 MHz, 600 MHz) spectrum of **COU-BDP-BF** in CDCl_3_.


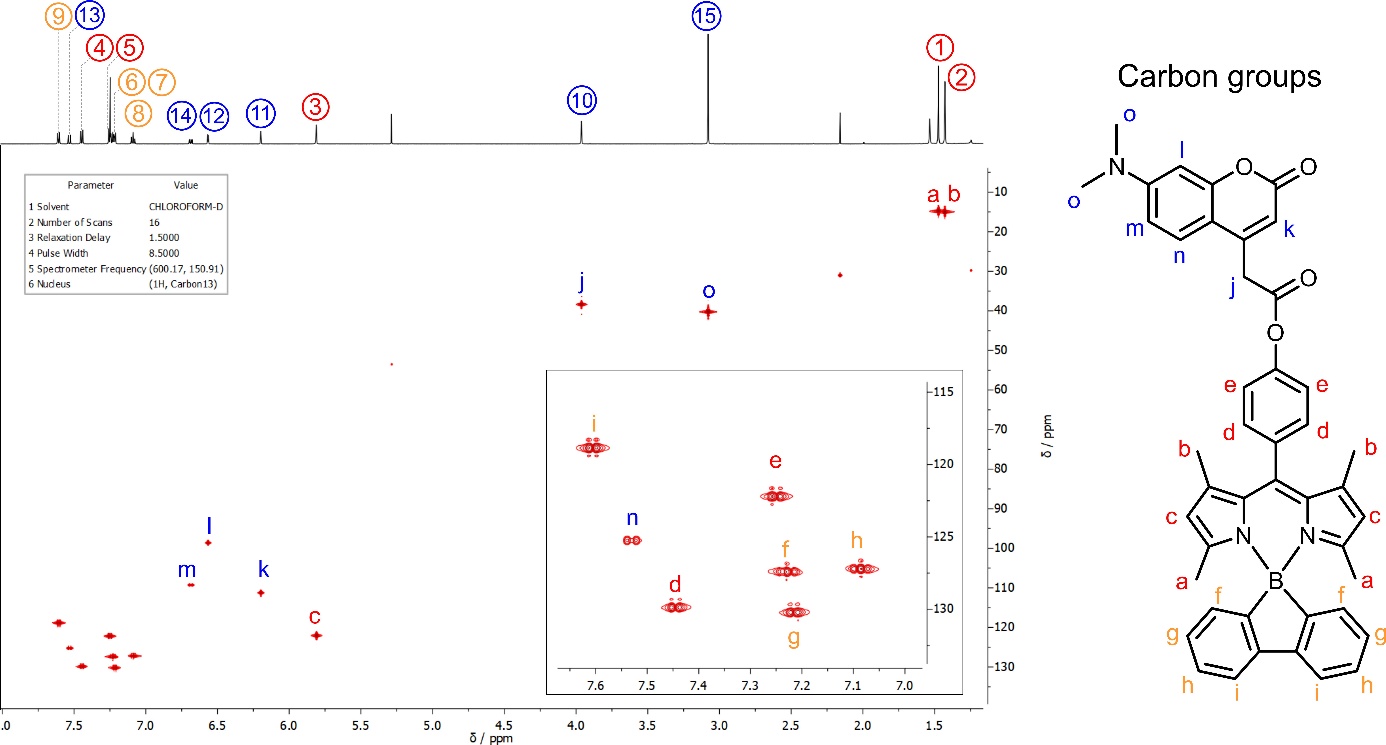


1. ^1^H-^13^C HSQC (600 MHz, 150 MHz) spectrum of **COU-BDP-BF** in CDCl_3_.

**
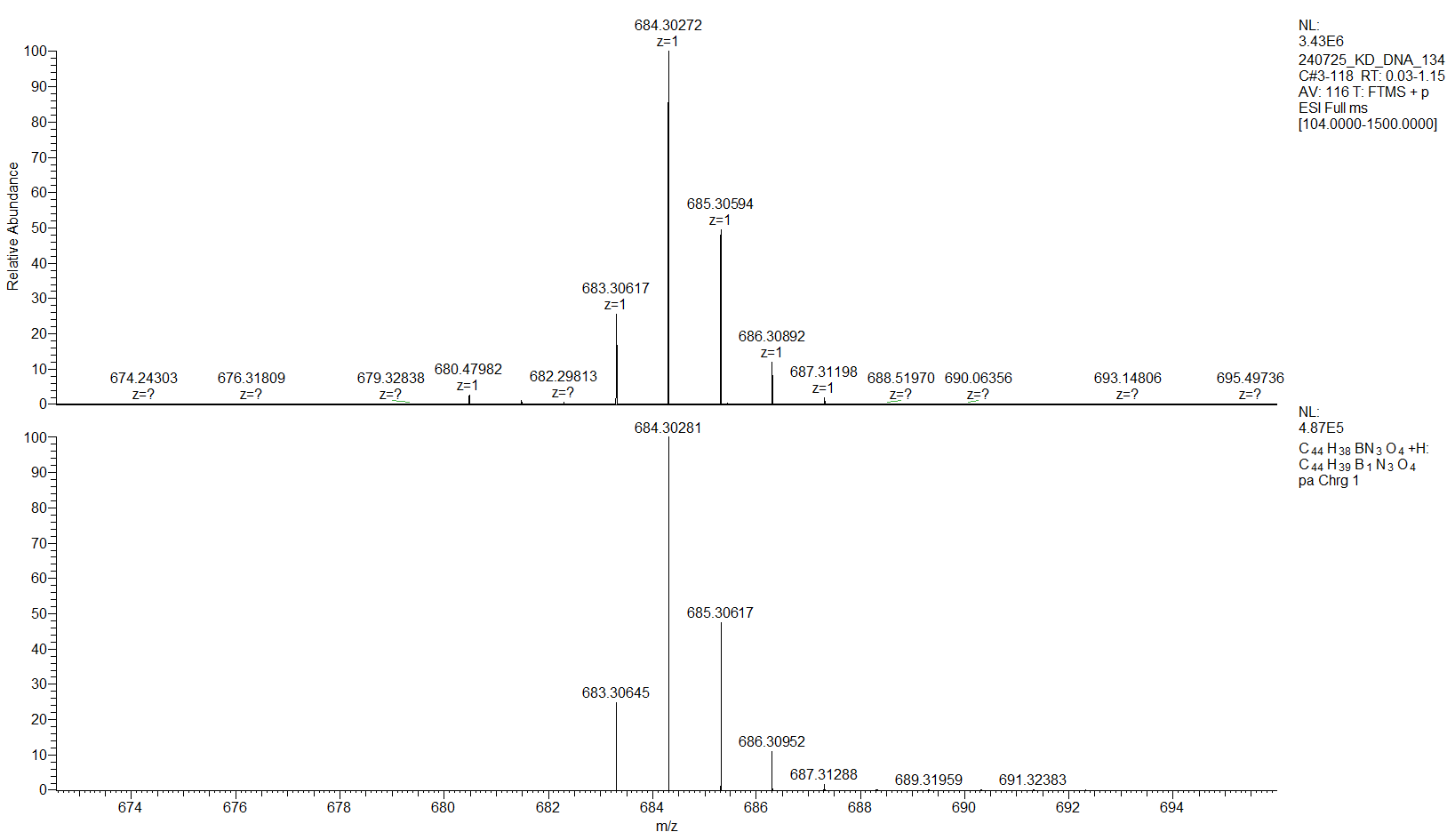
**

1. HRMS spectrum (ESI, positive ion mode) of **COU-BDP-BF**. The calculated spectrum of the formula C_44_H_38_BN_2_O_4_^+^ [MH^+^] is given in the bottom.

# References for Supporting Information

(1) Song, H. Y.; Ngai, M. H.; Song, Z. Y.; MacAry, P. A.; Hobley, J.; Lear, M. J. Practical Synthesis of Maleimides and Coumarin-Linked Probes for Protein and Antibody Labelling via Reduction of Native Disulfides. *Org. Biomol. Chem.* **2009**, *7* (17), 3400. https://doi.org/10.1039/b904060a.

(2) Clarke, K. M.; Mercer, A. C.; La Clair, J. J.; Burkart, M. D. In Vivo Reporter Labeling of Proteins via Metabolic Delivery of Coenzyme A Analogues. *J. Am. Chem. Soc.* **2005**, *127* (32), 11234–11235. https://doi.org/10.1021/ja052911k.

(3) Dhiman, S.; Kour, R.; Kaur, S.; Singh, P.; Kumar, S. Mitochondria Targeted Dual-Fluorescent Probe for Bio-Imaging Viscosity and F− with Different Fluorescence Signals. *Bioorganic Chemistry* **2022**, *129*, 106169. https://doi.org/10.1016/j.bioorg.2022.106169.
